# Efficacy, safety, pharmacokinetics, and associated microbiome changes of ibezapolstat compared with vancomycin in adults with *Clostridioides difficile* infection: a phase 2b, randomised, double-blind, active-controlled, multicentre study

**DOI:** 10.1016/j.lanmic.2025.101126

**Published:** 2025-06-11

**Authors:** Taryn A Eubank, Jinhee Jo, M Jahangir Alam, Khurshida Begum, Jacob K McPherson, ThanhPhuong M Le, Thomas D Horvath, Sigmund J Haidacher, Eugene C Poggio, Rong Lin, Corinne Seng Yue, Murray P Ducharme, Georges Koudssi, Julie Mercier, Jeffrey D Alder, Michael H Silverman, Kevin W Garey

**Affiliations:** Department of Pharmacy Practice and Translational Research, University of Houston College of Pharmacy, Houston, TX, USA (T A Eubank PharmD, J Jo PharmD, M J Alam PhD, K Begum PhD, J K McPherson PharmD, T M Le PharmD, T D Horvath PhD, Prof K W Garey PharmD); Department of Pathology and Immunology, Baylor College of Medicine, Houston, TX, USA (T D Horvath, S J Haidacher BS); Texas Children’s Microbiome Center, Department of Pathology, Texas Children’s Hospital, Houston, TX, USA (T D Horvath, S J Haidacher); Biostatistical Consulting, Lexington, MA, USA (E C Poggio PhD, R Lin MD); Learn and Confirm, Montreal, QC, Canada (C S Yue PhD, M P Ducharme PharmD); Altasciences Company, Montreal, QC, Canada (G Koudssi BS, J Mercier BS); Acurx Pharmaceuticals, Staten Island, NY, USA (J D Alder PhD, M H Silverman MD)

## Abstract

**Background:**

*Clostridioides difficile* infection is a common health-care-associated and community-acquired disease with few antibiotic treatment options. We aimed to assess the safety, efficacy, pharmacokinetics, and associated microbiome changes of ibezapolstat, an antibiotic that inhibits the PolC-type DNA polymerase III α subunit C, versus vancomycin for the treatment of *C difficile* infection in adults.

**Methods:**

This was a phase 2b, randomised, double-blind, active-controlled study conducted at 15 centres, primarily outpatient clinics and hospitals, in the USA. Adults aged 18–90 years, with signs and symptoms of *C difficile* infection and a positive toxin stool test were recruited. Participants were randomly assigned (1:1) with block assignment by study site using an interactive web response system to receive oral ibezapolstat (450 mg twice daily) or oral vancomycin (125 mg every 6 h) for 10 days. Masking was achieved by over-encapsulation of both study drugs (ibezapolstat and vancomycin) and placebo into identically sized capsules. Participants were excluded if they had received more than 24 h of treatment with oral vancomycin, fidaxomicin, or metronidazole for the current episode of *C difficile* infection before the first dose of study drug or any other antibacterial therapy within 48 h, had had more than three episodes of *C difficile* infection in the previous 12 months, or had had more than one previous episode in the past 3 months (excluding the current episode). The primary efficacy endpoint was initial clinical cure maintained for at least 48 h after the end of treatment. All individuals with *C difficile* infection who met inclusion and exclusion criteria, were randomly assigned, and were administered at least one dose of study drug were included in the efficacy analysis. The safety and tolerability of ibezapolstat was assessed in all individuals who were administered at least one dose of study drug. This study is registered with ClinicalTrials.gov, NCT04247542.

**Findings:**

Between March 12, 2021, and Oct 27, 2023, 39 individuals were assessed for eligibility, 32 of whom were recruited and randomly assigned to ibezapolstat (n=18) or vancomycin (n=14). Two participants were excluded from the efficacy analysis: one participant in the ibezapolstat group withdrew consent before receiving the study drug and another was identified after random assignment as having an exclusion criterion. The primary efficacy analysis included 16 participants in the ibezapolstat group and 14 in the vancomycin group; 24 (80%) participants were female and six (20%) were male. 15 (94%) of 16 participants in the ibezapolstat group had initial clinical cure compared with 14 (100%) of 14 participants in the vancomycin group (treatment difference −6·3% [95% CI −30·7 to 19·4]; p=1·0). Ibezapolstat was well tolerated with a safety profile similar to vancomycin. No drug-related serious adverse events, drug-related treatment withdrawal, or treatment-related deaths occurred in either group.

**Interpretation:**

Ibezapolstat is a Gram-positive selective spectrum antibiotic that shows potential in the treatment of initial *C difficile* infection and prevention of recurrence. Further clinical development is warranted.

**Funding:**

Acurx Pharmaceuticals.

## Introduction

*Clostridioides difficile* is the most common health-care-related pathogen in the USA, causing an estimated 453 000 incident cases of *C difficile* infection annually and accounting for 29 300 deaths.^1,2^ In Europe, approximately 198 526 cases are reported annually in acute care hospitals, with a large number of diagnoses missed each year due to a lack of clinical suspicion, especially in community-acquired cases.^3^ *C difficile* infection is associated with substantial mortality and morbidity, including adverse effects on quality of life.^4^ Current American and European treatment guidelines recommend two antibiotics for treatment of *C difficile* infection—oral vancomycin or fidaxomicin.^5–8^ Vancomycin is most frequently used, with initial clinical cure of 70–92% and sustained clinical cure of 42–71%.^9,10^ Fidaxomicin has fewer recurrences, an initial clinical cure of 84%, and sustained clinical cure of 67%.^11^ Both antibiotics are associated with emerging antimicrobial resistance.^8,12,13^ *C difficile* infection recurrence is associated with increased mortality, decreased quality of life, and higher health-care costs.^14^ Recurrence of *C difficile* infection is driven by persistent disruption of the gut microbiome, typically characterised by reduced proportions of Bacillota, Bacteroidota, and Actinomycetota bacterial phyla, and an increased proportion of Pseudomonadota phylum.^10^ These changes in the gut microbiome lead to loss of colonisation resistance as key bacteria essential for metabolism of bile acid are eliminated, resulting in elevated primary bile acid levels that facilitate *C difficile* spore germination and onset or recurrence of *C difficile* infection.^15^ New therapies for *C difficile* infection treatment should ideally target *C difficile* with a novel mechanism of action, sparing commensal bacteria responsible for providing colonisation resistance against *C difficile*.^16,17^ This spectrum of activity would allow for eradication of *C difficile* without treatment-associated dysbiosis that allows for further spore germination and recurrent *C difficile* infection after completion of antibiotic therapy.

Ibezapolstat (formerly ACX-362E) is a first-in-class inhibitor of the bacterial protein PolC.^18^ Uniquely, *polC* is found only in the genomes of low G+C Gram-positive bacteria in the Bacillota phylum, such as *C difficile*. This contrasts with the absence of *polC* in the genomes of Bacteroidota, Actinomycetota, and Pseudomonadota, giving ibezapolstat a phylogenetically restricted target and a narrow spectrum of activity. In an open-label, phase 2a, non-comparator study, ibezapolstat showed a narrower-than-expected spectrum of activity for some Bacillota subtaxa, resulting in an increased proportion of Lachnospiraceae and Oscillospiraceae, key taxa in metabolism of bile acid.^19^ In the same study, 100% of participants (ten of ten) had sustained clinical cure, ibezapolstat was well tolerated with no clinically significant adverse events, and favourable pharmacokinetics were observed. We aimed to assess the efficacy, safety, pharmacokinetics, and associated microbiome changes of ibezapolstat compared with vancomycin for the treatment of *C difficile* infection in adults.

## Methods

### Study design

This phase 2b, randomised, double-blind, active-controlled study was conducted at 15 centres, primarily outpatient clinics and hospitals, in the USA. Ethics approval was obtained from the institutional review board at each centre. The study adhered to the ethical principles set forth in the Declaration of Helsinki and followed all principles of Good Clinical Practice. This study is registered with ClinicalTrials.gov, NCT04247542, and is completed.

### Participants

Eligible participants were aged 18–90 years, with diarrhoea (three or more unformed bowel movements defined as type 5–7 on the Bristol Stool chart within a 24-h period) and a confirmed diagnosis of mild or moderate *C difficile* infection per the Infectious Diseases Society of America/Society of Healthcare Epidemiology *C difficile* clinical guidelines by a positive *C difficile* free toxin test within 24 h before treatment.^20^ Individuals were not enrolled if they had received more than 24 h of treatment with oral vancomycin, fidaxomicin, or metronidazole for the current episode of *C difficile* infection before the first dose of study drug, if they had received any other antibacterial therapy within 48 h before the first dose of study drug, had more than three episodes of *C difficile* infection in the previous 12 months, or had more than one previous episode in the past 3 months (excluding the current episode). Use of probiotics, antidiarrhoeal drugs, and antiperistaltic drugs was prohibited. Participant sex was self-reported (male or female). Complete eligibility criteria are in the study protocol (appendix p 5). All participants provided written informed consent.

### Randomisation and masking

Participants were randomly assigned (1:1) by block randomisation by study site to receive either ibezapolstat or vancomycin using an interactive web response system. Participants, investigators, and study personnel were masked to the treatment allocation until the database was locked. Masking was achieved by over-encapsulation of both study drugs (ibezapolstat and vancomycin) and a placebo with identically sized capsules. Participants were given blister packs that included study drug and placebo to maintain masking.

### Procedures

Ibezapolstat 450mg (three capsules, 150mg each) was given orally twice daily for 10 days, with participants also receiving a dose of placebo twice daily with the vancomycin dosing schedule. Vancomycin 125 mg (one capsule) was given orally four times daily for 10 days, along with two placebo capsules (twice daily). Participants were assessed for clinical response to treatment 2 days after the end of therapy (day 12±1), at a follow-up study visit to assess for sustained clinical cure (day 38±2), and, for a subset of participants who volunteered for long-term follow-up, at two long-term follow-up visits (days 56±2 and day 84±2). Participants were given a stool diary to be filled out daily during the study. Stool samples were collected at baseline, every other day until the evaluation for initial clinical cure, and at follow-up visits. Blood samples were collected at baseline and after day 5 of treatment for safety and plasma pharmacokinetic assessments. A study schema is in the appendix (p 2).

### Outcomes

The coprimary endpoints were initial clinical cure of *C difficile* infection, defined as resolution of diarrhoea in the 24-h period before the end of treatment, and maintained for at least 48 h after the end of treatment and safety and tolerability of ibezapolstat administered every 12 h for 10 days for treatment of *C difficile* infection.

The secondary endpoints were ibezapolstat systemic exposure, faecal concentration of ibezapolstat during treatment, and the incidence of sustained clinical cure, defined as initial clinical cure with no recurrence of *C difficile* infection within 28 days (±2 days) after the end of treatment.

Exploratory endpoints were the effect of ibezapolstat versus vancomycin on relative and quantitative changes to the faecal microbiome and stool bile acids; time to resolution of diarrhoea, defined as the interval from treatment onset to the first formed bowel movement (Bristol stool chart score <5) not followed by an unformed bowel movement within the next 24 h; and incidence of extended clinical cure in participants in the extended follow-up period, 84 days after the end of treatment.

Safety evaluations included adverse event assessments, physical examinations, vital signs, clinical laboratory tests (chemistry, haematology, and urinalysis), and electrocardiography. Safety endpoints for all participants were recorded, including nature, frequency, and severity of adverse events. Adverse events were assessed from enrolment and at each visit. Adverse events were classified according to the Medical Dictionary for Regulatory Activities (version 15.0) and evaluated for severity (mild, moderate, or severe) and causality (unrelated, possibly related, or probably related to the study drug) by site investigators.

In the pharmacokinetic assessment, plasma samples were obtained 2 h and 4 h after administration of the first daily ibezapolstat dose on days 1 and 4. Stool samples were collected at baseline, every other day during treatment, and at follow-up visits. Ibezapolstat plasma and stool concentrations were quantified by liquid chromatography-tandem mass spectrometry (Altasciences, Laval, QC, Canada).^21^

For microbiology and ribotyping, stool samples were enriched in brain–heart infusion broth with 0·05% sodium taurocholate (Hardy Diagnostics, Santa Maria, CA, USA) and incubated anaerobically at 37°C for 48 h, pelleted and resuspended, and then plated for *C difficile* growth onto cycloserine–cefoxitin fructose agar (Anaerobe Systems, Morgan Hill, CA, USA) at 37°C under anaerobic conditions for an additional 48 h.^22^ Isolates were confirmed as *C difficile* based on growth characteristics, morphology, and PCR confirmation of *C difficile* toxin genes. *C difficile* isolates were characterised by PCR-based ribotyping.^23^

DNA extraction and microbiome sequencing of the V1–V3 region of the 16S ribosomal RNA gene was done using the MiSeq system (Illumina, San Diego, CA, USA) following the manufacturer’s directions as previously described.^19^ Sequencing yielding at least 15 000 reads per sample was included for analysis. Bile acid concentrations were measured via liquid chromatography-tandem mass spectrometry as previously described and normalised to the corresponding stool sample weight.^24^

### Statistical analysis

Up to 72 participants were initially planned for random assignment in a non-inferiority trial with an endpoint of initial clinical cure (expected cure rate 80%), one-sided normal approximation, and a 1:1 treatment allocation (non-inferiority margin 0·25; power 80%). After an interim efficacy analysis with review by an independent data monitoring committee, the study was discontinued in consultation with medical and scientific advisers based on blinded observations of a high aggregate sustained clinical cure rate, no emerging safety concerns, and challenges in maintaining clinical study sites during the COVID-19 pandemic and its aftermath. The blinded clinical observations of a high overall success rate obviated the need for a non-inferiority assessment. The sponsor determined, in consultation with its clinical and statistical experts, that presenting clinical cure rates for the primary efficacy endpoint was the most appropriate representation for the clinical activity of ibezapolstat in treating *C difficile* infection.

For the phase 2b study, the intention-to-treat population included all randomly assigned participants. The per-protocol population included all participants in the intention-to-treat population who took at least one dose of study medication, met all inclusion and exclusion criteria, and had no major protocol deviation. All analyses were done using the per-protocol population except for safety analyses, which were done in all patients who received at least one dose of study drug. Baseline characteristics and safety data were tabulated using summary statistics. For the primary endpoint of initial clinical cure, the mean difference in response rate, 95% exact CIs for a binomial percentage, and Fisher’s exact p value between treatment groups (ibezapolstat and vancomycin) were calculated. The same evaluation was done for the secondary endpoint of sustained clinical cure. The exploratory endpoint of time to resolution of diarrhoea was evaluated using Kaplan–Meier curves to estimate median time to resolution of diarrhoea during treatment starting at day 1 of therapy for each treatment group. Microbiome abundance operational taxonomic units were generated using CLC Genomics software (version 12.0.3; Qiagen, Germantown, MD, USA) and analysed using vegan R package (version 2.6.6.1)^25^ and visualised using ggplot2 R package (version 3.5.1).^26^

Bacterial taxa significantly associated with ibezapolstat versus vancomycin were assessed using MaAsLin2.^27^ Primary and secondary bile acid changes from baseline and secondary-to-primary bile acid ratio over time in response to ibezapolstat or vancomycin were summarised and graphically visualised.

### Role of the funding source

The funder of the study was responsible for the study design and data collection. JDA and MHS are coauthors on this Article and also paid consultants of the funder—they had a role in data analysis, data interpretation, and writing of the report. The corresponding author had final responsibility for data interpretation and writing of the report.

## Results

**Figure 1:**
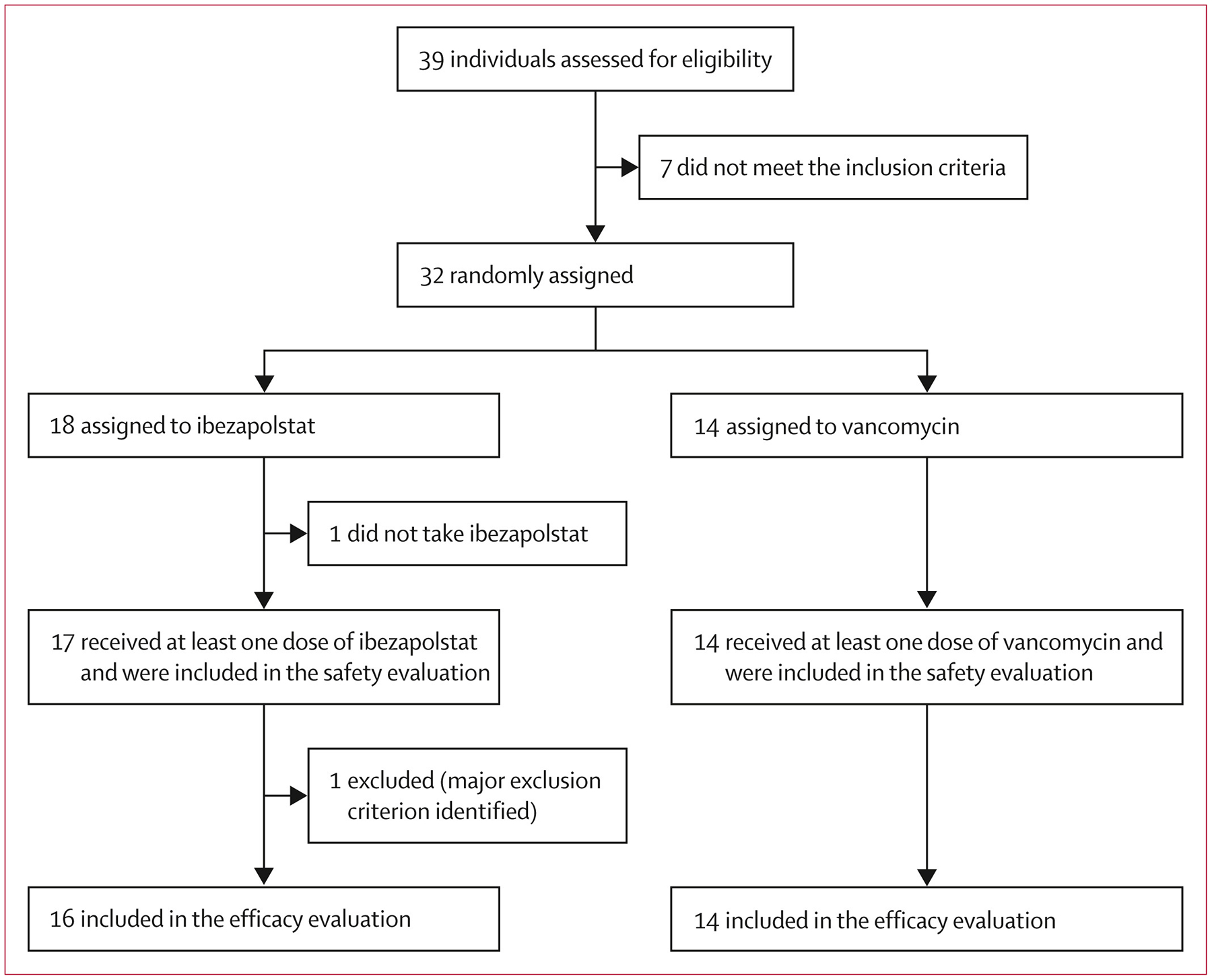
Trial profile

Between March 12, 2021, and Oct 27, 2023, 39 individuals were assessed for eligibility, 32 of whom were recruited and randomly assigned to ibezapolstat (n=18) or vancomycin (n=14; figure 1). One participant in the ibezapolstat group withdrew consent before receiving any study drug and one additional participant in the same group was found to have an exclusion criterion (irritable bowel syndrome) after starting ibezapolstat, which constituted a major protocol deviation. All participants who received at least one dose of study drug were included in the safety population (n=31). All participants who received at least one dose of study drug and met eligibility criteria (n=30) were assessed for clinical outcomes and secondary objectives. Baseline characteristics were similar between the ibezapolstat and vancomycin groups in the per-protocol population; 24 (80%) participants were female and six (20%) were male (table 1).

**Figure 2:**
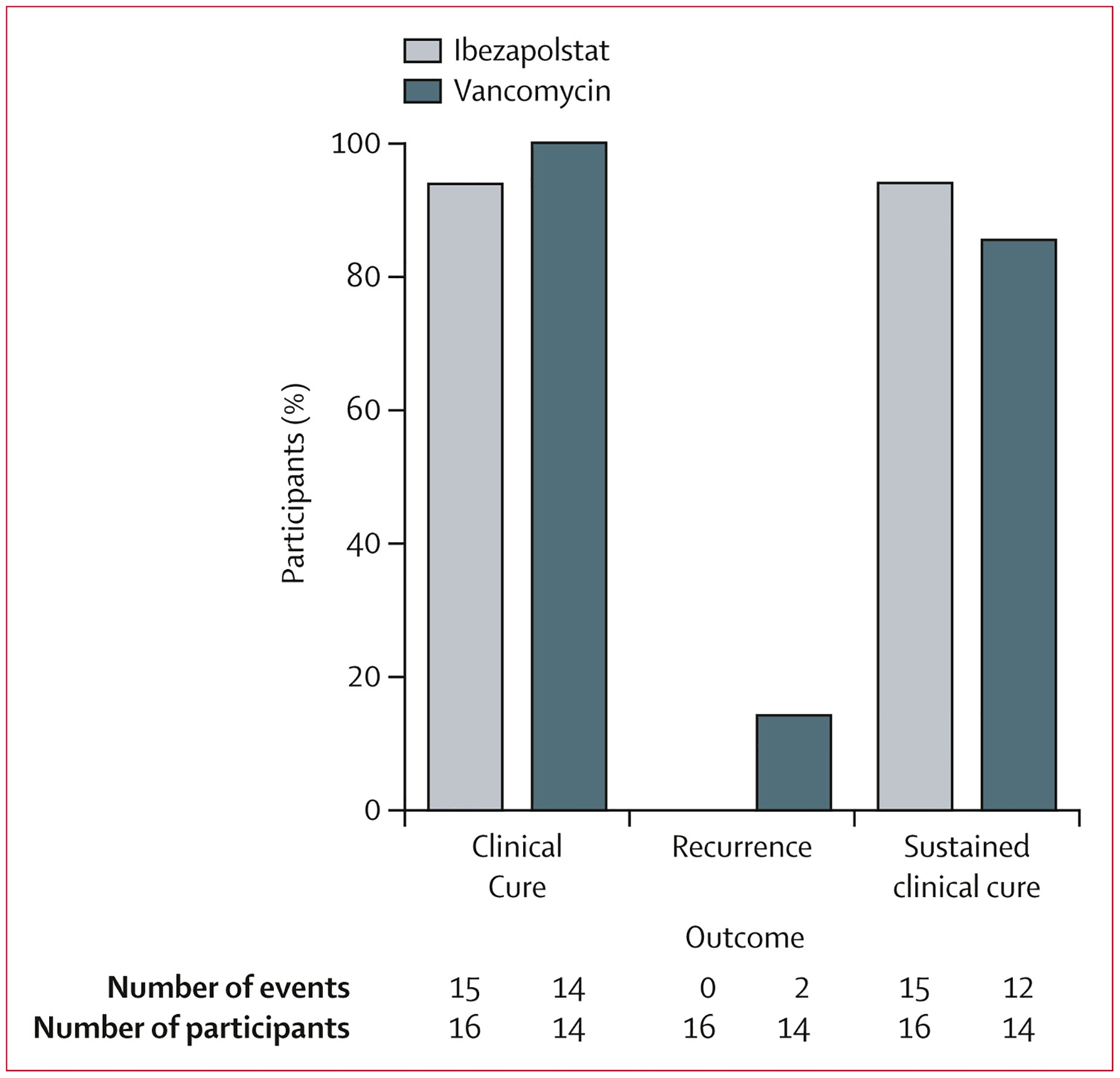
Efficacy analysis in the per-protocol population

15 (94%) of 16 participants in the ibezapolstat group had initial clinical cure compared with 14 (100%) of 14 participants in the vancomycin group (treatment difference −6·3% [95% CI −30·7 to 19·4]; p=1·0; figure 2). No participants in the ibezapolstat group had a recurrence assessed on day 28 after the end of treatment compared with two (14%) in the vancomycin group.

15 (94%) participants in the ibezapolstat group had sustained clinical cure compared with 12 (86%) in the vancomycin group (treatment difference 8⋅0% [95% CI −19·4 to 38·0]; p=0·59). In the exploratory analysis, median time to resolution of diarrhoea was 7 days (95% CI 4–8) in the ibezapolstat group and 10 days (6–11) in the vancomycin group (log-rank p=0·57; appendix p 2). In the exploratory analysis of extended clinical cure, there were no recurrences of *C difficile* infection among the five participants who had sustained clinical cure and who were followed up for 84 days after the end of treatment.

For the primary safety analysis, both treatments were well tolerated with no drug-related serious adverse events or treatment withdrawals (table 2). One (7%) of 14 participants in the vancomycin group had a moderate adverse event possibly related to study drug (headache). Three (18%) of 17 participants in the ibezapolstat group had mild adverse events possibly related to study drugs (gastro-oesophageal reflux disease [n=2] and nausea [n=1]).

Toxigenic *C difficile* was isolated from baseline stool samples in 11 (69%) of 16 participants in the ibezapolstat group and nine (64%) of 14 in the vancomycin group. Baseline *C difficile* ribotypes are shown in table 1. On day 3, one (6%) participant in the ibezapolstat group and two (14%) in the vancomycin group were positive for *C difficile*. In the vancomycin group, one (7%) additional participant on day 5 and one (7%) on day 8 had *C difficile* growth. No participants in the ibezapolstat group had *C difficile* growth after day 3 of treatment.

**Figure 3:**
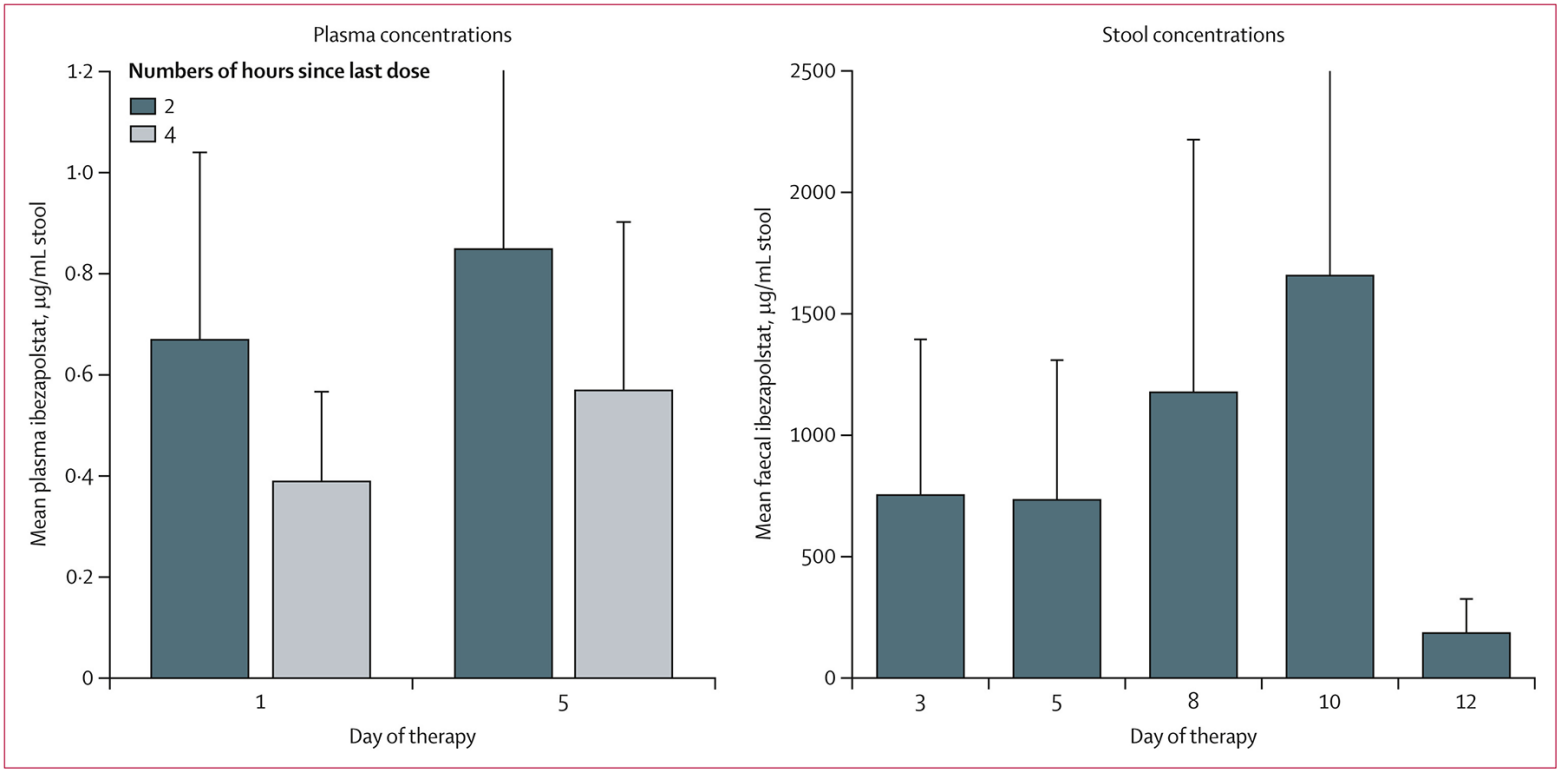
Ibezapolstat plasma and stool concentrations in the per-protocol population Data are mean (95% CI).

Plasma and stool ibezapolstat concentrations are shown in figure 3. Mean plasma ibezapolstat concentrations were relatively similar between the first day of therapy (0·63 μg/mL [95% CI 0·30–1·00] at 2 h and 0·36 μg/mL [0·20–0·57] at 4 h) and on day 5 of continuous dosing (0·79 μg/mL [0·30–1·90] at 2 h and 0·53 μg/mL [0·23–0·90] at 4 h), with little systemic accumulation. The mean stool ibezapolstat concentration was 750 μg/g (95% CI 109–1391) on the third day of dosing, 734 μg/g (156–1311) on day 5, 1175 μg/g (133–2217), on day 8, 1653 μg/g (450–3757) on day 10, and 185 μg/g (51–318) on day 12, two days after ibezapolstat discontinuation.

**Figure 4:**
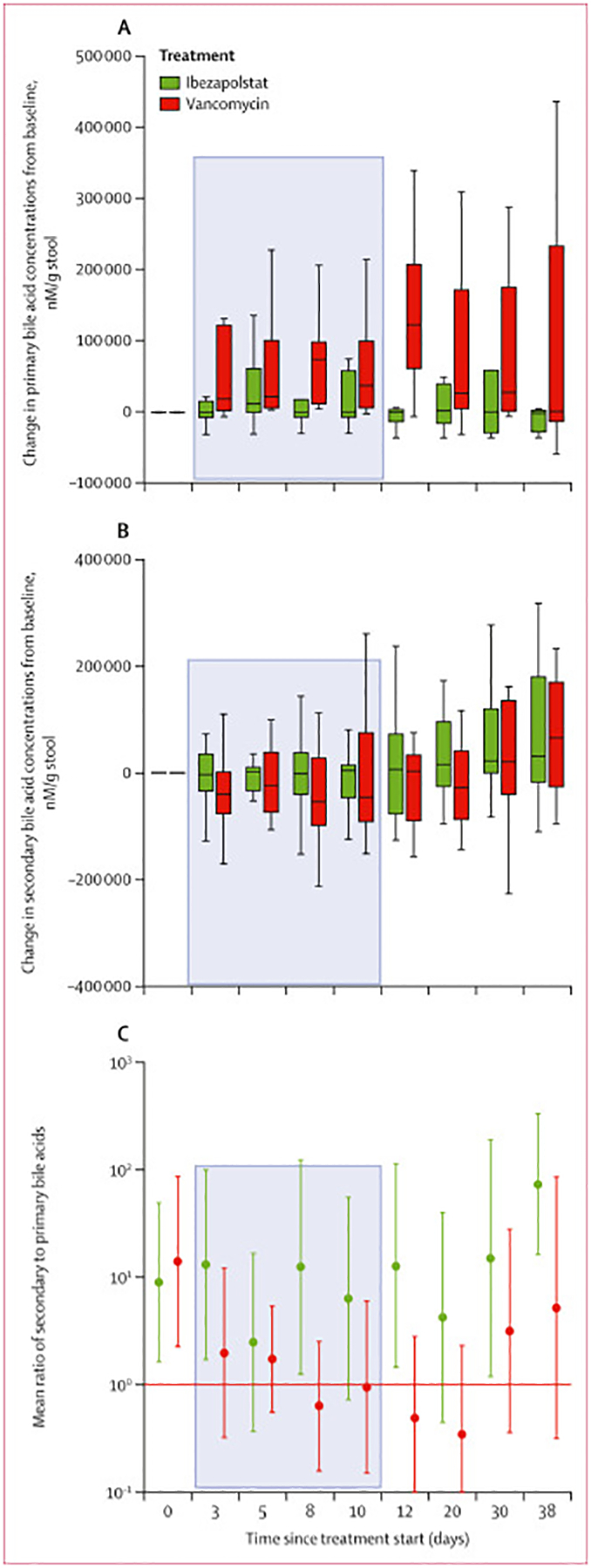
Microbiome changes in the per-protocol population (A) 16s rRNA analysis of α diversity changes (primary bile acids) assessed by either the Shannon and Simpson diversity index. (B) 16s rRNA analysis of abundance changes at the phylum and order level (secondary bile acids). (C) Ratio of secondary to primary bile acid concentrations. Grey shading shows active receipt of therapy. Error bars represent 1·5 × IQR from box edge (panels A and B) and 95% CIs (panel C).

Microbiome changes by study drug are shown in figure 4. α diversity, assessed by either the Shannon or Simpson diversity index, was similar at baseline for participants in the ibezapolstat and vancomycin groups but decreased in the vancomycin group (figure 4A). Proportional changes in bacterial taxa are shown in figure 4B. β diversity was similar at baseline for the ibezapolstat and vancomycin groups but diverged after the 10-day treatment, especially for the two participants in the vancomycin group with recurrence of *C difficile* infection (appendix p 4). MaAsLin2 analysis found that ibezapolstat treatment was associated with decreased proportions of Bifidobacteriales (Phylum: Actinomycetota), Bacteroidales (Phylum: Bacteroidota), and Clostridiales (Phylum: Bacillota) compared with vancomycin treatment (adjusted p<·05 each).

Total primary and secondary bile acid concentrations were similar between the ibezapolstat group and the vancomycin group at baseline (figure 4). Before evaluation at the end of treatment (days 1–12), total primary bile acids increased by a mean of 657 nM/g stool per day (SD 2324) and secondary bile acids increased by a mean of 1928 nM/g stool per day (1888 nM/g) compared with baseline in the ibezapolstat group. In the vancomycin group during therapy (days 1–12), total primary bile acids increased by a mean of 9023 nM/g stool per day (1931) and secondary bile acids increased by a mean of 824 nM/g stool per day (2736) compared with baseline. Primary bile acid concentrations remained greater than at baseline in the vancomycin group but not the ibezapolstat group through to follow-up on day 38, whereas secondary bile acids returned to baseline values by day 30 in both the ibezapolstat group and the vancomycin group (figure 4C). These changes resulted in more primary than secondary bile acids in more than 50% of participants in the vancomycin group by day 5 of therapy, which persisted until day 30 of follow-up.

## Discussion

In this phase 2b study, ibezapolstat showed similar efficacy to vancomycin for the treatment of *C difficile* infection in adults. One participant in the ibezapolstat group did not have initial clinical cure and two participants in the vancomycin group had recurrence of *C difficile* infection between study days 12 and 38. The mean time to resolution of diarrhoea was similar in both treatment groups. Both treatments were well tolerated, with the safety profile of ibezapolstat being consistent with results from the phase 1 and 2a studies.^19,21^ Ibezapolstat was poorly bioavailable, with high stool concentrations many orders of magnitude above the minimum inhibitory concentration for *C difficile* and plasma concentrations remaining less than 1 μg/mL for most participants (11 of 15), with no participant exceeding 4 μg/mL.^19,28^ Favourable microbiome changes in the ibezapolstat group included preservation of key taxa known to metabolise primary bile acids to secondary bile acids via the 7-α dehydroxylation pathway.^29^ Finally, bile acid analysis showed that participants in the vancomycin group had decreased primary bile acid conversion, leading to decreased ratios of secondary to primary bile acids, a shift not observed in the ibezapolstat group. Increased primary bile concentrations have been shown in the past with vancomycin use and increase the risk of recurrent *C difficile* infection.^10,30^ Taken together, these results provide further evidence that ibezapolstat reduces recurrence of *C difficile* infection and support further development of ibezapolstat in phase 3 studies.

New antibiotic development for *C difficile* infection is urgently needed. This study provides further evidence that vancomycin, although effective at reducing initial clinical symptoms of *C difficile* infection, has a major disruptive effect on the microbiome, increasing the risk of recurrence. This study is one of the first clinical studies to distinguish therapies by their effect on specific microbial taxa and bile acid homoeostasis. These data further support ibezapolstat’s mechanism of action for treatment of *C difficile* infection and highlight the limitations of vancomycin therapy. The effect of ibezapolstat on the human gut microbiome, including increased Actinomycetota, specific Eubacteriales, and Lachnospirales that contribute to short-chain fatty acid synthesis and bile acid metabolism, is consistent with observations from ibezapolstat phase 1 and 2a clinical studies. If validated in future studies, this multiomics approach to drug development for *C difficile* infection might allow for better and earlier prediction of anti-recurrence effects for antibiotics.

Although fidaxomicin is narrower in spectrum than vancomycin, relying on a single antibiotic for an infection that affects a large population is problematic, especially as fidaxomicin resistance has been reported.^31^ With a novel mechanism of action, ibezapolstat retains activity against strains non-susceptible to fidaxomicin, vancomycin, and metronidazole.^13^ In a head-to-head mouse model comparison with fidaxomicin, ibezapolstat showed a similarly narrow spectrum of microbiome disruption compared with fidaxomicin but resulted in a distinct microbiome profile in ibezapolstat-treated mice.^32^ Extension of these microbiome results to other metabolic changes in preclinical models might help inform future head-to-head clinical studies, distinguishing available therapies and enhancing our mechanistic understanding of recurrence of *C difficile* infection.

This study has limitations. The study enrolled a small number of participants and was not statistically powered to establish the typical 10% non-inferiority margin. This led to imprecision in statistical test measurements and wide CIs. However, high response rates for both ibezapolstat and vancomycin, along with no recurrence of *C difficile* infection in the ibezapolstat group, provided clinical evidence to support larger phase 3 studies. This study recruited participants with mild–moderate *C difficile* infection; future studies including participants with severe *C difficile* infection will be of value. Sustained clinical cure was evaluated 28 days after the end of treatment for all participants, with only a small subset of participants in the ibezapolstat group being evaluated beyond that timepoint. Of the five participants in the ibezapolstat group with sustained clinical cure and who were followed up long term, all had long-term cure. In future studies, larger numbers of participants will be valuable. Finally, our metabolic analyses were not powered to evaluate specific primary or secondary bile acid components. Larger studies will be needed to provide further mechanistic insights.

In conclusion, ibezapolstat was as clinically effective as vancomycin for the treatment of *C difficile* infection in adults, showed ideal pharmacokinetics for the treatment of *C difficile* infection, and showed beneficial effects on the microbiome and bile acid homoeostasis. These results support further development in phase 3 studies.

## Supplementary Material

Supplementary appendix

## Figures and Tables

**Table 1:** Demographics and baseline characteristics in the per-protocol population

	Ibezapolstat (n=16)	Vancomycin (n=14)
Age >65 years	8 (50%)	5 (36%)
Age >75 years	5 (31%)	2 (14%)
Sex		
Female	13 (81%)	11 (79%)
Male	3 (19%)	3 (21%)
Race		
White	16 (100%)	13 (93%)
Black	0	1 (7%)
Ethnicity		
Hispanic or Latino	11 (69%)	11 (79%)
Other	5 (31%)	3 (21%)
Charlson Comorbidity Index	2 (1–4)	2 (1–4)
Antibiotic use for Clostridioides *difficile* infection before starting study drug, three doses oral vancomycin	2 (13%)	2 (14%)
*C difficile* infection test used for initial diagnosis*	13 (81%)	11 (79%)
Number of unformed bowel movements at baseline	6 (4–7)	6 (4–8)
Baseline C *difficile* ribotypes†		
014–020	0	3 (27%)
027	1 (9%)	2 (18%)
106	3 (27%)	1 (9%)
002	1 (9%)	1 (9%)
116	0	1 (9%)
Other	6 (55%)	3 (27%)

Data are n (%) or median (IQR).

* All participants diagnosed using a *C difficile* free toxin test within 24 h before treatment (using *C difficile* Quik Chek Complete, Techlab).

† Not all patients had *C difficile* growth at baseline.

**Table 2:** Drug-related adverse events

	Ibezapolstat (n=17)	Vancomycin (n=14)
Drug-related serious adverse events	0	0
Drug-related treatment withdrawals	0	0
Moderate adverse event, possibly related	0	1 (7%)*
Mild adverse event, possibly related	3 (18%)†	0

Safety evaluation in all participants who received at least one dose of study drug.

* Headache.

† Two (12%) gastro-oesophageal reflux disease and one (6%) nausea.

## Data Availability

The protocol, clinical study report, and de-identified patient data will be made available to researchers with an accepted rationale by Acurx Pharmaceuticals once the study is complete and the final study report has been finalised. Access to the data can be requested from msilverman@biostrategics.com.

## Research in context

### Evidence before this study

We searched PubMed using the terms “ibezapolstat” and “ACX362E”, with no language restrictions, limited to non-review articles and a 5-year period before approval of the protocol (April, 2018 to September, 2024). The search identified seven articles describing the mechanism of action of ibezapolstat, which inhibits the PolC in low G+C Gram-positive bacteria, and the in-vitro activity against *Clostridioides difficile*, including strains with reduced susceptibility to other *C difficile*-targeted antibiotics. Among the resulting articles, a phase 1 study showed ibezapolstat to be safe, well tolerated, localised to the gastrointestinal tract, and associated with preservation of beneficial gut microbiota in humans. These findings were supported by a phase 2a, open-label, non-comparator study and showed that ten of ten participants were cured of *C difficile* infection, with no recurrence.

### Added value of this study

This phase 2b, randomised, double-blind, active-controlled study showed high rates of initial clinical cure in participants treated with ibezapolstat, with no recurrence. Ibezapolstat was found to be safe, well tolerated, and was associated with the preservation of key health-promoting bacteria responsible for bile acid homoeostasis, a key component in preventing recurrent *C difficile* infection.

### Implications of all the available evidence

Ibezapolstat shows potential as a novel antibiotic treatment for *C difficile* infection with high rates of initial clinical cure and sustained clinical cure while minimally disturbing the protective gut microbiota. The findings of our study support further clinical development of ibezapolstat.
