# Supplementary material for: Efficacy, safety, pharmacokinetics, and associated microbiome changes of ibezapolstat compared with vancomycin in adults with Clostridioides difficile infection: a phase 2b, randomised, double-blind, active-controlled, multicentre study
Source: Lancet Microbe. Author manuscript; Available in PMC 2026 Mar 20. (PMC13003532; doi:10.1016/j.lanmic.2025.101126)

THE LANCET
Microbe

Supplementary appendix

This appendix formed part of the original submission and has been peer reviewed.
We post it as supplied by the authors.

Supplement to: Eubank TA, Jo J, Alam MJ, et al. Efficacy, safety, pharmacokinetics, and associated microbiome changes of ibezapolstat compared with vancomycin in adults with *Clostridioides difficile* infection: a phase 2b, randomised, double-blind, active-controlled, multicentre study. *Lancet Microbe* 2025. https://doi.org/10.1016/j.lanmic.2025.101126

Supplemental Material

**Page**	**Description**
1	Supplemental Figure 1. Study schema
2	Supplemental Figure 2. Time to resolution of diarrhea for CDI patients given ibezapolstat (IBZ) or vancomycin (VAN)
3	Supplemental Figure 3. Beta diversity results in patients given ibezapolstat ((IBZ) or vancomycin (VAN) at baseline (top panel) and during days 5-12 of therapy (bottom panel)
4 -	Study protocol

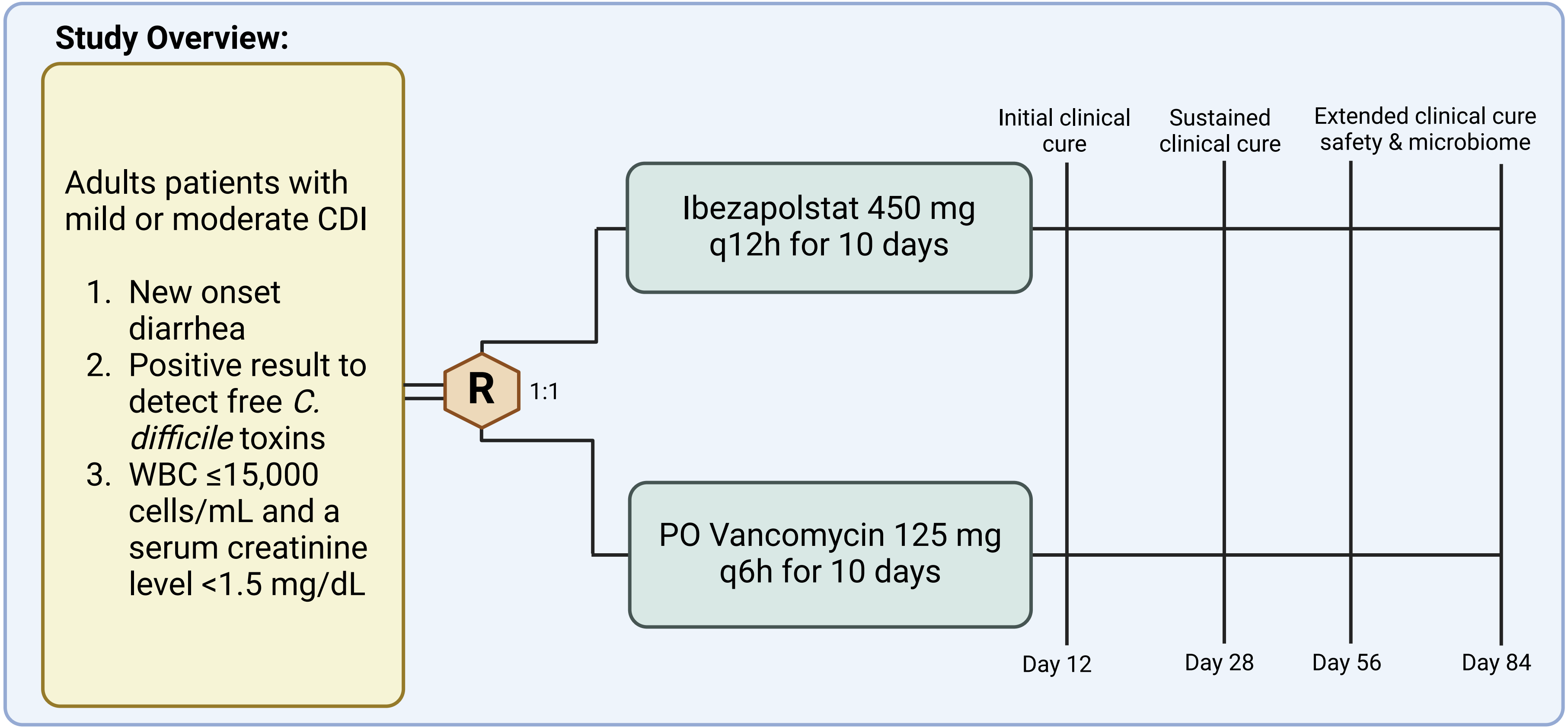

CDI, *Clostridioides difficile* infection; WBC, white blood cells; R, randomization; q12h, every 12 hours; q6h, every 6 hours

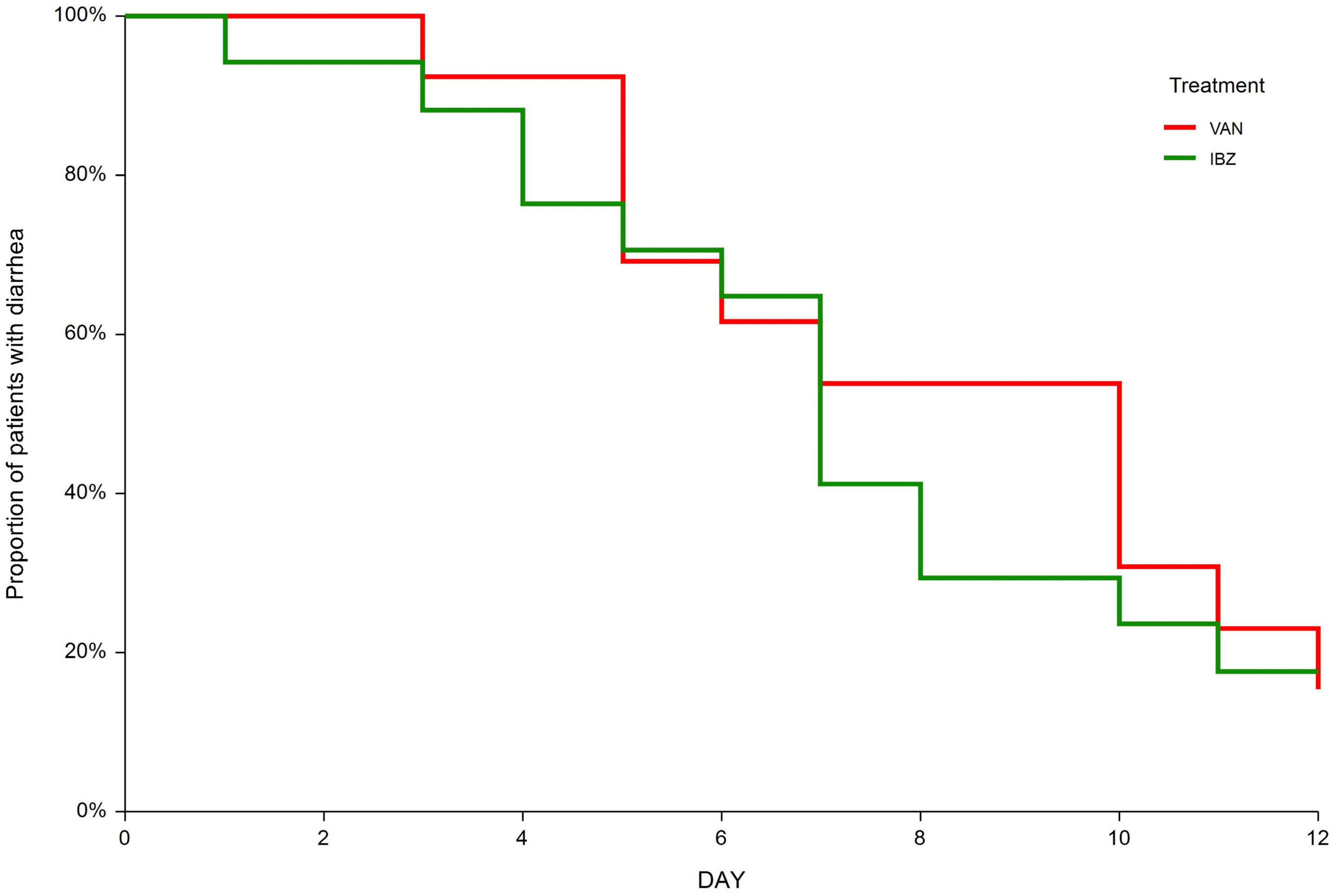

Number At Risk

Treatment = VAN	13	13	12	9	7	7	3
Treatment = IBZ	17	16	15	12	7	5	3

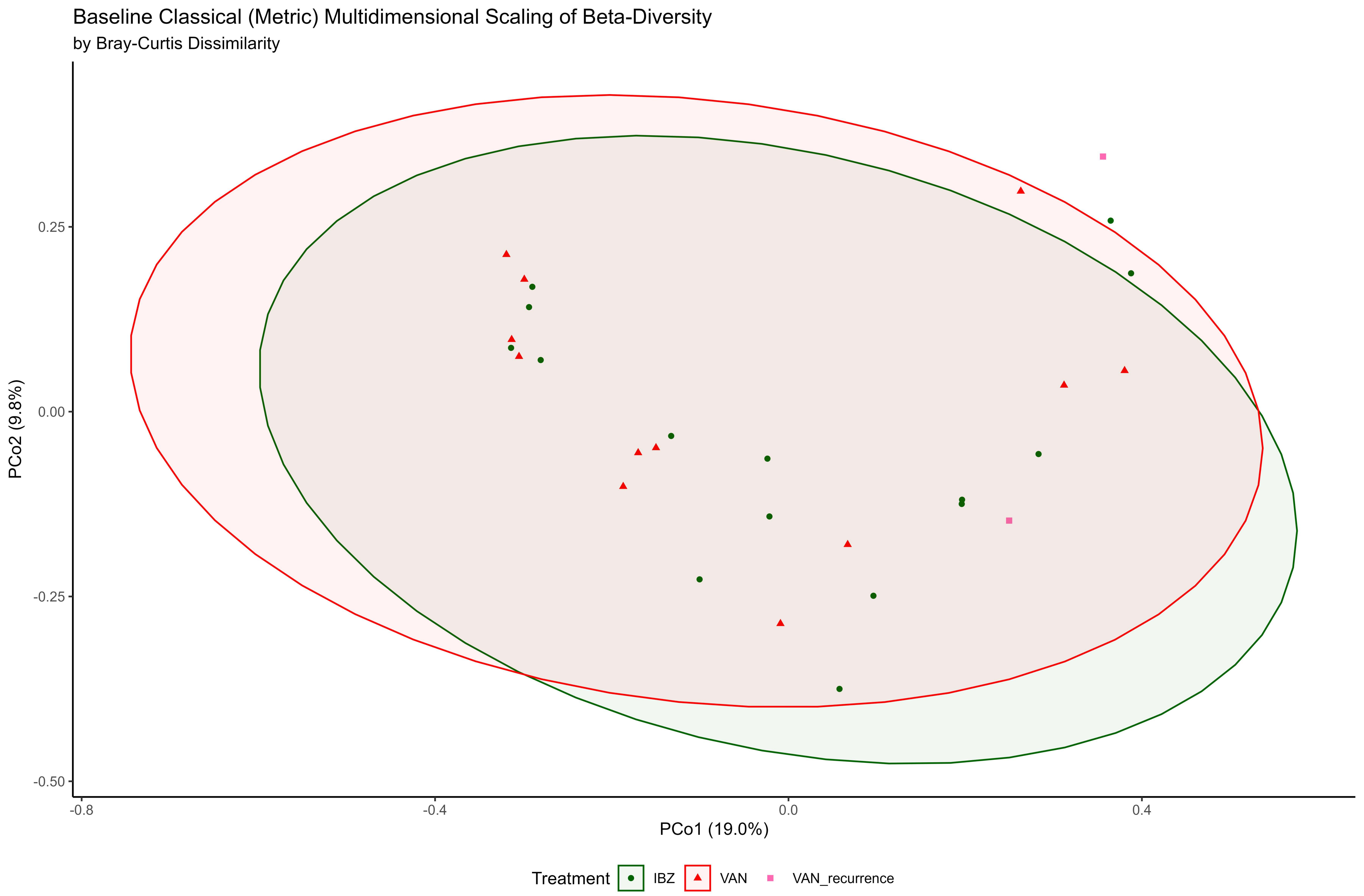
Baseline Classical (Metric) Multidimensional Scaling of Beta-Diversity
by Bray-Curtis Dissimilarity
PCo2 (9.8%)
0.25
0.00
-0.25
-0.50
-0.8
-0.4
0.0
0.4
PCo1 (19.0%)
Treatment
IBZ
VAN
VAN_recurrence

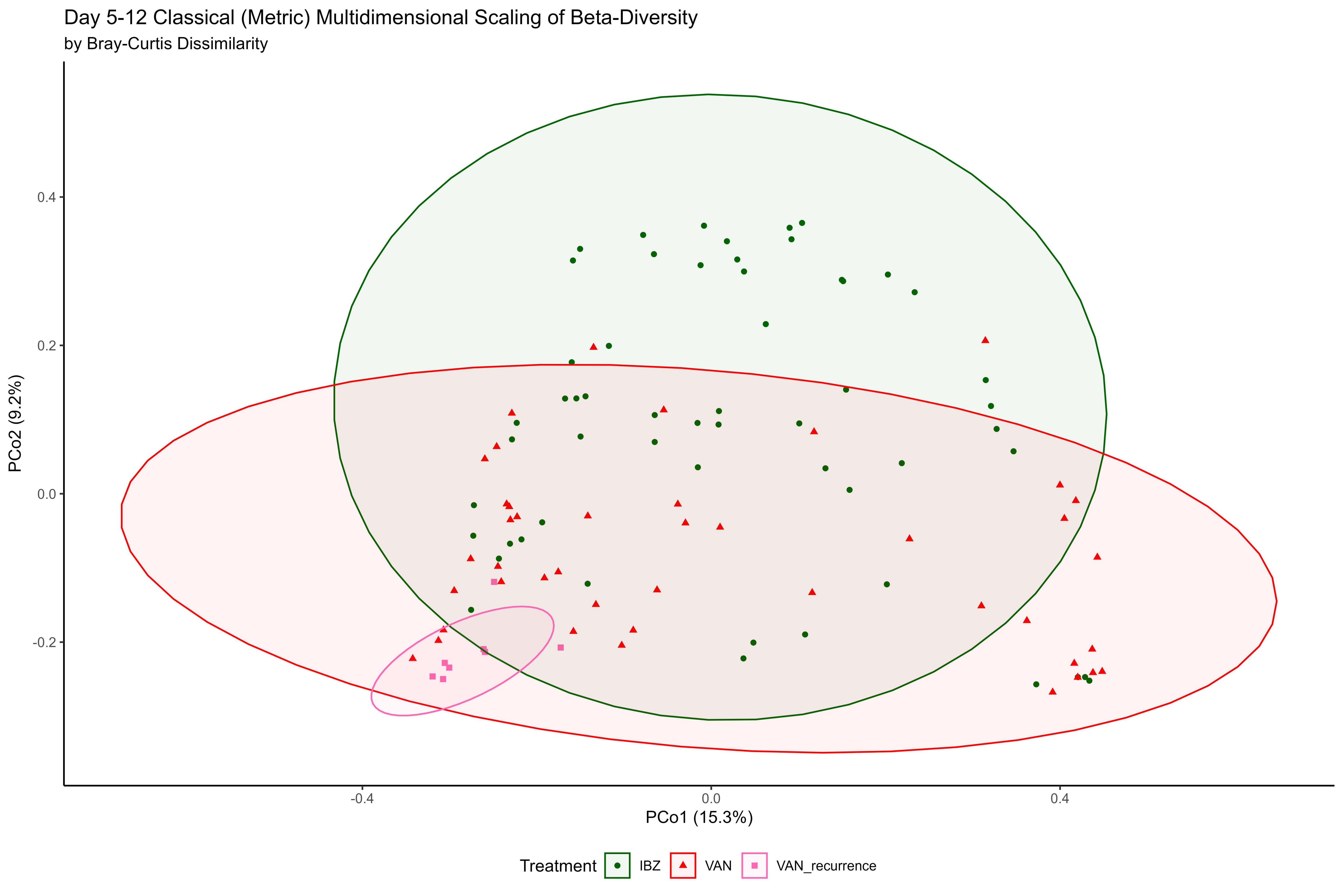
Day 5-12 Classical (Metric) Multidimensional Scaling of Beta-Diversity
by Bray-Curtis Dissimilarity
PCo2 (9.2%)
0.4
0.2
0.0
-0.2
-0.4
0.0
0.4
PCo1 (15.3%)
Treatment
IBZ
VAN
VAN_recurrence

1 CLINICAL STUDY PROTOCOL

Acurx Pharmaceuticals, Inc.

Protocol Title: Ibezapolstat (ACX-362E) for Oral Treatment of *Clostridioides difficile* Infection: A Phase 2A Open-Label Segment Followed by a Phase 2B Double-Blind Vancomycin-Controlled Segment

Protocol Number: ACX-362E-201

**Investigational New Drug Number:**	139236
**Name of Investigational Product**	Ibezapolstat (ACX-362E)
**Phase of Development:**	Phase 2
**Indication:**	*Clostridioides difficile* infection
**Sponsor:**	Robert J. DeLuccia<br>Executive Chairman<br>Acurx Pharmaceuticals, Inc.<br>259 Liberty Ave.<br>Staten Island, NY 10305<br>Tel: 914-949-3898<br>Email: rjdeluccia@acurxpharma.com
**NCT Number:**	04247542
**Protocol Version 1.0:**	10 Dec 2019
**Protocol Amendment 2.0**	09 Mar 2020
**Protocol Amendment 3.0**	18 Dec 2020
**Protocol Amendment 4.0**	01 Apr 2022
**Protocol Amendment 5.0**	30 Aug 2022
**Protocol Amendment 6.0**	03 Apr 2023

-CONFIDENTIAL-

This document and its contents are the property of and confidential to Acurx Pharmaceuticals, Inc.
Any unauthorized copying or use of this document is prohibited.

PROTOCOL APPROVAL SIGNATURES

Protocol Title: Ibezapolstat (ACX-362E) for Oral Treatment of *Clostridioides difficile* Infection: A Phase 2A Open-Label Segment Followed by a Phase 2B Double-Blind Vancomycin-Controlled Segment

Protocol Number: ACX-362E-201

This study will be conducted in compliance with the clinical study protocol (and amendments), International Council for Harmonisation (ICH) guidelines for current Good Clinical Practice (GCP) and applicable regulatory requirements.

Sponsor Signatory

Robert J. DeLuccia
Executive Chairman
Acurx Pharmaceuticals, Inc.
259 Liberty Ave.
Staten Island, NY 10305

Signature

05 April 2023

Date (DD-Mmm-YYYY)

Medical Monitors

Michael H. Silverman, MD, FACP
BioStrategics Consulting Ltd.
51 Intrepid Circle
Marblehead, MA 01945

Signature

05-April-2023

Date (DD-Mmm-YYYY)

Gino Girardi, MD
Sr Vice President
Syneos Health
100 Brandywine Boulevard, 2nd Floor
Newtown, PA 18940

Signature

03-April-2023

Date (DD-Mmm-YYYY)

This document is confidential.

INVESTIGATOR SIGNATURE PAGE

Protocol Title: Ibezapolstat (ACX-362E) for Oral Treatment of *Clostridioides difficile* Infection: A Phase 2A Open-Label Segment Followed by a Phase 2B Double-Blind Vancomycin-Controlled Segment

Protocol Number: ACX-362E-201

Confidentiality and Current Good Clinical Practice (GCP)/E6(R2) Compliance Statement

- I, the undersigned, have reviewed this protocol, including appendices, and I will conduct the study as described in compliance with this protocol, GCP, and relevant International Council for Harmonisation (ICH) guidelines.
- I am thoroughly familiar with the appropriate use of the study drug, as described in this protocol and any other information provided by Acurx Pharmaceuticals including, but not limited to, the current Investigator's Brochure.
- Once the protocol has been approved by the independent ethics committee (IEC)/institutional review board (IRB), I will not modify this protocol without obtaining prior approval of Acurx Pharmaceuticals and of the IEC/IRB. I will submit the protocol amendments and/or any informed consent form modifications to Acurx Pharmaceuticals and the IEC/IRB, and approval will be obtained before any amendments are implemented.
- I ensure that all persons or party assisting me with the study are adequately qualified and informed about the Acurx Pharmaceuticals study drug and of their delegated study-related duties and functions as described in the protocol.
- I ensure that source documents and trial records that include all pertinent observations on each of the site's trial subjects will be attributable, legible, contemporaneous, original, accurate, and complete.
- I understand that all information obtained during the conduct of the study with regard to the subjects' state of health will be regarded as confidential. No subjects' names will be disclosed. All subjects will be identified by assigned numbers on all case report forms, laboratory samples, or source documents forwarded to the Sponsor. Clinical information may be reviewed by the Sponsor or its agents or regulatory agencies. Agreement must be obtained from the subject before disclosure of subject information to a third party.
- Information developed in this clinical study may be disclosed by Acurx Pharmaceuticals to other clinical Investigators, regulatory agencies, or other health authority or government agencies as required.

Printed Name of Investigator ______________________________

Signature of Investigator ______________________________

Date ______________________________

Institution ______________________________

This document is confidential.

2 SYNOPSIS

**Title of Study:**	Ibezapolstat (ACX-362E) for Oral Treatment of *Clostridioides difficile* Infection: A Phase 2A Open-Label Segment Followed by a Phase 2B Double-Blind Vancomycin-Controlled Segment
**Protocol Number:**	ACX-362E-201
**Investigators/Study Sites:**	Up to 5 study centers in the United States (US) and Canada for Segment 2A and up to 30 study centers in United States and Canada for Segment 2B
**Phase of Development:**	Phase 2
**Objectives:**	**Primary Objectives:**<br>1. Assess *Clostridioides difficile* infection (CDI) clinical cure rates 2 days after the end of treatment (EOT) (Segment 2A/2B)<br>2. Evaluate the safety and tolerability of ibezapolstat (ACX-362E) administered every 12 hours for 10 days in the treatment of CDI (Segment 2A/2B)<br>**Secondary Objectives:**<br>1. Determine the systemic exposure (Segment 2A) and fecal concentrations (Segment 2A/2B) of ibezapolstat in subjects with CDI during the course of treatment.<br>2. Assess incidence of sustained clinical cure (SCC) at 28 ± 2 days after EOT (Segment 2A/2B)<br>**Exploratory Objectives:**<br>1. Compare the effects of ibezapolstat versus vancomycin on relative and quantitative changes to the fecal microbiome (Segment 2A/2B)<br>2. Assess times to resolution of diarrhea during the treatment period (Segment 2A/2B)<br>3. Assess times to hospital discharge during the treatment period (Segment 2A/2B)<br>4. Assess the impact of ibezapolstat treatment on subject reported quality of life and resource utilization (Segment 2B)<br>5. Assess incidence of extended clinical cure (ECC) at 56 ± 2 days and 84 ± 2 days after EOT in subjects participating in the extended follow-up period (Segment 2B)

This document is confidential.

**Study Endpoints:**	**Primary Endpoints:**<br>• Clinical cure at the test of cure (TOC) visit: defined as survival and the resolution of diarrhea in the 24-hour period immediately before EOT that is maintained for 48 hours post EOT without a requirement for additional CDI treatment. Diarrhea is defined as ≥ 3 unformed bowel movements (UBMs) in a 24-hour period; < 3 UBMs is considered as resolution of diarrhea. A UBM is defined as a Type 5, 6, or 7 bowel movement on the Bristol Stool Chart.<br>• Safety endpoints for all subjects: These include the nature, frequency, and severity of adverse events (AEs), including serious adverse events (SAEs); changes from baseline in findings on physical examination, vital sign measurements, safety laboratory tests (hematology, biochemistry, urinalysis); and electrocardiogram (ECG) findings.<br>**Secondary Endpoint:**<br>• SCC: this is defined as a clinical cure at the TOC visit (ie, at least 48 hours post EOT) and no recurrence of CDI within the 28 ± 2 days post EOT. Recurrence is defined as a new episode of diarrhea (≥ 3 UBMs in a 24-hour period) with a positive toxin result, using a Sponsor-approved *C. difficile* free toxin test and, in the opinion of the Investigator, requiring retreatment with an antibacterial agent for *C. difficile*.<br>**Pharmacokinetic Endpoint:**<br>• Systemic exposure of ibezapolstat will be determined by measuring plasma ibezapolstat concentrations at specified time points following dose administration; fecal concentrations of ibezapolstat will be measured at specified study visits.<br>**Exploratory Endpoints:**<br>• Microbial endpoints: Quantitative changes in relevant fecal bacterial communities and microbial diversity will be assessed for ibezapolstat-treated subjects in both segments, and in comparison to those treated with vancomycin in Segment 2B. Changes will be assessed during the treatment period and post EOT.<br>• Time to resolution of diarrhea, defined as the time from outset of treatment to the first formed bowel movement not followed within the next 24 hours by a UBM, will be recorded.<br>• Time to hospital discharge (for hospitalized subjects only) is defined as time from outset of treatment to day of discharge, and not followed by readmission for treatment of CDI before the TOC visit.<br>• Quality of life assessments including EuroQoL 5 Dimension 5 Level (EQ-5D-5L) and medical resource utilization data will be performed at designated time points during Segment 2B.<br>• ECC: this is defined as a clinical cure at the TOC visit (ie, at least 48 hours post EOT) and no recurrence of CDI within the 56 ± 2 days post EOT (ECC56) and 84 ± 2 days post EOT (ECC84). Recurrence is defined

This document is confidential.

	as a new episode of diarrhea (≥ 3 UBMs in a 24-hour period) with a positive toxin result, using a Sponsor-approved *C. difficile* free toxin test and, in the opinion of the Investigator, requiring retreatment with an antibacterial agent for *C. difficile*.
**Study Design:**	This Phase 2, multicenter, open-label single-arm segment followed by a double-blind, randomized, active-controlled segment is designed to evaluate ibezapolstat in the treatment of CDI. Segment 2A of this trial is an open-label cohort of up to 20 subjects in up to 5 study centers in the United States and Canada. In Segment 2A, up to 20 subjects with diarrhea caused by *C. difficile* will be treated with ibezapolstat 450 mg orally for 10 days. All subjects will be followed for recurrence for 28 ± 2 days. After 10 subjects have completed treatment and again after all subjects have completed treatment, a Trial Oversight Committee will assess the safety and tolerability, and, as data allow, efficacy, to make recommendation regarding whether the study should continue.<br><br>In Segment 2B, at least 72 additional subjects with CDI will be enrolled and randomized in a 1:1 ratio to either ibezapolstat 450 mg every 12 hours or vancomycin 125 mg orally every 6 hours for 10 days and followed for 28 ± 2 days for recurrence. Additionally, approximately 16 subjects recruited from selected sites will participate in an extended follow-up period consisting of observation visits at 56 days and 84 days after treatment to evaluate the long-term impact of ibezapolstat on the microbiome and on disease recurrence. The 2 treatments will be identical in appearance, dosing times, and number of capsules administered to maintain the blind.<br><br>Subjects in both segments will be evaluated for cure, safety, and tolerability. All subjects in both segments will have stool samples tested for microbiome profiles. Pharmacokinetic (PK) testing for systemic exposure will be performed on blood samples in Segment 2A and approximately 50% of subjects (ie, approximately 36 subjects) in Segment 2B. Stool samples will be tested for study drug concentration in Segment 2A and Segment 2B.
**Selection of Subjects:**	**Inclusion Criteria:**<br>1. Male or female 18 to 90 years of age, inclusive, at the time of Screening.<br>2. Capable of reading, understanding, and signing the written informed consent; able to adhere to all study procedures and attend all scheduled study visits.<br>3. Confirmed diagnosis of mild or moderate CDI as defined by the Infectious Diseases Society of America/Society for Healthcare Epidemiology of America guidelines. Subjects will be diagnosed with CDI based on clinical and laboratory findings:<br>a. The presence of diarrhea, defined as passage of ≥ 3 UBMs within 24 hours before dosing; an unformed stool is defined as a Type 5, 6, or 7 on the Bristol Stool Chart.<br>b. A stool test result positive for the presence of *C. difficile* free toxins using a test (approved by either the US Food and Drug Administration [FDA] or Health Canada) that detects toxins A/B

This document is confidential.

	(and is prospectively agreed with the Sponsor). The Sponsor will provide a toxin A/B test kit if the site does not have it as part of standard of care testing.<br>c. Mild or moderate CDI as defined as a white blood cell count of ≤ 15,000 cells/mL and a serum creatinine level < 1.5 mg/dL.<br>4. Agreement by subjects with reproductive potential to use two adequate methods of contraception during the study and for 4 weeks after the last study drug administration. A female subject is considered to be of reproductive potential following menarche and until she is in a postmenopausal state for 12 months or otherwise permanently sterile (for which acceptable methods include hysterectomy, bilateral salpingectomy, and bilateral oophorectomy). Female subjects must agree to the use of TWO reliable methods of contraception while receiving study drug and for 4 weeks after the last study drug administration if sexually active, which can include: condoms, spermicidal gel, diaphragm, hormonal or non-hormonal intrauterine device, surgical sterilization, oral contraceptive pill, and depot progesterone injections. If a male subject is sexually active, the subject and his partner will each use at least one of the listed contraceptive methods.<br>5. Negative serum pregnancy test at Screening and/or a negative urine pregnancy test on the day of admittance to the inpatient phase for all female subjects of childbearing potential.<br>6. Agreement in male subjects to not donate sperm starting at Screening and throughout the study period and for 90 days after the final study drug administration.<br>**Exclusion Criteria:**<br>1. Received more than 24 hours of dosing (> 4 doses) of oral vancomycin for the current episode of CDI before first dose of study drug.<br>2. Received more than 24 hours of dosing (> 2 doses) of oral fidaxomicin for the current episode of CDI before first dose of study drug.<br>3. Received more than 24 hours of dosing (> 3 doses) of oral/intravenous (IV) metronidazole for the current episode of CDI before first dose of study drug.<br>4. Received any other antibacterial therapy for the current CDI episode within 48 hours before the first dose of study drug.<br>5. Subjects considered treatment failures on prior antibiotics for their current episode of CDI will be excluded.<br>6. More than 3 episodes of CDI in the previous 12 months or more than 1 prior episode in the last 3 months, excluding the current episode.<br>7. Severe, complicated, or life-threatening fulminant CDI with evidence of hypotension (systolic blood pressure less than 90 mmHg), septic shock, peritoneal signs or ileus, or toxic megacolon.<br>8. Elevated liver transaminases (ALT, AST) greater than 2 times ULN.<br>9. Active inflammatory bowel disease (Crohn's disease, ulcerative colitis, Irritable Bowel Syndrome with chronic diarrhea).<br>10. Any other non-*C. difficile* diarrhea.

This document is confidential.

	11. Active gastroenteritis because of *Salmonella*, *Shigella*, *Escherichia coli* 0157H7, *Yersinia* or *Campylobacter*, a parasite, or virus within the past 2 weeks.<br>12. Had a known positive diagnostic test for other relevant gastrointestinal (GI) pathogens in the 2 weeks before study drug treatment and/or colonization/infection by ova or parasites.<br>13. Major GI surgery (ie, significant bowel resection) within 3 months of enrollment (does not include appendectomy or cholecystectomy).<br>14. Prior or current use of anti-*C. difficile* toxin antibodies.<br>15. Have received a vaccine against *C. difficile* or its toxins.<br>16. Anticipated that systemic antibacterial therapy for a non-CDI infection will be required for > 7 days after start of study therapy.<br>17. Actively taking anti-diarrheals, and unable to discontinue anti-diarrheal medication, or any medication with the potential to slow bowel movement (for opiates, a stable dose, including use as needed, is permitted).<br>18. Actively taking *Saccharomyces boulardii* and unwilling to discontinue during the study period.<br>19. Received a fecal transplant in the previous 3 months.<br>20. Received laxatives in the last 48 hours.<br>21. Unable or unwilling to stop taking oral probiotics for the duration of the study.<br>22. Received IV immunoglobulin within 3 months before study drug treatment.<br>23. Sepsis.<br>24. Have a known current history of significantly compromised immune system such as:<br>a. Subjects with a known history of human immunodeficiency virus infection and CD4 < 200 cells/mm^3 within 6 months of start of study therapy.<br>b. Severe neutropenia with neutrophil count < 500 cells/mL.<br>c. Concurrent intensive induction chemotherapy, radiotherapy, or biologic treatment for active malignancy.<br>25. Any specific condition that, in the judgment of the Investigator, precludes participation because it could affect subject safety.<br>26. Active participation in other clinical research studies using an investigational product or received investigational antibacterial agent within one month before Screening.<br>27. Pregnant or lactating women.<br>28. Known hypersensitivity to any ibezapolstat excipient.<br>29. Hypersensitivity or other contraindication to vancomycin.<br>30. Prior treatment with study drug in this trial.
**Planned Sample Size:**	Up to 20 subjects are planned for Segment 2A and at least 72 subjects are planned for Segment 2B.
**Investigational Therapy:**	Ibezapolstat 450 mg oral capsules taken with food every 12 hours for 10 days.
**Reference Therapy:**	Vancomycin 125 mg every 6 hours for 10 days.

**Treatment Duration:**	Subjects will receive study drug for 10 days.
**Efficacy**	Subjects will receive a diary and will be instructed to record in it each day of the study the date and time of dosing of study drug; date, time, and consistency of all bowel movements up to Day 12/TOC; and the date, time, and consistency of all UBMs from TOC to Day 38.<br><br>Sustained clinical cure will be determined in the 24-hour period immediately before EOT that is maintained for 48 hours post EOT.<br><br>SCC will be determined by following all subjects for 28 ± 2 days post treatment for recurrence of UBMs, although only subjects with clinical cure are eligible to be classified as SCC.
**Exploratory:**	The time to resolution of diarrhea will be determined by measuring the time from outset of treatment to the first formed bowel movement not followed within the next 24 hours by a UBM.<br><br>Time to hospital discharge will be determined by measuring time from the outset of treatment to the day of discharge that is not followed by a readmission for treatment of CDI before the TOC visit (for hospitalized subjects only).<br><br>Microbiome testing to determine the microbial content of stool samples will occur at specified time points during the study.<br><br>Quality of life assessments including EQ-5D-5L and medical resource utilization data will be performed at designated time points during Segment 2B.<br><br>ECC: this is defined as a clinical cure at the TOC visit (ie, at least 48 hours post EOT) and no recurrence of CDI within the 56 ± 2 days post EOT (ECC56) and 84 ± 2 days post EOT (ECC84). Recurrence is defined as a new episode of diarrhea (≥ 3 UBMs in a 24-hour period) with a positive toxin result, using a Sponsor-approved *C. difficile* free toxin test and, in the opinion of the Investigator, requiring retreatment with an antibacterial agent for *C. difficile*.
**Safety:**	Safety assessments include vital signs, physical examination, ECG recordings, collection of AEs, and clinical laboratory tests (hematology, biochemistry, and urinalysis).
**Pharmacokinetics:**	During Segment 2A and Segment 2B, stool samples will be collected to analyze ibezapolstat levels at designated time points. In Segment 2A and approximately 50% of subjects in Segment 2B, blood samples will be collected to analyze ibezapolstat levels at designated time points.
**Statistical Methods and Planned Analyses:**	**Study Populations**<br><br>The Enrolled population includes all subjects who sign an informed consent form.<br><br>The Intent-to-Treat (ITT) population includes all subjects who are enrolled and treated in Segment 2A or who are randomized in Segment 2B and will be used to analyze efficacy. In Segment 2B, subjects will be analyzed based on their randomized assignment. This population will be used for the primary analyses of efficacy.<br><br>The Per Protocol (PP) population will consist of subjects in the ITT population with no major protocol deviations. The determination of which subjects have major protocol violations will be made in a blinded manner prior to database lock. For analyses based on the PP population, subjects will be grouped

	according to the treatment to which they were randomized. This population will be used for a secondary analysis of the primary and secondary efficacy endpoints.<br><br>The ITT Extension population will include all randomized Segment 2B subjects who participate in the extended follow-up period. Subjects will be analyzed based on their randomized assignment.<br><br>The Safety population includes all subjects who receive at least 1 dose of study drug and will be used to analyze safety. Subjects will be analyzed based on the treatment actually received.<br><br>The PK population includes all subjects who take at least 1 dose of active study treatment (ibezapolstat) and have at least 1 quantifiable plasma concentration collected post dose without any important protocol deviations/violations or events that would affect the PK.<br><br>**Efficacy**<br><br>The primary efficacy endpoint is clinical cure at the TOC visit defined as survival and the resolution of diarrhea in the 24-hour period immediately before the EOT and maintained for 48 hours after EOT without a requirement for additional CDI treatment. For Segment 2A, this endpoint will be summarized using frequencies and percentages, together with a 95% exact (Clopper-Pearson) confidence interval for the true cure percentage. For Segment 2B, the null and alternative hypotheses for this endpoint are:<br><br>H_0: $\pi_{1,T} \leq \pi_{1,C} - \delta$ and H_1: $\pi_{1,T} > \pi_{1,C} - \delta$,<br><br>where $\pi_{1,T}$ and $\pi_{1,C}$ are the true proportions of subjects with clinical cure at the TOC visit for the ibezapolstat 450 mg and vancomycin 125 mg treatments, respectively, and δ is the non-inferiority margin which is here taken to be 0.25. This endpoint will be summarized by treatment group using frequencies and percentages, together with an exact (Clopper-Pearson) 95% confidence interval for the true cure percentage. A 95% confidence interval based on the normal approximation will also be calculated for the difference between the 2 population proportions. The null hypothesis will be tested using a one-sided normal approximation test of non-inferiority at the 0.03037 significance level (Pocock 1977) for an overall significance level of 0.05. In the event non-inferiority of ibezapolstat 450 mg is demonstrated, Fisher's exact test (two-sided) will be used to conduct a test for superiority at the 0.05 significance level.<br><br>A single interim analysis will be performed when the first 36 Segment 2B subjects in the ITT Population (one half of the planned sample size) have data for the primary efficacy endpoint. The null hypothesis (see Section 14.3.1) will be tested using a one-sided normal approximation test of non-inferiority at a one-sided α=0.03037 level of significance (Pocock 1977) for an overall significance level of 0.05. If the p-value is less than or equal to 0.03037 at the time of the interim analysis, then the study will be stopped early for success.<br><br>A secondary endpoint is SCC. For Segment 2A, this endpoint will be summarized using frequencies and percentages, together with an exact (Clopper-Pearson) 95% confidence interval for the true SCC percentage. For Segment 2B, this endpoint will be summarized by treatment group using frequencies and percentages, together with an exact (Clopper-Pearson) 95% confidence interval for the true SCC percentage. A 95% confidence interval based on the normal approximation will also be calculated for the difference between the 2 population proportions. The null hypothesis that the true

	proportions are equal for the 2 treatments will be tested using a two-sided Fisher's exact test at the 0.05 significance level.<br><br>**Safety**<br><br>Treatment-emergent AEs (TEAEs) will be coded using the most recent version of the Medical Dictionary for Regulatory Activities (MedDRA) and summarized. If a subject experiences more than 1 occurrence of the same AE, the occurrence with the greatest severity and the closest relationship with the study drug will be used in the summary tables.<br><br>The number and percentage of subjects with at least one TEAE, at least one treatment-related TEAE, at least one serious TEAE, and at least one TEAE leading to study discontinuation will be presented by treatment group. AEs that are definitely, probably, or possibly related, or for which the relationship is missing, will be considered related.<br><br>The number and percentage of subjects having a TEAE in each System Organ Class (SOC) and having each individual type of AE (Preferred Term) will be presented. TEAEs will also be summarized at the event level by SOC/Preferred Term and severity. This will be done for all TEAEs, all serious TEAEs, and all TEAEs leading to discontinuation from the study.<br><br>All AEs will be listed by subject, along with information regarding onset, duration, relationship and severity to study drug, action taken with study drug, treatment of event, and outcome.<br><br>Clinical laboratory data and vital signs data will be summarized by treatment group and time point using descriptive statistics, for both the actual values and the changes from baseline, as well as the numbers and percentages of subjects with values outside the limits of the normal range at each time point. Electrocardiogram data will be summarized by treatment group and time point using descriptive statistics for both the actual values and the changes from baseline.<br><br>Concomitant medications initiated during the study period will be summarized by treatment group using frequencies and percentages.<br><br>**Exploratory Analyses**<br><br>Quantitative changes in relevant fecal bacterial communities and microbial diversity will be determined at designated time points during the study. Quantitative changes in relevant fecal bacterial communities and microbial diversity will be assessed for ibezapolstat-treated subjects in both segments, and in comparison to those treated with vancomycin in Segment 2B. Changes will be assessed during the treatment period and post EOT.<br><br>For Segment 2A, time to resolution of diarrhea will be summarized using the Kaplan-Meier method, with estimates of the true resolution of diarrhea rate at Days 1, 5, 10, 12, and 40. The estimated median time to resolution of diarrhea, together with a 95% confidence interval, will also be presented. For Segment 2B, the analysis will be the same as for Segment 2A, except that the results will be presented by treatment group.<br><br>For Segment 2A, time to hospital discharge will be summarized using descriptive statistics. For Segment 2B, the analysis will be the same as for Segment A, except that the results will be presented by treatment group.<br><br>Medical resource utilization data will be summarized by treatment group and time point using descriptive statistics for continuous variables and frequencies and percentages for categorical variables.

This document is confidential.

	EQ-5D-5L scores for each of the 5 dimensions and the EQ-5D-5L visual analog scale score will be summarized by treatment group and time point using descriptive statistics.<br><br>ECC56 and ECC84 will be summarized by treatment group using frequencies and percentages, together with an exact (Clopper-Pearson) 95% confidence interval for the true percentage. A 95% confidence interval based on the normal approximation will also be calculated for the difference between the 2 population proportions.<br><br>These analyses will be performed for Segment 2B only.<br><br>Further details of the statistical analyses of the EQ-5D-5L and medical resource utilization will be described in the statistical analysis plan.<br><br>**Pharmacokinetic Analysis**<br><br>Systemic exposure of ibezapolstat will be determined by measuring plasma ibezapolstat concentrations at specified time points following dose administration. Fecal concentrations of ibezapolstat will be measured at specified study visits. Plasma and fecal ibezapolstat concentrations will be listed by study segment, treatment, study day and/or nominal time, and summarized using descriptive statistics.<br><br>Summary statistics will be generated for ibezapolstat plasma and fecal concentrations. Given sufficient plasma and fecal levels over the lower limit of quantitation (LLOQ), the data will be analyzed as follows: Median (and range) and mean (and standard deviation) ibezapolstat plasma concentrations at each nominal time point will be calculated. Concentrations reported as below the LLOQ will be set to zero for descriptive statistics.

This document is confidential.

3 TABLE OF CONTENTS

1 CLINICAL STUDY PROTOCOL 1
2 SYNOPSIS 4
3 TABLE OF CONTENTS 13
3.1 List of In-text Tables 17
3.2 List of In-Text Figures 17
4 LIST OF ABBREVIATIONS 18
5 INTRODUCTION 19
5.1 Background on Clostridioides Difficile 19
5.2 Background on Ibezapolstat 20
5.2.1 Nonclinical Studies 20
5.2.2 Clinical Studies 21
5.3 Clinical Risks/Benefits of Ibezapolstat 22
5.4 Study Rationale 22
5.4.1 Justification for Dose 24
6 STUDY OBJECTIVES AND ENDPOINTS 24
6.1 Study Objectives 24
6.1.1 Primary Objectives 24
6.1.2 Secondary Objectives 24
6.1.3 Exploratory Objectives 24
6.2 Study Endpoints 25
6.2.1 Primary Endpoint 25
6.2.2 Secondary Endpoints 25
6.2.2.1 Efficacy Endpoints 25
6.2.2.2 Pharmacokinetic Endpoint 25
6.2.3 Exploratory Endpoints 25
7 INVESTIGATIONAL PLAN 26
7.1 Description of Overall Study Design and Plan 26
7.2 Discussion of Study Design 28
7.3 End of Study 29
8 SELECTION OF STUDY POPULATION 29
8.1 Inclusion Criteria 29
8.2 Exclusion Criteria 30
8.3 Rescreening 31

This document is confidential.

8.4 Study Discontinuation, Removal, and Replacement of Subjects 31
8.4.1 Lost to Follow-Up 33
9 TREATMENTS 33
9.1 Ibezapolstat 33
9.1.1 Description 33
9.1.2 Manufacturing 33
9.1.3 How Supplied 33
9.1.4 Packaging and Labeling 33
9.1.5 Storage and Handling 33
9.1.6 Dietary and Lifestyle Restrictions 33
9.2 Vancomycin 34
9.3 Placebo 34
9.4 Dosage Schedule 34
9.5 Measures to Minimize Bias: Study Treatment Assignment and Blinding 35
9.5.1 Method of Study Treatment Assignment 35
9.5.2 Blinding 36
9.6 Dosage Modification 36
9.7 Treatment Accountability and Compliance 36
9.8 Prior and Concomitant Therapy 37
9.8.1 Prior Medications 37
9.8.2 Concomitant Medications 38
10 STUDY PROCEDURES 41
10.1 Informed Consent 45
10.2 Study Procedures 45
11 EFFICACY ASSESSMENTS 45
11.1 Subject Diary 45
11.2 Clinical Cure 46
11.3 Sustained Clinical Cure 47
11.4 Extended Clinical Cure 47
11.5 Microbial Assessments 47
11.6 Time to Resolution of Diarrhea 47
11.7 Time to Hospital Discharge 47
12 SAFETY AND TOLERABILITY ASSESSMENTS 47
12.1 Medical History 48
12.2 Vital Signs 48

12.3 Physical Examination 48
12.4 Electrocardiograms 49
12.5 Laboratory Assessments 49
12.6 Adverse Events 50
12.6.1 Serious Adverse Events 51
12.6.2 Serious Adverse Event Reporting 52
12.6.3 Suspected Unexpected Serious Adverse Reactions 52
12.6.4 Pregnancy 53
12.6.5 Overdose 54
12.7 EQ-5D-5L 54
12.8 Medical Resource Utilization Questions 54
13 PHARMACOKINETICS 54
13.1 Pharmacokinetic Sampling 54
13.1.1 Blood Samples 54
13.1.2 Stool Samples 55
13.2 Pharmacokinetic Bioanalytical Methodology 55
13.3 Calculation of Pharmacokinetic Variables 55
14 STATISTICAL ANALYSIS 55
14.1 Determination of Sample Size 56
14.2 Analysis Populations 56
14.2.1 Enrolled Population 56
14.2.2 Intent-to-Treat Population 57
14.2.3 Per Protocol Population 57
14.2.4 ITT Extension Population 57
14.2.5 Safety Population 57
14.2.6 Pharmacokinetic Population 57
14.3 Efficacy Analysis 57
14.3.1 Analysis of Primary Efficacy Endpoint 57
14.3.2 Analysis of Secondary Efficacy Endpoint: Sustained Clinical Cure 58
14.3.3 Analysis of Exploratory Endpoints 58
14.3.3.1 Microbial Assessments 58
14.3.3.2 Time to Resolution of Diarrhea 59
14.3.3.3 Time to Hospital Discharge 59
14.3.3.4 Quality of Life Assessments 59

This document is confidential.

14.3.3.5 ECC56 and ECC84 59
14.4 Safety Analysis 59
14.5 Pharmacokinetic Analysis 60
14.6 Interim Analysis 60
14.7 Trial Oversight Committee 61
15 STUDY MANAGEMENT 61
15.1 Approval and Consent 61
15.1.1 Regulatory Guidelines 61
15.1.2 Institutional Review Board/Independent Ethics Committee 61
15.1.3 Informed Consent 62
15.2 Data Handling 62
15.3 Source Documents 62
15.4 Record Retention 63
15.5 Monitoring 63
15.6 Quality Control and Quality Assurance 63
15.7 Protocol Amendment and Protocol Deviation 64
15.7.1 Protocol Amendment 64
15.7.2 Protocol Deviations 64
15.8 Ethical Considerations 64
15.9 Financing and Insurance 64
15.10 Publication Policy/Disclosure of Data 64
16 REFERENCES 66
17 APPENDICES 68
APPENDIX 1. CONTRACEPTION GUIDELINES 69
APPENDIX 2. BRISTOL STOOL CHART 72

3.1 List of In-text Tables

Table 1. Dose, Route, and Schedule of Study Medication for Segment 2B 35

Table 2. Daily Dosing for Segment 2B 35

Table 3. Potentially Confounding Medications...39

Table 4. Schedule of Assessments 42

Table 5. Laboratory Assessments..49

Table 6. Classification of Adverse Events by Intensity 50

Table 7. Classification of Adverse Events by Relationship to Study Drug 51

Table 8. Estimated Cure Rate and Associated 95% Confidence Intervals Based on the Normal Approximation 56

Table 9. Highly Effective Contraceptive Methods...70

3.2 List of In-Text Figures

Figure 1. Study Schematic...28

4 LIST OF ABBREVIATIONS

**Abbreviation**	**Definition**
AE	adverse event
CDI	*Clostridioides difficile* infection
CFR	Code of Federal Regulations
ECC	extended clinical cure
eCRF	electronic case report form
ECG	electrocardiogram
EDC	electronic data capture
EOT	end of treatment
FDA	Food and Drug Administration
GCP	Good Clinical Practice
GI	gastrointestinal
ICF	informed consent form
ICH	International Council for Harmonisation of Technical Requirements for Pharmaceuticals for Human Use
IEC	independent ethics committee
IRB	institutional review board
ITT	intent-to-treat
IWRS	interactive web response system
PI	Principal Investigator
PK	pharmacokinetic(s)
PP	Per protocol
SAE	serious adverse event
SAP	statistical analysis plan
SCC	sustained clinical cure
SUSAR	suspected unexpected serious adverse reaction
TEAE	treatment-emergent adverse event
TOC	test of cure
UBM	unformed bowel movements
US	United States
WOCBP	women of childbearing potential

This document is confidential.

5 INTRODUCTION

5.1 Background on Clostridioides Difficile

Clostridioides difficile (*C. difficile*) is an anaerobic, spore-forming, Gram-positive bacterium. It is the causative agent of *C. difficile* infection (CDI), an increasingly common, potentially life-threatening disease. *C. difficile* can live harmlessly in the colon, but in the presence of an antibiotic administered for another condition, overgrowth may occur, replacing normal bacterial flora and resulting in CDI and or *C. difficile*-associated disease (Nelson, 2017).

The epidemiology of *C. difficile* has changed with the emergence of the North American pulsed-field gel electrophoresis type 1 strain, and use of more sensitive assays (Lessa, 2015). CDI infection affects approximately 500,000 patients in the United States (US) each year, and is associated with 15,000 to 30,000 deaths (McDonald, 2018). In fact, 65,000 hospital acquired hospital-onset cases, 50,000 hospital acquired post-discharge cases, and 263,000 nursing home-onset cases, result in $4 billion in excess costs to the healthcare system annually (Clostridium Infections – Pipeline Review, 2012). Hospitalizations for CDI among nonpregnant adults has doubled from 2000 through 2010 and are projected to continue to increase in 2011 and 2012 (Lessa, 2015).

Although the discontinuation of predisposing antibiotic therapy and treatment with metronidazole, oral vancomycin, or the recently approved drug fidaxomicin (Dificid®), are effective in management of CDI, the development of new alternative therapeutic measures will be of great benefit. Not only is the incidence of hospitalizations and deaths associated with *C. difficile* rising (Lessa, 2015; Louie, 2012; Zilberberg, 2008), but some strains may become hypervirulent and increasingly resistant to cephalosporins and fluoroquinolones (Razavi, 2007).

Current treatment strategy includes discontinuation of predisposing antibiotic and oral administration of vancomycin or fidaxomicin. If access to vancomycin or fidaxomicin is limited, metronidazole may be used for an initial episode of nonsevere CDI only (McDonald, 2018).

While vancomycin continues to show good clinical efficacy for *C. difficile*, there is an increasing incidence of serious illness because of vancomycin-resistant enterococci in the United States, and use of oral vancomycin (as well as oral metronidazole) has been shown to promote overgrowth of this pathogen (Al-Nassir, 2008). Use of vancomycin to treat *C. difficile* is also associated with unacceptably high recurrence rates. Fidaxomicin (Dificid), although newly approved for treatment of CDI, is a treatment option if the risk of recurrence is high, but not for complicated CDI (Smits, 2016).

Several additional products are under development, and include small molecules, natural products, vaccines, and fecal transplants (Clostridium Infections – Pipeline Review, 2012; McDonald, 2018).

This document is confidential.

Ibezapolstat (ACX-362E) has activity in vitro and in vivo against *C. difficile* (Xu, 2011; Dvoskin, 2012). The crisis in antibiotic resistance of Gram-positive bacteria prompted the development of novel antibacterials through structure–activity relationship studies of replication-specific DNA polymerase IIIC. It is from among these compounds that antibacterial compounds against *C. difficile* with potent in vitro activity have been discovered. Among these is ibezapolstat, which also shows potent in vivo activity in the established Syrian golden hamster model of CDI (Kokkotou, 2008; Dvoskin, 2012).

Ibezapolstat is an inhibitor of the *C. difficile* DNA polymerase IIIC and is highly selective for *C. difficile* compared with other Gram-positive anaerobes and aerobic bacteria. Ibezapolstat, 2-(3,4-dichlorobenzyl)-7-(2-[morpholinyl]ethyl)guanine, is a small molecule, poorly bioavailable and active orally in the hamster model. In vitro, ibezapolstat inhibited numerous *C. difficile* strains, with a minimum inhibitory concentration required to inhibit the growth of 90% of organisms value of 4 µg/mL, and without inhibiting *Bifidobacteria* and *Lactobacillus spp*., important "good" intestinal anaerobic bacteria, or Gram-negative bacteria. These results suggest that development of ibezapolstat as an oral treatment for CDI in human patients will result in a novel, first-in-class drug to treat this emerging infectious disease.

Subjects in this study will be administered 450 mg ibezapolstat orally every 12 hours for 10 days. Ibezapolstat is administered orally because it has been shown that ibezapolstat has low oral availability with low systemic absorption, and also has a unique specificity for *C. difficile* that does not affect the normal good flora of the colon, an important advantage to prevent recurrence.

5.2 Background on Ibezapolstat

5.2.1 Nonclinical Studies

In 14-day multiple oral dose studies, doses of ibezapolstat were given orally at doses ranging from 60 to 600 mg/kg in dogs, and 100 to 2000 mg/kg/day in rats. Bioavailability appeared to be approximately 10% after comparing intravenous and oral dosing in both rats (approximately 8%) and dogs (approximately 11%). The apparent plasma clearance after single dosing appeared to be approximately 90 to 140 L/h in rats and 60 to 90 L/h in dogs after administration of oral doses ranging from 60 to 1000 mg/kg/day. No toxicity was seen in these studies so the no observed adverse effect level was > 600 mg/kg/day in dogs and > 2000 mg/kg/day in rats (data on file).

Ibezapolstat was excreted in urine and feces after oral administration in rats. Approximately 9% and 0.4% of the dose was excreted in feces and urine, respectively; considering the oral bioavailability is approximately 10%, and that ibezapolstat is metabolized to 11 different metabolites. Fecal concentrations after a 50 mg/kg oral dose in rats were 107 to 191 µg/g at 10 to 24 hours post dose (data on file).

For more information, refer to the Investigator's Brochure.

This document is confidential.

5.2.2 Clinical Studies

A Phase 1 study of ibezapolstat in healthy subjects (ACX-362E-101) has been completed. Study ACX-362E-101 was a randomized, double-blind, placebo-controlled, 3-part, single-ascending dose, multiple-ascending dose, food effect study in which ibezapolstat was administered in doses of 150 mg, 300 mg, 450 mg, 600 mg, and 900 mg. The primary objective of the study was to determine the safety of ibezapolstat in both single and multiple doses to healthy subjects. Secondary objectives of the study were to determine systemic and fecal pharmacokinetics (PK) of ibezapolstat after single and multiple doses and to compare fecal microbiome effects of ibezapolstat compared to those of vancomycin after multiple doses. The plasma and stool levels of ibezapolstat were also determined after a fed and fasted state. Part 1 (n = 6 ibezapolstat; n = 2 placebo) was a single-ascending dose, double-blind, randomized, placebo-controlled escalation study. Part 2 (n = 8 ibezapolstat) was a separate, single dose cohort at the dose determined in Part 1 and was a randomized, open-label, food effect, 2-period cross-over study in which subjects were assigned a single dose of ibezapolstat in either a high-fat, high-calorie, fed state followed by a fasted state, or in a fasted state followed by a high-fat, high-calorie fed state. Part 3 (n = 6 ibezapolstat; n = 2 placebo) was a multiple-dose, double-blind, randomized, placebo-controlled dose escalation study in which tolerability, plasma PK, fecal concentrations, and fecal microbiome effects of ibezapolstat were evaluated. In addition, a positive-control subgroup was randomly assigned to dosing with vancomycin and tolerability, fecal concentrations, and fecal microbiome effect were evaluated for these subjects.

Preliminary results from Study ACX-362E-101 showed a favorable safety profile. Adverse events (AEs) were well tolerated, similar to placebo, and transitory. AEs in subjects who received ibezapolstat included cough, cystitis-noninfective, dizziness, nasal congestion, twitching sensation, and headache that were considered by the Investigator to be unrelated to ibezapolstat and epigastric pain, headache (3 events), dyspepsia, nausea, prolonged PR interval, shortness of breath, and tachycardia that were considered by the Investigator to be possibly or probably related to ibezapolstat. All the events were mild in intensity except for 1 of moderate intensity (headache). No AE required a change in therapy or intervention. Ibezapolstat was shown to have ideal PK for a *C. difficile* antibiotic. Systemic concentrations of ibezapolstat after administration showed low systemic absorption. Fecal levels were several orders of magnitude higher than the microbial inhibitory concentration of *C. difficile* (4000 to 6000 µg/g stool). Study results demonstrated that after ibezapolstat administration, there is a favorable microbiome profile remaining in the gut with less disruption to normal microbiota compared to vancomycin, which was largely driven by decreased killing of *Bacteroidetes* and *Firmicutes* bacteria, which constitute > 80% of normal microbiota. These results support continuing the development of ibezapolstat for the treatment of CDI.

The food effect on ibezapolstat following a 300 mg single dose of ibezapolstat showed a peak plasma concentration of ibezapolstat of approximately 3 times greater in a fasting state

This document is confidential.

versus a fed state, although the ibezapolstat plasma concentration returned to zero sooner in the fasting state.

Comparison of multiple doses of 300 mg and 450 mg dosing of ibezapolstat showed plasma concentrations peaking at approximately 2 times greater with the 450 mg dose compared to the 300 mg dose. Multiple dosing showed higher peaks of ibezapolstat in stool samples in subjects who received 450 mg ibezapolstat compared to 300 mg ibezapolstat.

5.3 Clinical Risks/Benefits of Ibezapolstat

Based on the available nonclinical and clinical data to date, the conduct of the study, as specified in this protocol, is considered justifiable. The nonclinical data suggest an acceptable safety margin for the selected dose of ibezapolstat.

All subjects will receive an active treatment for their CDI in the study. Subjects who fail to respond to study treatment will be offered treatment for CDI according to the standard of care antibiotic at the Investigator's discretion.

This study will be performed in compliance with the protocol, International Council for Harmonisation Technical Requirements for Pharmaceuticals for Human Use (ICH) Good Clinical Practice (GCP) and applicable regulatory requirements. Aspects of the study concerned with the study drug will meet the requirements of Good Manufacturing Practice.

Subjects will be closely monitored for safety during the study. The study progresses in stages, only going to the next stage after the Trial Oversight Committee (Section 14.7) has fully assessed the safety and tolerability of the prior stage and recommends continuing the study.

The Sponsor will immediately notify the Principal Investigator (PI) if any additional safety or toxicology information becomes available during the study.

The collection of blood samples may be accompanied by mild bruising, but it is usually painless.

As this is the first administration of ibezapolstat to subjects with CDI, all effects cannot be reliably predicted. Facilities and staff for resuscitation and the treatment of other medical emergencies will be provided.

5.4 Study Rationale

The growing prevalence, epidemic nature, and health care burden of *C. difficile* diarrhea provides a strong rationale to discover and develop oral agents to treat the condition in addition to those in current use. To that end, purine derivatives with potent inhibition of the growth of *C. difficile* clinical isolates in culture have been identified. Based on the finding that these molecules, inhibitors of DNA polymerase IIIC (pol IIIC), are potent inhibitors of the growth of the anaerobic bacterium *C. difficile*, the gene for pol IIIC from this organism was cloned and expressed for testing. This showed that inhibition of this enzyme by these anti-*C. difficile* compounds is, as expected, the likely basis for their antibacterial effect. One

of these purine-based inhibitors of *C. difficile* pol IIIC, ibezapolstat, is highly active in vitro and in vivo, displaying excellent potential for further development as a clinical agent for the treatment of CDI (Torti, 2011).

New anti-CDI agents are needed to supplement those in current use for at least 2 significant reasons: firstly, ibezapolstat selectively inhibits pol IIIC, a target heretofore unexploited in *C. difficile* drug development. This property gives ibezapolstat strong potential for bypassing the resistance mechanism that may emerge in *C. difficile* during the prolonged administration of the agents in current use. Secondly, undesirable clinical issues frequently develop with prolonged use of some therapies such as vancomycin. Recurrence of *C. difficile* with vancomycin is a problem because vancomycin disrupts the normal balance of "good" bacteria in the gut, allowing "bad" bacteria to take over. Ibezapolstat, with its limited spectrum of activity, does not appear to disrupt the normal gut microbiome, thus having the potential to prevent the development and takeover of resistant strains needed for recurrence.

Data from Study ACX-362E-101 supports further rationale for advancing ibezapolstat into Phase 2 studies in the treatment of *C. difficile*. When administered to healthy subjects, ibezapolstat was well tolerated, with an AE profile similar to placebo. All AEs but 1 were mild, transient, and nonrecurrent despite continued dosing. No AE required a change in therapy or an intervention and there were no clinically important adverse changes in physical examinations, vital signs, laboratory testing results, or electrocardiograms (ECGs). Ibezapolstat was shown to be an ideal treatment for *C. difficile*; it has low systemic exposure after oral dosing and showed no accumulation following 10 days of repeat dosing, yet fecal concentrations rose rapidly and were sustained at 2 to 3 orders of magnitude above the minimum inhibitory concentration for *C. difficile*. Furthermore, after treatment with ibezapolstat, the fecal microbiome remained favorable, with less disruption to microbiota compared to vancomycin. Ibezapolstat treatment resulted in decreased killing of *Bacteroidetes* and *Firmicutes*, which generally comprise 80% to 90% of the healthy fecal microbiome. The 450 mg dose of ibezapolstat proved to result in higher fecal concentrations of ibezapolstat over the 300 mg dose, while also having an acceptable safety profile, and thus was chosen as the dose moving forward. Treatment with oral vancomycin is the standard of care for patients with CDI. Vancomycin treatment will be incorporated as a positive control into this study to allow accurate and sophisticated comparison of the respective effects of ibezapolstat and vancomycin on the fecal microbiome. Such data will provide valuable information regarding potential advantages of ibezapolstat over the current standard of care, such as preservation of "good" elements of the microbiome or quicker recovery to pretreatment profiles. Furthermore, comparative microbiome profiling may provide information on the benefits of ibezapolstat to maintain normal intestinal flora, which has the potential to improve the efficiency of future ibezapolstat efficacy studies.

This document is confidential.

5.4.1 Justification for Dose

The ibezapolstat dose chosen for this study is 450 mg. The results from Study ACX-362E-101 showed that after administration of ibezapolstat 450 mg every 12 hours, stool concentrations of ibezapolstat peak at higher concentrations compared to the 300 mg dose given every 12 hours and are consistently 2 to 3 orders of magnitude above the *C. difficile* minimum inhibitory concentration required to inhibit the growth of 90% of organisms. The 450 mg dose had an acceptable safety and tolerability profile and thus was selected to ensure presumed therapeutic levels of ibezapolstat in the gastrointestinal (GI) tract of subjects with CDI, while minimally impacting normal gut microbiome composition.

Vancomycin is a registered product and is commercially available. The usual adult total daily dose is 500 mg to 2 g administered orally in 3 or 4 divided doses for 7 to 10 days. For this study, 125 mg vancomycin will be administered approximately every 6 hours for 10 days, per current treatment guidelines for CDI (McDonald, 2018).

The 10-day duration of treatment was selected because: (a) this is the recommended duration of therapy for all current standard of care oral CDI drugs, according to the most recent Infectious Diseases Society of America and Society for Healthcare Epidemiology of America Guidelines (McDonald, 2018); (b) by the same token, it is the standard regimen of oral vancomycin, which is being used as the active control agent in the Phase 2B segment of this trial; (c) Phase 1 data have established the safety of ibezapolstat over 10 days of oral dosing; and (d) no human data exist to indicate that a shorter course of therapy with ibezapolstat would achieve acceptable efficacy for the treatment of CDI.

6 STUDY OBJECTIVES AND ENDPOINTS

6.1 Study Objectives

6.1.1 Primary Objectives

1. Access CDI clinical cure rates 2 days after the end of treatment (EOT) (Segment 2A/2B)
2. Evaluate the safety and tolerability of ibezapolstat administered every 12 hours for 10 days in the treatment of CDI (Segment 2A/2B)

6.1.2 Secondary Objectives

1. Determine the systemic exposure (Segment 2A) and fecal concentrations (Segment 2A/2B) of ibezapolstat in subjects with CDI during the course of treatment
2. Assess incidence of sustained clinical cure (SCC) at 28 ± 2 days after EOT (Segment 2A/2B)

6.1.3 Exploratory Objectives

1. Compare the effects of ibezapolstat versus vancomycin on relative and quantitative changes to the fecal microbiome (Segment 2A/2B)
2. Assess times to resolution of diarrhea during the treatment period (Segment 2A/2B)

This document is confidential.

3. Assess times to hospital discharge during the treatment period (Segment 2A/2B)
4. Assess the impact of ibezapolstat treatment on subject reported quality of life and resource utilization (Segment 2B)
5. Assess the incidence of extended clinical cure (ECC) at 56 ± 2 days and 84 ± 2 days after EOT in subjects participating in the extended follow-up period (Segment 2B)

6.2 Study Endpoints

6.2.1 Primary Endpoint

The primary endpoints of this study are:

- Clinical cure at the test of cure (TOC) visit: defined as survival and the resolution of diarrhea in the 24-hour period immediately before EOT that is maintained for 48 hours post EOT without a requirement for additional CDI treatment. Diarrhea is defined as ≥ 3 unformed bowel movements (UBMs) in a 24-hour period; < 3 UBMs is considered as resolution of diarrhea. A UBM is defined as a Type 5, 6, or 7 bowel movement on the Bristol Stool Chart (Appendix 2).
- Safety endpoints for all subjects: These include the nature, frequency, and severity of AEs, including serious adverse events (SAEs); changes from baseline in findings on physical examination, vital sign measurements, safety laboratory tests (hematology, biochemistry, urinalysis); and ECG findings.

6.2.2 Secondary Endpoints

6.2.2.1 Efficacy Endpoints

The secondary efficacy endpoint of this study is:

- SCC: this is defined as a clinical cure at the TOC visit (ie, at least 48 hours post EOT) and no recurrence of CDI within the 28 ± 2 days post EOT. Recurrence is defined as a new episode of diarrhea (≥ 3 UBMs in a 24-hour period) with a positive toxin result, using a Sponsor-approved *C. difficile* free toxin test and, in the opinion of the Investigator, requiring retreatment with an antibacterial agent for *C. difficile*.

6.2.2.2 Pharmacokinetic Endpoint

The secondary PK endpoint is as follows:

- Systemic exposure of ibezapolstat will be determined by measuring plasma ibezapolstat concentrations at specified time points following dose administration; fecal concentrations of ibezapolstat will be measured at specified study visits.

6.2.3 Exploratory Endpoints

The exploratory endpoints of this study are as follows:

- Microbial endpoints: Quantitative changes in relevant fecal bacterial communities and microbial diversity will be assessed for ibezapolstat-treated subjects in both segments, and in comparison to those treated with vancomycin in Segment 2B.

This document is confidential.

Changes will be assessed during the treatment period and post EOT. Details of the final microbiome analysis will be described in the statistical analysis plan (SAP) or relevant laboratory manual.

- Time to resolution of diarrhea, defined as the time from outset of treatment to the first formed bowel movement not followed within the next 24 hours by a UBM, will be recorded.
- Time to hospital discharge (for hospitalized subjects only) is defined as time from outset of treatment to day of discharge, and not followed by readmission for treatment of CDI before the TOC visit.
- Quality of life assessments including EuroQol 5 Dimension 5 Level (EQ-5D-5L) and medical resource utilization data will be performed at designated time points during Segment 2B.
- ECC: this is defined as a clinical cure at the TOC visit (ie, at least 48 hours post EOT) and no recurrence of CDI within the 56 ± 2 days post EOT (ECC56) and 84 ± 2 days post EOT (ECC84). Recurrence is defined as a new episode of diarrhea (≥ 3 UBMs in a 24-hour period) with a positive toxin result, using a Sponsor-approved *C. difficile* free toxin test and, in the opinion of the Investigator, requiring retreatment with an antibacterial agent for *C. difficile*.

7 INVESTIGATIONAL PLAN

7.1 Description of Overall Study Design and Plan

This Phase 2, multicenter, combined segment open-label single-arm segment followed by a double-blind, randomized, active-controlled clinical segment is designed to evaluate ibezapolstat in the treatment of CDI.

Segment 2A of this trial is an open-label cohort of up to 20 subjects in up to 5 study centers in the United States and Canada. If safety, tolerability, and efficacy are considered acceptable by the Trial Oversight Committee, Segment 2A will be followed by a double-blind, randomized, active-controlled trial with an additional cohort of at least 72 subjects.

In Segment 2A, up to 20 subjects with diarrhea caused by *C. difficile* will be enrolled and treated with ibezapolstat 450 mg orally every 12 hours for 10 days. All subjects will be followed for recurrence for 28 ± 2 days, regardless of response to ibezapolstat. After up to 10 subjects have been enrolled in the study and completed the full course of treatment in Segment 2A, there will be an assessment of safety, tolerability, and, as data allow, efficacy, by the Trial Oversight Committee, which will make a recommendation regarding continued enrollment of Segment 2A. The Trial Oversight Committee will consist of both the Sponsor's Medical Monitor (Chair), the Syneos Health Medical Monitor, and up to 2 PIs who enrolled subjects in Segment 2A.

This document is confidential.

Another assessment of safety, tolerability, and efficacy will be made by the Trial Oversight Committee after all subjects have been enrolled in Segment 2A and followed through at least the EOT visit.

In Segment 2B, at least 72 additional subjects with CDI will be enrolled and randomly assigned in a 1:1 ratio to either ibezapolstat 450 mg orally every 12 hours with food or to vancomycin 125 mg orally every 6 hours in up to 30 centers in the United States and Canada. Treatment will be administered for 10 days, and subjects will be followed for 28 ± 2 days for recurrence. Blinding in Segment 2B will be maintained by over-encapsulation of both drugs and dosing every 6 hours in all subjects. Subjects randomized to ibezapolstat will receive 2 doses per day of ibezapolstat and 2 doses per day of placebo. Additionally, subjects recruited from selected sites will participate in an extended follow-up period consisting of observation visits at 56 and 84 days after treatment to evaluate the long-term impact of ibezapolstat on the microbiome and on disease recurrence. At these selected sites, consecutive subjects will be offered the opportunity to participate in the extended follow-up period until approximately 16 subjects are recruited.

For all subjects, the diagnosis of CDI will be based on unexplained and new onset of diarrhea (≥ 3 UBMs in a 24-hour period) and a positive result using a US Food and Drug Administration (FDA)-approved test to detect free *C. difficile* toxins A and B in stools. Baseline safety evaluations will consist of physical examination, vital signs, routine laboratory (hematology, biochemistry, and urinalysis) testing, and ECG.

Subjects will be evaluated for treatment response and safety (AEs, physical examination, vital signs, laboratory tests, and ECG) at designated time points according to Table 4. Clinical cure of CDI will be defined as survival and the resolution of diarrhea in the 24-hour period immediately before EOT that is maintained for 48 hours post EOT without a requirement for additional CDI treatment. All subjects, regardless of outcome after 10 days of treatment, will be followed for recurrence until Day 38; recurrence is defined as a new episode of diarrhea (≥ 3 UBMs in a 24-hour period) with a positive free toxin test result (using a Sponsor-approved diagnostic test) that in the opinion of the Investigator requires retreatment with an antibacterial for *C. difficile*.

Stool samples for microbiome testing in Segment A and Segment B will be collected at the time points outlined in Table 4, frozen, and sent to the central laboratory for analysis. An additional sample will be collected in the event of any suspected recurrence post TOC, regardless of the outcome from the 10 days of treatment. Samples will also be collected in the event of any suspected recurrence at 56 and 84 days after EOT for those subjects participating in the extended follow-up period. Evaluation of changes to the microbiome at the TOC visit as well as overgrowth by vancomycin-resistant *Enterococcus* and multi-drug resistant organisms will be performed using Next Gen metagenomics sequencing and quantitative polymerase chain reaction.

This document is confidential.

For PK analysis in Segment 2A and 2B, fecal concentrations of ibezapolstat will be determined for the stool samples collected at time points outlined in Table 4. Blood samples to measure plasma levels of ibezapolstat in Segment 2A and approximately 50% of subjects (ie, approximately 36 subjects) in Segment 2B will be drawn 2 hours and 4 hours after the morning dose at time points specified in Table 4.

Figure 1 presents the study design.

Figure 1. Study Schematic

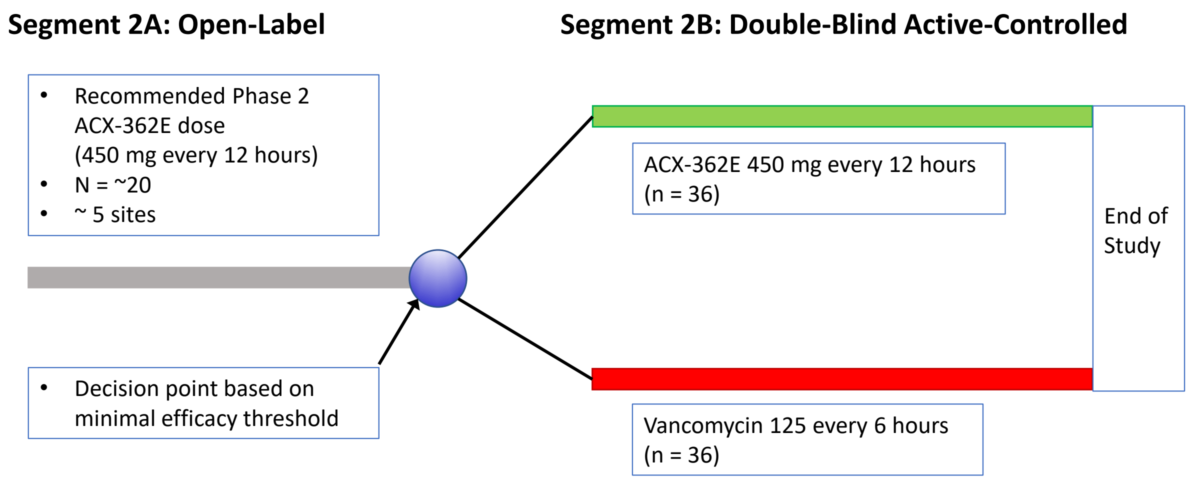

7.2 Discussion of Study Design

Ibezapolstat is under clinical development for the treatment of CDI.

A 10-day treatment period was selected to support the primary endpoint of initial cure rate and the 28 ± 2 days post EOT is considered the most appropriate measure of efficacy for sustained cure for comparison to vancomycin, since it captures both cure of the initial infection and any onset of CDI recurrence.

The open-label Segment 2A will allow an initial evaluation of the safety, tolerability, and efficacy of ibezapolstat in a limited number of subjects diagnosed with CDI, since there has been only limited clinical exposure in healthy subjects to date and no clinical exposure in subjects diagnosed with CDI. The randomized, double-blind Segment 2B allows an evaluation of the safety, tolerability, and efficacy of ibezapolstat in a blinded comparison to vancomycin, the current standard of care for CDI. The extended follow-up period allows an evaluation of the long-term impact of ibezapolstat on the microbiome and on the incidence of recurrent CDI.

To ensure the randomized population has a true CDI, the diagnosis of CDI is confirmed by the presence of free toxins in stool, which is the most rigorous sign of true infection in accordance with treatment guidelines.

Vancomycin 125 mg every 6 hours over 10 days is a standard therapy option for CDI.

This document is confidential.

7.3 End of Study

A subject will have fulfilled the requirements for study completion if/when the subject has completed all study periods, including the Follow-Up End of Study Day 38 visit, and for those Segment 2B subjects participating in the extended follow-up period, observation visits Day 66 (56 days post EOT) and Day 94 (84 days post EOT), as indicated in the Schedule of Assessments (Table 4).

The end of the study will be the last subject's last visit or the last subject's scheduled visit/assessment as indicated in the Schedule of Assessments (Table 4).

8 SELECTION OF STUDY POPULATION

Up to 20 evaluable subjects with mild or moderate CDI are planned to be enrolled in Segment 2A and at least 72 subjects with mild or moderate CDI are planned to be randomized for Segment 2B. Subjects must meet all of the inclusion criteria and none of the exclusion criteria to be eligible for the study.

8.1 Inclusion Criteria

Subjects must meet all of the following criteria to be included in the study:

1. Male or female 18 to 90 years of age, inclusive, at the time of Screening.
2. Capable of reading, understanding, and signing the written informed consent; able to adhere to all study procedures and attend all scheduled study visits.
3. Confirmed diagnosis of mild or moderate CDI as defined by the Infectious Diseases Society of America/Society for Healthcare Epidemiology of America guidelines (McDonald et al. 2018). Subjects will be diagnosed with CDI based on clinical and laboratory findings:
   a. The presence of diarrhea, defined as passage of ≥ 3 UBMs within 24 hours before dosing; an unformed stool is defined as a Type 5, 6, or 7 on the Bristol Stool Chart (Appendix 2).
   b. A stool test result positive for the presence of *C. difficile* free toxins using a test (approved by either the US FDA or Health Canada) that detects toxins A/B (and is prospectively agreed with the Sponsor). The Sponsor will provide a toxin A/B test kit if the site does not have it as part of standard of care testing.
   c. Mild or moderate CDI as defined as a white blood cell count of ≤ 15,000 cells/mL and a serum creatinine level < 1.5 mg/dL.
4. Agreement by subjects with reproductive potential to use two adequate methods of contraception during the study and for 4 weeks after the last study drug administration. A female subject is considered to be of reproductive potential following menarche and until she is in a postmenopausal state for 12 months or otherwise permanently sterile (for which acceptable methods include hysterectomy, bilateral salpingectomy, and bilateral oophorectomy). Female subjects must agree to the use of TWO reliable methods of contraception while receiving study drug and for 4 weeks after the last study drug

administration if sexually active, which can include: condoms, spermicidal gel, diaphragm, hormonal or non-hormonal intrauterine device, surgical sterilization, oral contraceptive pill, and depot progesterone injections. If a male subject is sexually active, the subject and his partner will each use at least one of the listed contraceptive methods. See Appendix 1 for contraception guidelines for the study.

5. Negative serum pregnancy test at Screening and/or a negative urine pregnancy test on the day of admittance to the inpatient phase for all female subjects of childbearing potential.
6. Agreement in male subjects to not donate sperm starting at Screening and throughout the study period and for 90 days after the final study drug administration.

8.2 Exclusion Criteria

Subjects meeting any of the following criteria at Screening or baseline are ineligible to participate in this study:

1. Received more than 24 hours of dosing (> 4 doses) of oral vancomycin for the current episode of CDI before first dose of study drug.
2. Received more than 24 hours of dosing (> 2 doses) of oral fidaxomicin for the current episode of CDI before first dose of study drug.
3. Received more than 24 hours of dosing (> 3 doses) of oral/intravenous (IV) metronidazole for the current episode of CDI before first dose of study drug.
4. Received any other antibacterial therapy for the current CDI episode within 48 hours before the first dose of study drug.
5. Subjects considered treatment failures on prior antibiotics for their current episode of CDI will be excluded.
6. More than 3 episodes of CDI in the previous 12 months or more than 1 prior episode in the last 3 months, excluding the current episode.
7. Severe, complicated, or life-threatening fulminant CDI with evidence of hypotension (systolic blood pressure less than 90 mmHg), septic shock, peritoneal signs or ileus, or toxic megacolon.
8. Elevated liver transaminases (ALT, AST) greater than 2 times ULN.
9. Active inflammatory bowel disease (Crohn's disease, ulcerative colitis, Irritable Bowel Syndrome with chronic diarrhea).
10. Any other non-*C. difficile* diarrhea.
11. Active gastroenteritis because of *Salmonella*, *Shigella*, *Escherichia coli* 0157H7, *Yersinia* or *Campylobacter*, a parasite, or virus within the past 2 weeks.
12. Had a known positive diagnostic test for other relevant GI pathogens in the 2 weeks before study drug treatment and/or colonization/infection by ova or parasites.
13. Major GI surgery (ie, significant bowel resection) within 3 months of enrollment (does not include appendectomy or cholecystectomy).
14. Prior or current use of anti-*C. difficile* toxin antibodies.
15. Have received a vaccine against *C. difficile* or its toxins.

This document is confidential.

16. Anticipated that systemic antibacterial therapy for a non-CDI infection will be required for > 7 days after start of study therapy.
17. Actively taking anti-diarrheals, and unable to discontinue anti-diarrheal medication, or any medication with the potential to slow bowel movement (for opiates, a stable dose, including use as needed, is permitted).
18. Actively taking *Saccharomyces boulardii* and unwilling to discontinue during the study period.
19. Received a fecal transplant in the previous 3 months.
20. Received laxatives in the last 48 hours.
21. Unable or unwilling to stop taking oral probiotics for the duration of the study.
22. Received IV immunoglobulin within 3 months before study drug treatment.
23. Sepsis.
24. Have a known current history of significantly compromised immune system such as:
    a. Subjects with a known history of human immunodeficiency virus infection and CD4 < 200 cells/mm^3 within 6 months of start of study therapy.
    b. Severe neutropenia with neutrophil count < 500 cells/mL.
    c. Concurrent intensive induction chemotherapy, radiotherapy, or biologic treatment for active malignancy.
25. Any specific condition that, in the judgment of the Investigator, precludes participation because it could affect subject safety.
26. Active participation in other clinical research studies using an investigational product or received investigational antibacterial agent within one month before Screening.
27. Pregnant or lactating women.
28. Known hypersensitivity to any ibezapolstat excipient.
29. Hypersensitivity or other contraindication to vancomycin.
30. Prior treatment with study drug in this trial.

8.3 Rescreening

Subjects who sign the informed consent form (ICF) to participate in the study but who do not subsequently meet all the requirements as outlined in the inclusion and exclusion criteria and therefore do not enroll (screen failures) may be rescreened. Such individuals may be allowed to rescreen up to 2 times. Subjects with a negative toxin text will not be rescreened, unless the subject is experiencing a new, separate diarrheal episode.

8.4 Study Discontinuation, Removal, and Replacement of Subjects

Discontinuation of study treatment for a subject occurs when study treatment is stopped earlier than the protocol planned duration. Discontinuation of study treatment can be decided by either the subject or the Investigator.

If a subject discontinues study treatment and is withdrawn from the study for any reason, the study site must immediately notify the Medical Monitor. The date and the reason for study discontinuation must be recorded on the electronic case report form (eCRF). Subjects who

complete or discontinue early from the study will be asked to return to the study site within 10 days of the last administration of study drug to complete End of Study assessments as indicated in the Schedule of Assessments (Table 4).

In the event that a subject discontinues prematurely from the study because of a treatment-emergent AE (TEAE) or serious TEAE, the TEAE or serious TEAE will be followed up until it resolves (returns to normal or baseline values) or stabilizes, or until it is judged by the Investigator to no longer be clinically significant.

Once a subject is withdrawn from the study, the subject may not reenter the study.

A subject may voluntarily withdraw or be withdrawn from the study at any time for reasons including, but not limited to, the following:

- subject withdrawal of consent: at any time, a subject's participation in the study may be terminated at his/her request or on the basis of the Investigator's clinical judgment for safety, behavioral, compliance or administrative reasons
- unacceptable toxicity or AE/SAE
- intercurrent illness: a condition, injury, or disease unrelated to the primary diagnosis that became apparent during treatment and necessitated the subject's termination from the study
- general or specific changes in the subject's condition that renders him/her ineligible for further treatment according to the inclusion/exclusion criteria
- subject fails to adhere to the protocol requirements (eg, drug noncompliance, failure to return for defined number of visits)
- lost to follow-up: the subject stopped coming for visits, and study personnel were unable to contact the subject
- pregnancy, as indicated in Section 12.6.4.

Additionally, the Sponsor may stop the study at any time for safety, regulatory, legal, or other reasons aligned with GCP studies. This study may be terminated at the discretion of the Sponsor or any regulatory agency. An Investigator may elect to discontinue or stop the study at his or her study site for any reason, including safety or low enrollment.

Subjects who discontinue the study will not be replaced. Subjects who discontinue the study will be offered treatment for CDI according to the standard of care antibiotic, at the Investigator's discretion.

If the subject withdraws consent for disclosure of future information, the Sponsor may retain and continue to use any data collected before such a withdrawal of consent. In addition, if a subject withdraws from the study, he/she may request destruction of any samples taken and not tested, and the Investigator must document this in the study center study records.

This document is confidential.

8.4.1 Lost to Follow-Up

A subject will be considered lost to follow-up if he or she repeatedly fails to return for scheduled visits and is unable to be contacted by the study center.

Reasonable efforts will be made to contact subjects who are lost to follow-up. These efforts must be documented in the subject's file.

9 TREATMENTS

Study treatment includes ibezapolstat, vancomycin, and placebo.

9.1 Ibezapolstat

9.1.1 Description

Ibezapolstat is a white to pale yellow powder. It is formulated as a 150 mg neat (without excipients) powder in white opaque, hard, size 1 gelatin capsules for oral administration.

9.1.2 Manufacturing

Ibezapolstat is manufactured by Piramal Enterprises Limited Pharmaceutical Development Services in Ahmedabad, India. Ibezapolstat will be supplied by Acurx Pharmaceuticals.

9.1.3 How Supplied

Segment 2A: Approximately 30 capsules are supplied in 40 cc round, white, opaque, high-density polyethylene bottles with integral seals and caps. Individual doses will be taken from these bottles according to the coded dosing schedule.

Segment 2B: Individual gelatin capsules will be in blister packs and each dose labeled as "Day X (Day 1 to 10) Dose X (Dose 1 to 4)".

9.1.4 Packaging and Labeling

All packaging and labeling will be done according to Good Manufacturing Practice and the relevant regulatory requirements. Packaging will be designed to maintain the double-blind nature of the trial.

9.1.5 Storage and Handling

Ibezapolstat is to be stored at ambient temperature (23 ºC, range 20 ºC to 25 ºC). Keep bottle tightly closed before and after each use.

All study treatments must be stored in a secure, environmentally controlled and monitored area in accordance with the labeled storage conditions with access limited to the Investigator, appropriate pharmacists, and authorized study center staff.

9.1.6 Dietary and Lifestyle Restrictions

In the Phase 1 study, the food effect on ibezapolstat following a single dose of ibezapolstat showed a peak plasma concentration of ibezapolstat of approximately 3 times greater in a fasting state versus a fed state. Therefore, to minimize systemic absorption and maximize colonic concentrations, subjects in Segment 2B will be instructed to take their morning and

evening doses (every 12 hours) of study drug with food; subjects will record their fed or fasting status for each dose. The requirement to take study drug with food only affects ibezapolstat but not vancomycin or placebo; therefore, only morning and evening doses must be taken with food by all subjects in this blinded trial. There is no dietary requirement for the other two daily dosing times.

9.2 Vancomycin

Vancomycin is commercially available as vancomycin hydrochloride 125-mg capsules. Vancomycin capsules will be over-encapsulated with gelatin capsules to be identical in appearance to ibezapolstat.

Vancomycin will be packaged in blister packs identical to ibezapolstat with each dose labeled as "Day X Dose X."

Vancomycin will be administered according to instructions in the package insert. Vancomycin is administered orally as 125 mg every 6 hours. Although the requirement to take study drug with food only affects ibezapolstat, not vancomycin or placebo, to maintain the blind in Segment 2B, only morning and evening doses must be taken with food; subjects will record their fed or fasting status for each dose. There is no dietary requirement for the other 2 daily dosing times.

9.3 Placebo

Placebo powder will be filled into gelatin capsules to be identical in appearance to ibezapolstat. See Section 9.4 for placebo dosing instructions.

The placebo capsules are manufactured by Piramal Enterprises Limited Pharmaceutical Development Services in Ahmedabad, India and will be supplied by Acurx Pharmaceuticals.

Packaging, labeling, storage, and handling of placebo will be the same as for ibezapolstat.

9.4 Dosage Schedule

Segment 2A

In this open-label segment, ibezapolstat will be administered to all enrolled subjects as 450 mg (three 150 mg capsules) every 12 hours for 10 days. Study drug will be administered with 240 mL water. There are no dietary requirements for this part of the study.

Segment 2B

Subjects randomly assigned to ibezapolstat will receive 450 mg ibezapolstat (three 150 mg capsules) orally every 12 hours for 10 days. Blinding is maintained in Segment 2B by over-encapsulation of all study drugs to appear identical. Because ibezapolstat and vancomycin have different dosing regimens (every 12 hours versus every 6 hours, respectively), subjects randomized to ibezapolstat will receive a placebo twice a day opposite of ibezapolstat dosing to mimic vancomycin dosing every 6 hours in order to maintain the blind. Thus, subjects assigned to ibezapolstat will receive ibezapolstat every 12 hours (eg,

This document is confidential.

6 AM and 6 PM) and a placebo capsule (eg, 12 AM and 12 PM) every 12 hours. Subjects randomized to vancomycin will receive 1 vancomycin capsule and 2 placebo capsules to mimic the 3 ibezapolstat 150 mg capsules every 12 hours (eg, 6 AM and 6 PM) and a single vancomycin capsule (eg, 12 AM and 12 PM) every 12 hours.

Study drug will be administered with 240 mL water. The morning and evening doses (every 12 hours) of study drug must be taken with food. Subjects will record their fed or fasting status for each dose in the subject diary.

Table 1. Dose, Route, and Schedule of Study Medication for Segment 2B

Randomized Study Arm	Dose[a]	Duration of Treatment (Days)	Active Capsules per Day	Placebo Capsules per Day	Route
Ibezapolstat	450 mg	10	6	2	Oral
Vancomycin	125 mg	10	4	4	Oral

[a] Subjects will take study drug 4 times per day (approximately every 6 hours) at breakfast, lunch, dinner, and bedtime.

Table 2. Daily Dosing for Segment 2B

Study Arm	Dose 1[a]	Dose 2	Dose 3[a]	Dose 4
Ibezapolstat	Ibezapolstat 450 mg oral capsule (given as three 150 mg capsules)	Placebo	Ibezapolstat 450 mg oral capsules (given as three 150 mg capsules)	Placebo
Vancomycin	Vancomycin 125 mg oral capsule (1 vancomycin capsule + 2 placebo capsules)	Vancomycin 125 mg oral capsule	Vancomycin 125 mg oral capsule (1 vancomycin capsule + 2 placebo capsules)	Vancomycin 125 mg oral capsule

[a] Subjects will take study drug with food.

9.5 Measures to Minimize Bias: Study Treatment Assignment and Blinding

9.5.1 Method of Study Treatment Assignment

Segment 2A

Subjects will be screened and enrolled in the order they present to the study centers and will be assigned the next available sequential subject number. Subject number will be assigned sequentially by electronic data capture (EDC).

Segment 2B

On Day 1, subjects will be randomly allocated to kits containing blister packs of either ibezapolstat or vancomycin and will be assigned a randomization number according to an

interactive web response system (IWRS). The randomization schedule will be produced by staff outside the study team and kept secure from blinded study staff until after the study database is locked. Site staff will give the kits containing the blister packs to hospital staff while the subject is in the hospital (if needed), or to the subject to take home for self-dosing.

9.5.2 Blinding

Segment 2A

Segment 2A is an open-label study; therefore, blinding is not applicable.

Segment 2B

Segment 2B is a double-blind study. Ibezapolstat, placebo, and vancomycin capsules will be identical in physical appearance by over-encapsulation, packaged into blister packs with each dose labeled, and will be administered 4 times a day so that neither study staff nor the subjects will know to which treatment the subject was assigned. Subjects randomized to vancomycin will receive 3 capsules (1 vancomycin capsule and 2 placebo capsules) to mimic the 3 ibezapolstat 150 mg capsules every 12 hours (eg, 6 AM and 6 PM) and a single vancomycin capsule (eg, 12 AM and 12 PM) every 12 hours. The Investigator, study staff, Sponsor, Trial Oversight Committee, and the subject will remain blinded to study treatment throughout the study.

According to the randomization schedule as indicated in the Schedule of Assessments (Table 4), the Investigator or designee will obtain the kit number from the IWRS for the subject. The pharmacist or designee will provide the assigned kit number to the subject. No study site personnel, subjects, Sponsor personnel, or Sponsor designees will be unblinded to treatment assignment throughout the duration of the study unless unblinding is required. If an Investigator becomes unblinded to a given subject's study treatment, that subject will continue in the study and undergo all protocol-required procedures for the determination of safety and efficacy.

In the event that emergency unblinding is required for a given subject because of AEs or concerns for the subject's safety or wellbeing, the Investigator may break the randomization code for the subject via the IWRS, by which system the unblinding will be captured. The Investigator is responsible for notifying the Medical Monitor and/or Sponsor of such an event within 24 hours. The unblinding and its cause will also be documented in the eCRF.

9.6 Dosage Modification

Doses of study drug may not be modified in this study.

9.7 Treatment Accountability and Compliance

The pharmacist or other designated individual will maintain records of drug kit number delivered to the study site (including amounts and lot numbers received, and confirmation of appropriate temperature conditions during transit), the inventory at the study site, the distribution to and use by each subject, and the return of materials to the Sponsor for storage

or disposal. These records should include dates, quantities, batch/serial numbers, expiration dates, in-clinic temperature log, and unique code numbers assigned to the product and study subjects.

If the subject is hospitalized, administration of all study drug will be supervised by study site personnel to ensure compliance. Only subjects enrolled in the study may receive study drug and only authorized study center staff may administer the study drug to the subject. The subject will enter the date and time of dose administration and indicate fed or fasting status for each dose in the subject diary.

If the subject is discharged or not hospitalized, at each visit after initiation of treatment, study site personnel will record compliance of the subject with the subject's assigned regimen. Subjects will receive a diary and are to record the time and date of each dose they take in the diary. Subjects will be instructed to bring their diaries and drug kits containing unused/partially used/empty blister packs back for inspection at each study visit, as compliance will be assessed by capsule counts and recorded in the eCRF. Subjects are to be reminded of the importance of compliance with their assigned regimen, with an emphasis on taking their study drug on schedule and maintaining the prescribed interval between doses.

Discontinuation for noncompliance is at the Investigator's discretion and is to be noted in the eCRF.

Study site personnel will be instructed to keep unused study drug and empty study drug packaging for drug accountability. Investigational product will not be returned to the Sponsor until accountability has been fully monitored. Any unused study treatment material may be destroyed by the study center according to their standard operating procedures upon written approval of the Sponsor.

Investigators will maintain records that adequately document that the subjects were administered study drug from the correct study treatment kits and reconcile the products received from the drug dispensing center.

The Investigator, institution, or the head of the medical institution (where applicable) is responsible for study treatment accountability, reconciliation, and record maintenance (ie, receipt, reconciliation, and final disposition records).

9.8 Prior and Concomitant Therapy

9.8.1 Prior Medications

Restricted prior therapies are provided in Section 8.2. Any medication or vaccine (including over-the-counter or prescription medicines, vitamins, and/or herbal supplements) that the subject took within 30 days before, or is currently receiving at the Screening visit, must be recorded in the eCRF along with:

- Reasons for use
- Dates of administration including start and end dates

- Dosage information including dose and frequency

Because of the seriousness of CDI, subjects need to start on an anti-*C. difficile* antibacterial drug as soon as possible once a CDI is suspected. However, subjects cannot have received more than 24 hours of an anti-*C. difficile* antibacterial medication before study drug treatment. Subjects also cannot have received more than 48 hours of any anti-*C. difficile* antibacterial for the current CDI episode within 14 days before the first dose of study drug. Doing so may mask the treatment effect of ibezapolstat.

9.8.2 Concomitant Medications

All medications and other treatments taken by the subject during the study, including over-the-counter or prescription medicines, vitamins, and/or herbal supplements, must be recorded in the eCRF. The entry must include the dose, regimen, route, indication, and dates of use.

Refer to Table 3 for a list of medications that may alter bowel motility or impact CDI status and hence have the potential to confound study results. Control over administration of these medications is necessary, considering the primary endpoint of the study, but it is understood that a medication should not be withheld for purposes of the study if an alternative and comparable treatment cannot be provided, and the subject's health would otherwise be compromised.

From baseline/Day 1 to Day 12/TOC visit, confounding medications are NOT permitted. Any confounding medications taken should be recorded in the eCRF.

From the Day 12/TOC visit to Day 38, confounding medications, except for CDI medication for recurrence, should be AVOIDED. Any confounding medications taken during this time should be recorded in the eCRF.

Throughout the study, if antimicrobial therapy is required for infections other than those because of *C. difficile*, antimicrobials without activity/efficacy against *C. difficile* should be prescribed where possible without compromising the medical care of the subject.

This document is confidential.

Table 3. Potentially Confounding Medications

Confounding Medications	Screening	Treatment Segment	Follow-Up Segment	Recurrence Segment
Antimicrobials for treatment of CDI[a]	Maximum of 24 hrs for current episode of CDI	Not allowed	Not allowed	Permitted for treatment of confirmed recurrence
Antimicrobials that may be active against *C. difficile*[b]	No more than 24 hrs immediately before study drug treatment	Not allowed	Not allowed[c]	As medically required
Antimicrobials NOT active against *C. difficile*[d]	Allowed	Allowed	Allowed	Allowed
Anti-diarrheals, antiperistaltics	Not allowed	Not allowed	As medically required	As medically required
Opiates	Allowed if dose stable, decreasing or stopped 7 days before study drug treatment	Allowed if dose stable, decreasing or stopping is likely[e]	Allowed if dose stable, decreasing or stopping is likely[e]	Allowed following confirmed recurrence
Anti-toxin antibodies including bezlotoxumab	No prior use	Not allowed	Not allowed	Allowed following confirmed recurrence
Probiotics and herbal products for treatment of CDI	Not allowed	Not allowed	Not allowed	Allowed following confirmed recurrence
Fecal microbiota transplant	If taken within 3 months before study drug treatment, subject is excluded	Not allowed	Not allowed	Allowed following confirmed recurrence
IMP for CDI	If taken within 3 months before study drug treatment, subject is excluded	Not allowed	Not allowed	Allowed following confirmed recurrence
Intravenous immunoglobulin	If taken within 3 months before study drug treatment, subject is excluded	Not allowed	Not allowed	Allowed following confirmed recurrence
Investigational vaccine for *C. difficile*	Subject is excluded	Not allowed	Not allowed	Allowed following confirmed recurrence
IMP for non-CDI	If taken within 1 month (or 5 half-lives) before study drug treatment, subject is excluded	Not allowed	Not allowed	Allowed following confirmed recurrence
Treatment for active malignancy[f]	If taken within 6 months before study drug treatment (or likely to be taken during study), subject is excluded	Not allowed	Not allowed	Allowed following confirmed recurrence
Chemotherapy	If taken within 3 months before study drug treatment (or likely to be taken during study), subject is excluded	Not allowed	Not allowed	Allowed following confirmed recurrence

This document is confidential.

Confounding Medications	Screening	Treatment Segment	Follow-Up Segment	Recurrence Segment
Immunosuppressive therapy[g]	If current or likely to be taken during study, subject is excluded	Not allowed	Not allowed	Allowed following confirmed recurrence

Abbreviations: CDI, *C. difficile* infection; eCRF, electronic case report form; hrs, hours; IMP, investigational medicinal product.

[a] Includes but not limited to oral vancomycin, IV or oral metronidazole, fidaxomicin.

[b] Includes but not limited to oral teicoplanin, tigecycline, oral rifamycins, fusidic acid, bacitracin.

[c] If antibiotics are required for non-CDI infection prescribe antibiotics that are not active versus *C. difficile.*

[d] Document all antibiotics given in eCRF.

[e] Consult with Medical Monitor if required.

[f] Chemotherapy, radiotherapy, or biological treatment.

[g] Does not include the limited topical or inhaled use of steroids or a brief period of oral steroids equivalent to a daily dose of less than 40 mg of prednisone administered for less than 2 weeks.

This document is confidential.

Any potential confounding medications taken 4 weeks before study drug treatment should be recorded in the eCRF along with any potential confounding medications started, stopped, or changed during the study (up to Day 38).

During the study, both the treatment period and follow-up period, subjects who take any confounding medications should not necessarily be discontinued from study treatment and should not be withdrawn from the study.

Investigators should contact the Medical Monitor for any questions regarding concomitant or prior therapy.

10 STUDY PROCEDURES

Table 4 outlines the timing of procedures and assessments to be performed throughout the study. Section 12.5 specifies laboratory assessment samples to be obtained. See Section 11 and Section 12 for additional details regarding efficacy assessments and safety assessments, respectively.

Table 4. Schedule of Assessments

<table>
<tr><th>Study Phase</th><th>Screening</th><th colspan="5">Treatment</th><th colspan="4">Follow-Up</th><th>Recurrence</th><th colspan="2">Extended Follow-Up[i]</th></tr>
<tr><th>Assessment</th><th>Screening Visit[a]</th><th>Baseline[a]</th><th>Check In</th><th>Check In</th><th>Check In</th><th>End of Treatment</th><th>Test of Cure</th><th>Check In</th><th>Check In</th><th>End of Study</th><th>Suspected Recurrence</th><th>Obs Visit</th><th>Obs Visit</th></tr>
<tr><td>Day</td><td>Day -2 to Day 1</td><td>Day 1</td><td>Day 3</td><td>Day 5</td><td>Day 8</td><td>Day 10</td><td>Day 12 (≥ 48 hrs post last dose)</td><td>Day 20</td><td>Day 30</td><td>Day 38</td><td>Test of Cure to End of Study[j]</td><td>Day 66</td><td>Day 94</td></tr>
<tr><td>Visit Window</td><td></td><td></td><td>±1 Day</td><td>±1 Day</td><td>±1 Day</td><td>±1 Day</td><td>+1 Days</td><td>±2 Days</td><td>±2 Days</td><td>±2 Days</td><td><48 hrs of Reporting</td><td>±2 Days</td><td>±2 Days</td></tr>
<tr><td>Visit Format</td><td>Clinic</td><td>Clinic</td><td>Tel.</td><td>Clinic</td><td>Tel.</td><td>Tel.</td><td>Clinic</td><td>Tel.</td><td>Tel.</td><td>Clinic</td><td>Clinic</td><td>Clinic</td><td>Clinic</td></tr>
<tr><td>Segment 2A/2B</td><td></td><td></td><td></td><td></td><td></td><td></td><td></td><td></td><td></td><td></td><td></td><td></td><td></td></tr>
<tr><td>Informed consent</td><td>X</td><td></td><td></td><td></td><td></td><td></td><td></td><td></td><td></td><td></td><td></td><td></td><td></td></tr>
<tr><td>Inclusion/exclusion criteria</td><td>X</td><td></td><td></td><td></td><td></td><td></td><td></td><td></td><td></td><td></td><td></td><td></td><td></td></tr>
<tr><td>CDI diagnosis including free toxin test[b]</td><td>X</td><td></td><td></td><td></td><td></td><td></td><td></td><td></td><td></td><td></td><td>X</td><td></td><td></td></tr>
<tr><td>Demographics</td><td>X</td><td></td><td></td><td></td><td></td><td></td><td></td><td></td><td></td><td></td><td></td><td></td><td></td></tr>
<tr><td>Medical & medication histories including detailed CDI history</td><td>X</td><td></td><td></td><td></td><td></td><td></td><td></td><td></td><td></td><td></td><td></td><td></td><td></td></tr>
<tr><td>Physical examination[c]</td><td>X</td><td></td><td></td><td></td><td></td><td></td><td></td><td></td><td></td><td></td><td></td><td></td><td></td></tr>
<tr><td>Height</td><td>X</td><td></td><td></td><td></td><td></td><td></td><td colspan="5"></td><td></td><td></td></tr>
<tr><td>Vital signs</td><td></td><td>X</td><td></td><td>X</td><td></td><td></td><td colspan="5">X[d]</td><td></td><td></td></tr>
<tr><td>Safety laboratory tests[e]</td><td></td><td>X</td><td></td><td>X</td><td></td><td></td><td>X</td><td colspan="4"></td><td></td><td></td></tr>
<tr><td>Pregnancy test[f]</td><td>X</td><td></td><td></td><td></td><td></td><td></td><td>X</td><td></td><td></td><td></td><td></td><td></td><td></td></tr>
</table>

This document is confidential.

Study Phase	Screening	Treatment					Follow-Up				Recurrence	Extended Follow-Up[i]	
**Assessment**	**Screening Visit[a]**	**Baseline[a]**	**Check In**	**Check In**	**Check In**	**End of Treatment**	**Test of Cure**	**Check In**	**Check In**	**End of Study**	**Suspected Recurrence**	**Obs Visit**	**Obs Visit**
**Day**	**Day -2 to Day 1**	**Day 1**	**Day 3**	**Day 5**	**Day 8**	**Day 10**	**Day 12 (≥ 48 hrs post last dose)**	**Day 20**	**Day 30**	**Day 38**	**Test of Cure to End of Study[j]**	**Day 66**	**Day 94**
**Visit Window**			**±1 Day**	**±1 Day**	**±1 Day**	**±1 Day**	**+1 Days**	**±2 Days**	**±2 Days**	**±2 Days**	**<48 hrs of Reporting**	**±2 Days**	**±2 Days**
**Visit Format**	**Clinic**	**Clinic**	**Tel.**	**Clinic**	**Tel.**	**Tel.**	**Clinic**	**Tel.**	**Tel.**	**Clinic**	**Clinic**	**Clinic**	**Clinic**
Electrocardiogram		X		X									
Dosing		◄──	───	───	───	──►							
Investigator assessment of clinical response							X			X			
Concomitant medications	◄──	───	───	───	───	───	───	───	───	───	──►		
Adverse events	◄──	───	───	───	───	───	───	───	───	───	──►		
Subject diary	◄──	───	───	───	───	───	───	───	───	───	──►		
Stool samples for future analyses[g] (microbiome and fecal drug concentration analyses)	X		X	X	X	X	X	X	X	X	X		
Blood sample for pharmacokinetics[h]		X		X									
**Segment 2B only**													
Randomization per IWRS		X											
EQ-5D-5L	X						X			X	X	X	X

This document is confidential.

**Study Phase**	**Screening**	**Treatment**					**Follow-Up**				**Recurrence**	**Extended Follow-Up[i]**	
**Assessment**	**Screening Visit[a]**	**Baseline[a]**	**Check In**	**Check In**	**Check In**	**End of Treatment**	**Test of Cure**	**Check In**	**Check In**	**End of Study**	**Suspected Recurrence**	**Obs Visit**	**Obs Visit**
**Day**	**Day -2 to Day 1**	**Day 1**	**Day 3**	**Day 5**	**Day 8**	**Day 10**	**Day 12 (≥ 48 hrs post last dose)**	**Day 20**	**Day 30**	**Day 38**	**Test of Cure to End of Study[j]**	**Day 66**	**Day 94**
**Visit Window**			**±1 Day**	**±1 Day**	**±1 Day**	**±1 Day**	**+1 Days**	**±2 Days**	**±2 Days**	**±2 Days**	**<48 hrs of Reporting**	**±2 Days**	**±2 Days**
**Visit Format**	**Clinic**	**Clinic**	**Tel.**	**Clinic**	**Tel.**	**Tel.**	**Clinic**	**Tel.**	**Tel.**	**Clinic**	**Clinic**	**Clinic**	**Clinic**
Medical Resource Utilization Questions	X		X	X	X	X	X	X	X	X	X		
Stool samples for future analyses (microbiome analyses)												X	X

Abbreviations: CDI, *Clostridioides difficile* infection; IWRS, Interactive Web Response System; Obs, observation; Tel., telephone.

[a] May occur the same day as the baseline visit. Written informed consent for the study will be obtained from all subjects before any protocol specific procedures are performed.

[b] Any Sponsor-approved free toxin test may be used.

[c] A complete physical at screening; limited physical examination at other times as clinically indicated.

[d] Perform at Day 38 or earlier (at an unscheduled visit) if subject experiences a confirmed recurrence.

[e] Safety laboratory tests will include biochemistry, hematology, and urinalysis.

[f] Only in women of childbearing potential. Serum test will be done at Screening; urine test will be done at the site at other specified time points.

[g] If subject does not attend clinic then arrangements must be made to collect stool sample from subject's home. Sample must be collected before first dose of study drug. Stool samples should be kept cool and brought to the clinic within 24 hours after collection.

[h] Pharmacokinetic blood samples will be collected 2 hours (± 15 minutes) and 4 hours (± 30 minutes) after first dose of the day. In Segment 2B, pharmacokinetic blood samples will be collected in approximately 50% of subjects (ie, approximately 36 subjects).

[i] Randomized Segment 2B subjects recruited from selected sites will have the opportunity to participate in an extended follow-up period to Day 94

[j] End of study includes observation visits Day 66 and Day 94 for those subjects participating in the extended follow-up period

This document is confidential.

10.1 Informed Consent

Before performing any study-related procedures, the Investigator (or designee) will obtain written informed consent from the subject.

In the event that rescreening occurs, the individual is required to sign a new ICF and must be assigned a new identification number.

10.2 Study Procedures

Assessments and their timing are to be performed as outlined in the Schedule of Assessments (Table 4). Section 12.5 specifies laboratory assessment samples to be obtained.

Assessments and procedures scheduled at a visit where study drug is administered should be performed before administration of treatment unless otherwise indicated in the Schedule of Assessments (Table 4).

Efficacy assessments are described in Section 11 and include provision of subject diaries, assessment of clinical response, measurements of plasma and fecal ibezapolstat concentrations at specific concentrations, characterization of the fecal microbiome, assessment of recurrence, measurement of time to resolution of diarrhea, and measurement of time to hospital discharge.

Safety assessments are described in Section 12 and include vital signs, physical examinations, ECGs, clinical laboratory assessments, pregnancy tests, concomitant medications, and AEs.

The Investigator may, at his/her discretion, arrange for a subject to have an unscheduled assessment, especially in the case of AEs that require follow-up or are considered by the Investigator to be possibly related to the use of study drug. The unscheduled visit page in the eCRF must be completed.

Study discontinuation procedures are described in Section 8.4.

Follow-up of AEs and SAEs leading to study discontinuation is discussed in Section 8.4.

11 EFFICACY ASSESSMENTS

The Schedule of Assessments (Table 4) outlines the efficacy assessments to be performed throughout the study and their timing.

11.1 Subject Diary

Subjects will be provided with a diary and completion instruction at the baseline visit (Day 1). Adequate time should be allocated on Day 1 to ensure that the subject understands how to complete the diary and is willing and able to do so every day throughout the study through Day 38/end of study visit. NOTE: The primary objective and endpoint for this study relies on subject reported data collected via the diary. It is critical that the Investigator and subject understand that accurate, complete, and contemporaneous diary data is required to meet the study primary objective. It is particularly important to ensure that each subject completes the diary

every day. The diary will have reminders for subjects to take the study drug and to complete the diary daily.

Subjects will be asked to record the following details in their diary:

- Date and time of each dose of study drug and if it was taken with food
- Date, time, and consistency (using the Bristol Stool Chart) of all bowel movements (up to Day 12/TOC)
- Date, time, and consistency of all UBMs from TOC to Day 38. Subjects will be required to answer a question every day in the diary to indicate if UBMs were experienced and to record the details of the UBMs if they occurred.

Information from the diary will be used directly for analysis of the primary and secondary endpoints. The Investigator or designee will be required to monitor the subject's diary entries and contact the subject to discuss any omissions or inaccurate entries.

Data changes that constitute subject reported data are not permitted. Changes to diary data will be limited to administrative corrections such as erroneous subject number, visit date, subject status, and elimination of duplicate data or merging of data for the same subject.

Subject reported diary errors will be reported as protocol deviations.

Investigators or designated site staff will be responsible to have a missed diary entry completed by asking the subject about the missed data when the subject brings the diary to study visits.

Subjects who have difficulty completing the diary may be supported by a care provider either temporarily or for the duration of the study. This is permitted as long as the Investigator is confident that the support provided is adequate to ensure accurate entries into the diary on the subject's behalf. The Investigator must document agreement for a care provider to enter information into the diary on behalf of the subject and the rationale.

11.2 Clinical Cure

Clinical cure: survival and the resolution of diarrhea in the 24-hour period immediately before EOT that is maintained for 48 hours post EOT without a requirement for additional CDI treatment.

Treatment failures are defined as those subjects:

- Who do not respond with reduced diarrhea; or
- Whose diarrhea worsens during therapy; or
- Who do not remain free of diarrhea for at least 2 days after the end of therapy; or
- Who require an additional treatment for CDI before the TOC visit; or
- Who do not survive until the TOC visit.

Subjects who failed study treatment will be offered treatment for CDI according to standard of care antibiotic at the Investigator's discretion.

After EOT, all subjects will be followed for up to an additional 28 ± 2 days to assess the recurrence of CDI. There also will be post treatment observation visits on Day 66 and Day 94 for those subjects participating in the extended follow-up period.

11.3 Sustained Clinical Cure

Sustained clinical cure: no recurrence of diarrhea from TOC to Day 38 among the subset of subjects who achieved an initial clinical cure. Recurrence is defined as ≥ 3 UBMs in any 24-hour period with free *C. difficile* toxin A or B (or both) detected in a stool sample and, in the opinion of the Investigator, there is a need for CDI retreatment.

The Investigator's assessment of SCC will be based on the Investigator's decision that there is no need for further treatment for CDI based on post treatment symptoms and signs during the 28 ± 2 days post treatment.

11.4 Extended Clinical Cure

Extended clinical cure: no recurrence of diarrhea from TOC to Day 56 (ECC56) and Day 84 (ECC84) among the subset of subjects who achieved an initial clinical cure. Recurrence is defined as ≥ 3 UBMs in any 24-hour period with free *C. difficile* toxin A or B (or both) detected in a stool sample and, in the opinion of the Investigator, there is a need for CDI retreatment.

The Investigator's assessment of ECC will be based on the Investigator's decision that there is no need for further treatment for CDI based on post treatment symptoms and signs during the 28 ± 2 days post treatment.

11.5 Microbial Assessments

Microbiome testing: Stool samples will be collected at the time points outlined in Table 4 and analyzed for microbial quantity and diversity by polymerase chain reaction and Next Gen sequencing.

Subjects will be provided with kits for collecting stool samples at home. A cooling device will be included to preserve the sample during transport to the study clinic. Stool samples must be brought to the study clinic within 24 hours of collection.

11.6 Time to Resolution of Diarrhea

Time to resolution of diarrhea: the time from outset of treatment to the first formed bowel movement not followed within the next 24 hours by a UBM.

11.7 Time to Hospital Discharge

Time from outset of treatment to the day of discharge, and not followed by readmission for treatment of CDI before the TOC visit (for hospitalized subjects only).

12 SAFETY AND TOLERABILITY ASSESSMENTS

Safety and tolerability assessments (vital signs, physical examinations, ECG recording, AEs, clinical laboratory results [routine hematology, biochemistry, and urinalysis]), pregnancy tests,

This document is confidential.

and concomitant medications are to be performed at protocol-specified visits, as specified in the Schedule of Assessments (Table 4). Study subjects will be monitored carefully throughout each dosing period for adverse experiences. A qualified physician will assess the relationship of AEs to study drug and in general based on such considerations as temporal relationship to study drug administration, subject's relevant medical history, and whether the finding is likely because of a pre-existing condition.

12.1 Medical History

Medical history will be recorded at Screening. Investigators should document the occurrence, signs, and symptoms of the subject's pre-existing conditions, including all prior significant illnesses, up to and including 1 year before Screening. Additional pre-existing conditions present at the time when informed consent is given and up to the time of first dosing (Day 1) are to be regarded as concomitant. Medical history will include alcohol consumption and smoking history.

Illnesses first occurring or detected during the study and/or worsening of a concomitant illness during the study are to be documented as AEs in the eCRF in accordance with Section 12.6. All changes not present at baseline or described in the past medical history and identified as clinically noteworthy must be recorded as AEs.

Additionally, demographic data will be collected for all subjects and include age, sex, race, ethnicity, and CDI history.

12.2 Vital Signs

Vital signs (body temperature, respiration rate, heart rate, and systolic and diastolic blood pressure measurements) will be evaluated at the visits indicated in the Schedule of Assessments (Table 4). All vital signs will be measured after the subject has been resting in a sitting or supine position for at least 5 minutes. Blood pressure measurements are to be taken in the same arm for the duration of the study. Either an automated or manual measurement of blood pressure is acceptable; however, the same method should be used for the duration of the study. Body weight (without shoes) will be recorded whenever vital signs are recorded; height (without shoes) will be recorded at Screening only.

Vital sign measurements will be repeated if clinically significant or machine/equipment errors occur. Out-of-range blood pressure, respiratory rate, or heart rate measurements will be repeated at the Investigator's discretion. Any confirmed, clinically significant vital sign measurements must be recorded as AEs.

12.3 Physical Examination

A complete physical examination (head, eyes, ears, nose and throat; heart; lungs; abdomen; skin; cervical and axillary lymph nodes; and neurological and musculoskeletal systems) will be performed at Screening (Visit 1). Physical examinations will be performed by a physician.

A limited physical examination to verify continued subject eligibility and to follow-up regarding any change in medical history will be performed at the visits indicated in the Schedule of

Assessments (Table 4). Symptom-driven, limited physical examinations will be performed as clinically indicated at any study visit.

12.4 Electrocardiograms

A 12-lead, resting (sitting or supine for at least 10 minutes) ECG will be obtained at the visits indicated in the Schedule of Assessments (Table 4). Study sites may use their standard operating procedures for detailed instructions on conducting the ECG.

At Screening, the Investigator will examine the ECG traces for signs of cardiac disease that could exclude the subject from the study. An assessment of normal or abnormal will be recorded; if the ECG is considered abnormal, the abnormality will be documented in the eCRF. ECGs will be repeated if clinically significant abnormalities are observed or artifacts are present.

12.5 Laboratory Assessments

Laboratory assessment samples (Table 5) are to be obtained at designated visits as detailed in the Schedule of Assessments (Table 4).

Table 5. Laboratory Assessments

Hematology	Biochemistry	Urinalysis (Dipstick)
Full and differential blood count	Albumin	Appearance
Hct	ALT	pH
Hb	ALP	Protein
MCH	AST	Glucose
MCHC	BUN	Ketones (dipstick)
MCV	Creatinine	RBCs (dipstick)
Platelet count	Electrolytes (sodium, potassium, chloride, calcium, phosphorus)	Specific gravity
RBC count	Glucose	Urine HCG (premenopausal women only)
WBC count with differential	LDH	Microscopy (if needed)
	Total bilirubin	
	Direct bilirubin	
	Uric acid	
**Pregnancy test:** A pregnancy HCG test will be performed on all women of childbearing potential at Screening.		

Abbreviations: ALP, alkaline phosphatase; ALT, alanine aminotransferase; AST, aspartate aminotransferase; BUN, blood urea nitrogen; Hb, hemoglobin; HCG, human chorionic gonadotropin; Hct, hematocrit; LDH, lactate dehydrogenase; MCH, mean corpuscular hemoglobin; MCHC, mean corpuscular hemoglobin concentration; MCV, mean corpuscular volume; RBC, red blood cell; WBC, white blood cell.

Local laboratories will be used in Segment 2A. A central laboratory will be used for Segment 2B. Urine samples will be analyzed by dipstick, and a microscopic analysis will be performed if the results of dipstick indicate abnormalities to be further investigated. All laboratory reports must be reviewed, signed, and dated by the Investigator. A legible copy of all reports must be included in the subject's medical record (source document) for that visit and the site must enter laboratory results manually into EDC for each subject. Any laboratory test result

considered by the Investigator to be clinically significant should be considered an AE (clinically significant AEs include those that require an intervention). Clinically significant abnormal values occurring during the study will be followed up until repeat test results return to normal, stabilize, or are no longer clinically significant.

12.6 Adverse Events

An AE is any symptom, physical sign, syndrome, or disease that either emerges during the study or, if present at Screening, worsens during the study, regardless of the suspected cause of the event. All medical and psychiatric conditions (except those related to the indication under study) present at Screening will be documented in the medical history eCRF. Changes in these conditions and new symptoms, physical signs, syndromes, or diseases should be noted on the AE eCRF during the rest of the study. Clinically significant laboratory abnormalities should also be recorded as AEs. Surgical procedures that were planned before the subject enrolled in the study are not considered AEs if the conditions were known before study inclusion; the medical condition should be reported in the subject's medical history.

Recurrence of CDI is a disease-related event that is common in subjects with CDI and can be serious/life threatening. Because recurrent CDI is an important medical event, all recurrences of CDI will be reported to the Sponsor per the standard process for expedited reporting of SAEs.

Subjects will be instructed to report AEs at each study visit. All AEs are to be followed up until resolution or a stable clinical endpoint is reached.

Each AE is to be documented in the eCRF with reference to date of onset, duration, frequency, severity, relationship to study drug, action taken with study drug, treatment of event, and outcome. Furthermore, each AE is to be classified as being serious or nonserious. Changes in AEs and resolution dates are to be documented in the eCRF.

For the purposes of this study, the period of observation for collection of AEs extends from the time the subject gives informed consent through study completion, as indicated in the Schedule of Assessments (Table 4). Follow-up of the AE, even after the date of therapy discontinuation, is required if the AE persists until the event resolves or stabilizes at a level acceptable to the Investigator.

When changes in the intensity of an AE occur more frequently than once a day, the maximum intensity for the event should be noted. If the intensity category changes over a number of days, then those changes should be recorded separately (with distinct onset dates).

Specific guidelines for classifying AEs by intensity and relationship to study drug are given in Table 6 and Table 7.

Table 6. Classification of Adverse Events by Intensity

**MILD**: An event that is easily tolerated by the subject, causing minimal discomfort and not interfering with everyday activities.
**MODERATE**: An event that is sufficiently discomforting to interfere with normal everyday activities.

This document is confidential.

**SEVERE**: An event that prevents normal everyday activities.

Table 7. Classification of Adverse Events by Relationship to Study Drug

**UNRELATED**: This category applies to those AEs that are clearly and incontrovertibly due to extraneous causes (disease, environment, etc.).
**POSSIBLY**: This category applies to those AEs for which a connection with the test drug administration appears unlikely but cannot be ruled out with certainty. An AE may be considered possibly related if or when it meets 2 of the following criteria: (1) it follows a reasonable temporal sequence from administration of the drug; (2) it could not readily have been produced by the subject's clinical state, environmental or toxic factors, or other modes of therapy administered to the subject; or (3) it follows a known pattern of response to the test drug.
**PROBABLY**: This category applies to those AEs that the Investigator feels with a high degree of certainty are related to the test drug. An AE may be considered probably related if or when it meets 3 of the following criteria: (1) it follows a reasonable temporal sequence from administration of the drug; (2) it could not be reasonably explained by the known characteristics of the subject's clinical state, environmental or toxic factors, or other modes of therapy administered to the subject; (3) it disappears or decreases on cessation or reduction in dose (note that there are exceptions when an AE does not disappear upon discontinuation of the drug, yet drug-relatedness clearly exists; for example, as in bone marrow depression, fixed drug eruptions, or tardive dyskinesia); or (4) it follows a known pattern of response to the test drug.
**DEFINITELY**: This category applies to those AEs that the Investigator feels are incontrovertibly related to test drug. An AE may be assigned an attribution of definitely related if or when it meets all of the following criteria: (1) it follows a reasonable temporal sequence from administration of the drug; (2) it could not be reasonably explained by the known characteristics of the subject's clinical state, environmental or toxic factors, or other modes of therapy administered to the subject; (3) it disappears or decreases on cessation or reduction in dose and recurs with reexposure to drug (if rechallenge occurs); and (4) it follows a known pattern of response to the test drug.

Abbreviation: AE, adverse event.

12.6.1 Serious Adverse Events

An SAE is any untoward medical occurrence, in the view of either the Investigator or Sponsor, that:

- results in death,
- is life-threatening,
- results in inpatient hospitalization or prolongation of existing hospitalization,
- results in persistent or significant disability/incapacity, and/or
- is a congenital anomaly/birth defect.
- Other important medical events that may not be immediately life-threatening or result in death or hospitalization, based upon appropriate medical judgment, are considered SAEs if they are thought to jeopardize the subject and/or require medical or surgical intervention to prevent one of the outcomes defining an SAE.

SAEs are critically important for the identification of significant safety problems; therefore, it is important to take into account both the Investigator's and the Sponsor's assessment. If either the Sponsor or the Investigator believes that an event is serious, the event must be considered serious and evaluated by the Sponsor for expedited reporting.

This document is confidential.

12.6.2 Serious Adverse Event Reporting

An SAE occurring from the time informed consent is obtained, during the study, or within 30 days of stopping the treatment must be reported to the Syneos Health Safety and Pharmacovigilance group and will be communicated to the Sponsor. Any such SAE because of any cause, whether or not related to the study drug, must be reported within 24 hours of occurrence or when the Investigator becomes aware of the event. Notification can be made using the dedicated fax line or email for the Syneos Health pharmacovigilance group:

Syneos Health Safety and Pharmacovigilance fax number: 1-877-464-7787

Syneos Health Safety and Pharmacovigilance email address: safetyreporting@syneoshealth.com

If the Investigator contacts the Syneos Health pharmacovigilance group by telephone, then a written report must follow within 24 hours and is to include a full description of the event and sequelae in the format detailed in the SAE reporting form.

The event must also be recorded on the standard AE eCRF. Preliminary reports of SAEs must be followed up by detailed descriptions later on, including clear and anonymized photocopies of hospital case reports, consultant reports, autopsy reports, and other documents when requested and applicable. SAE reports must be made whether or not the Investigator considers the event to be related to the investigational drug.

Appropriate remedial measures should be taken to treat the SAE, and the response should be recorded. Clinical, laboratory, and diagnostic measures should be employed as needed in order to determine the etiology of the problem. The Investigator must report all additional follow-up evaluations to the Syneos Health Safety and Pharmacovigilance group within 24 hours of becoming aware of the additional information or as soon as is practicable. All SAEs will be followed up until the Investigator and Sponsor agree the event is satisfactorily resolved.

Any SAE that is not resolved by the end of the study or upon discontinuation of the subject’s participation in the study is to be followed up until it either resolves, stabilizes, returns to baseline values (if a baseline value is available), or is shown to not be attributable to the study drug or procedures.

12.6.3 Suspected Unexpected Serious Adverse Reactions

AEs that meet all of the following criteria will be classified as suspected unexpected serious adverse reactions (SUSARs) and reported to the appropriate regulatory authorities in accordance with applicable regulatory requirements for expedited reporting:

- serious
- unexpected (ie, the event is not consistent with the safety information in the Investigator’s Brochure)
- there is at least a reasonable possibility that there is a causal relationship between the event and the study treatment

This document is confidential.

The Investigator will assess whether an event is causally related to study treatment. The Sponsor (or Syneos Health) will consider the Investigator's assessment and determine whether the event meets the criteria for being reportable as a 7-day or 15-day safety report. SUSARs that are fatal or life-threatening must be reported to the regulatory authorities and the independent ethics committee (IEC)/institutional review board (IRB) (where required) within 7 days after the Sponsor (or Syneos Health) has first knowledge of them, with a follow-up report submitted within a further 8 calendar days. Other SUSARs must be reported to the relevant regulatory authorities and the IEC/IRBs within 15 calendar days after the Sponsor (or Syneos Health) first has knowledge of them.

The Sponsor (or Syneos Health) is responsible for reporting SUSARs and any other events required to be reported in an expedited manner to the regulatory authorities and for informing investigators of reportable events, in compliance with applicable regulatory requirements within specific timeframes. Investigators will notify the relevant IEC/IRBs of reportable events within the applicable timeframes.

12.6.4 Pregnancy

Women of childbearing potential (WOCBP) must have a negative pregnancy test at Screening. Following administration of study drug, any known cases of pregnancy in female subjects will be reported until the subject completes or withdraws from the study. The pregnancy will be reported immediately by faxing/emailing a completed pregnancy report to the Sponsor (or designee) within 24 hours of knowledge of the event. The pregnancy will not be processed as an SAE; however, the Investigator will follow-up with the subject until completion of the pregnancy and must assess the outcome in the shortest possible time but not more than 30 days after completion of the pregnancy. The Investigator should notify the Sponsor (or designee) of the pregnancy outcome by submitting a follow-up pregnancy report. If the outcome of the pregnancy involved spontaneous or therapeutic abortion (any congenital anomaly detected in an aborted fetus is to be documented), stillbirth, neonatal death, or congenital anomaly, the Investigator will report the event by faxing/emailing a completed pregnancy report form to the Sponsor (or designee) within 24 hours of knowledge of the event.

If the Investigator becomes aware of a pregnancy occurring in the partner of a subject participating in the study, the pregnancy should be reported to the Sponsor (or designee) within 24 hours of knowledge of the event. Information regarding the pregnancy must only be submitted after obtaining written consent from the pregnant partner. The Investigator will arrange counseling for the pregnant partner by a specialist to discuss the risks of continuing with the pregnancy and the possible effects on the fetus.

Upon discontinuation from the study, only those procedures that would not expose the subject to undue risk will be performed. The Investigator should also be notified of pregnancy occurring during the study but confirmed after completion of the study. In the event that a subject is subsequently found to be pregnant after inclusion in the study, any pregnancy will be followed to term, and the status of mother and child will be reported to the Sponsor after delivery.

This document is confidential.

12.6.5 Overdose

There is no specific antidote for ibezapolstat. The Investigator must immediately notify the Sponsor of any occurrence of overdose with study drug. Overdosage should be managed with symptomatic and supportive care.

12.7 EQ-5D-5L

The EQ-5D-5L is a questionnaire, which has been developed as a standardized measure of health status, which can provide a simple, generic measure of health for clinical and economic appraisal. It is cognitively undemanding, taking only a few minutes to complete. Subjects will be asked to complete the EQ-5D-5L at certain intervals in Segment 2B throughout the study, as specified in Table 4. Instructions to respondents are included in the questionnaire. The EQ-5D-5L essentially consists of 2 pages – the EQ-5D descriptive system (page 2) and the EQ visual analog scale (page 3). The EQ-5D-5L descriptive system comprises the following 5 dimensions: mobility, self-care, usual activities, pain/discomfort, and anxiety/depression.

If, at any visit, the subject is unable to complete the EQ-5D-5L independently, then it should not be completed for that visit.

12.8 Medical Resource Utilization Questions

Medical resource utilization data, associated with medical encounters, will be collected in the eCRF by the Investigator and study site personnel for all subjects throughout the study. Protocol-mandated procedures, tests, and encounters are excluded. Data collected will include:

- Length of hospital stay
- Hospital admissions and readmissions, and reasons for admission
- Subject location at admission and discharge

13 PHARMACOKINETICS

13.1 Pharmacokinetic Sampling

Instructions for the collection and handling of biological samples will be provided separately by the Sponsor or Sponsor designee and will be detailed in the Sample Processing Instructions.

Any changes in the timing or addition of time points for any planned study assessments must be documented and approved by the relevant study team member(s) and then archived in the Sponsor and study center study files but will not constitute a protocol amendment. The IRB will be informed of any safety issues that require alteration of the safety monitoring scheme or amendment of the ICF.

13.1.1 Blood Samples

For Segment 2A and approximately 50% of subjects in Segment 2B: Blood samples for PK analysis of ibezapolstat plasma levels will be collected at the time points indicated in the Schedule of Assessments (Table 4). The actual date and time of each blood sample collection will be recorded. The actual date and time of study treatment administration will also be

recorded. The timing of PK samples may be altered, and/or PK samples may be obtained at additional time points to ensure thorough PK monitoring. Blood samples will be taken either by direct venipuncture or an indwelling cannula inserted in a forearm vein.

Details of PK blood sample collection, processing, labeling, freezing and storage, and shipping procedures are provided in a separate laboratory manual.

13.1.2 Stool Samples

In conjunction with the drawing of multiple blood samples, multiple stool samples will be collected at the time points in Segment 2A and 2B indicated in the Schedule of Assessments (Table 4) for fecal concentrations of ibezapolstat through the TOC visit. The actual date and time of each fecal void will be recorded.

Subjects will be provided with kits for collecting stool samples at home. A cooling device will be included to preserve the sample. Stool samples must be brought to the study clinic within 24 hours of collection.

Details of stool sample collection, processing, labeling, storage, and shipping procedures are provided in the Sample Processing Instructions.

13.2 Pharmacokinetic Bioanalytical Methodology

The concentration of study drug will be determined from the plasma and stool samples using a validated bioanalytical method. Details of the method validation and sample analysis will be included with a separate bioanalytical report. PK samples will be collected in Segment 2A and approximately 50% of subjects in Segment 2B.

13.3 Calculation of Pharmacokinetic Variables

A population PK analysis may be conducted using plasma and fecal ibezapolstat concentrations. The model developed using this approach will be used to predict parameters such as, but not limited to, C_{max} and $AUC_{0\text{-}inf}$ for each subject. This analysis will be described in a separate PK analysis plan and results from the population PK analysis will also be presented in a separate report.

14 STATISTICAL ANALYSIS

The SAP will be prepared after the protocol is approved. This document will provide further details regarding the definition of analysis variables and analysis methodology to address all study objectives. The SAP will serve as a complement to the protocol and supersedes it in case of differences. The SAP will describe the subject analysis sets to be included in the analyses and procedures for handling missing data. This section is a summary of the planned statistical analyses of the endpoints.

The statistical analyses will be performed using SAS® software version 9.4 or higher. All data will be listed, and summary tables will be provided. Summary statistics will be presented by treatment group.

The following descriptive statistics will be used as applicable to summarize the study data unless otherwise specified:

- Continuous variables: sample size, mean, standard deviation, median, minimum, and maximum. Geometric mean and coefficient of variation will be included for relevant PK parameter summaries.
- Categorical variables: frequencies and percentages.

14.1 Determination of Sample Size

Segment 2A is considered a proof-of-principle substudy and is not hypothesis driven; the sample size of up to 20 subjects was selected to provide some degree of precision for the estimated cure rate, as shown in the table below:

Table 8. Estimated Cure Rate and Associated 95% Confidence Intervals Based on the Normal Approximation

Estimated Cure Rate (%)	95% Confidence Interval (%)
75	(56.0, 94.0)
80	(62.5, 97.5)
85	(69.4, 100.0)
90	(76.9, 100.0)

Sample size for Segment 2B was calculated based on the following assumptions:

1. Non-inferiority study
2. Endpoint is clinical cure at the TOC visit
3. One-sided normal approximation test
4. Overall alpha = 0.05
5. Allocation to treatment = 1:1
6. True cure rate = 0.80 in both arms
7. Non-inferiority margin = 0.25
8. Power = 80%
9. Sample size adjusted for one interim analysis (Pocock 1977).

A sample size of 36 subjects per treatment group, or 72 subjects total, will satisfy these criteria.

Further, subjects at selected sites will undergo extended follow-up to Day 94. At these selected sites, consecutive subjects will be offered the opportunity to participate in the extended follow-up period until approximately 16 subjects are recruited.

14.2 Analysis Populations

14.2.1 Enrolled Population

The Enrolled population will include all subjects who sign the ICF.

This document is confidential.

14.2.2 Intent-to-Treat Population

The intent-to-treat (ITT) population will include all subjects who are enrolled and treated in Segment 2A or who are randomized in Segment 2B. In Segment 2B, subjects will be analyzed based on their randomized assignment. The ITT population will be used as the primary analysis of efficacy.

14.2.3 Per Protocol Population

The PP population will consist of patients in the ITT population with no major protocol deviations. The determination of which subjects have major protocol violations will be made in a blinded manner before the database lock. For analyses based on the PP population, subjects will be grouped according to the treatment to which they were randomized. This population will be used for a secondary analysis of the primary and secondary efficacy endpoints.

14.2.4 ITT Extension Population

The ITT Extension population will include all randomized Segment 2B subjects who participate in the extended follow-up period. Subjects will be analyzed based on their randomized assignment. The ITT Extension population will be used for the analysis of the ECC56 and ECC84 exploratory endpoints.

14.2.5 Safety Population

The Safety population will include all subjects who receive at least 1 dose of study drug. Subjects will be analyzed based on the treatment actually received. This population will be used for the analysis of safety.

14.2.6 Pharmacokinetic Population

The PK population will include all subjects who take at least 1 dose of active study treatment and have at least 1 quantifiable plasma concentration collected post dose without important protocol deviations/violations or events thought to affect the PK.

14.3 Efficacy Analysis

14.3.1 Analysis of Primary Efficacy Endpoint

The primary efficacy endpoint is clinical cure at the TOC visit, defined as survival and resolution of diarrhea in the 24-hour period immediately before EOT that is maintained for 48 hours post EOT without a requirement for additional CDI treatment.

After EOT, subjects with initial clinical cure will be followed for up to an additional 28 ± 2 days to assess the recurrence of CDI.

For Segment 2A, this endpoint will be summarized using frequencies and percentages, together with a 95% exact (Clopper-Pearson) confidence interval for the true cure percentage.

For Segment 2B, the null and alternative hypotheses for this endpoint are as follows:

H_0: $\pi_{1,T} \leq \pi_{1,C} - \delta$

and

H_1: $\pi_{1,T} > \pi_{1,C} - \delta$,

where $\pi_{1,T}$ and $\pi_{1,C}$ are the true proportions of subjects with clinical cure at the TOC visit for the ibezapolstat 450 mg and vancomycin 125 mg treatments, respectively, and δ is the non-inferiority margin which is here taken to be 0.25.

This endpoint will be summarized by treatment group using frequencies and percentages, together with an exact (Clopper-Pearson) 95% confidence interval for the true cure percentage. The 95% confidence interval based on the normal approximation will also be calculated for the difference between the 2 population proportions ($\pi_{1,C} - \pi_{1,T}$). The null hypothesis will be tested using a one-sided normal approximation test non-inferiority at the 0.03037 significance level (Pocock 1977) for an overall significance level of 0.05. In the event non-inferiority of ibezapolstat 450 mg is demonstrated, Fisher's exact test (two-sided) will be used to conduct a test for superiority at the 0.05 significance level.

14.3.2 Analysis of Secondary Efficacy Endpoint: Sustained Clinical Cure

The secondary efficacy endpoint is SCC, which is defined below, and is determined for subjects who achieve initial clinical cure. The determination will be made at the Day 38 follow-up visit.

1. Recurrence is defined as ≥ 3 bowel movements with unformed stools in any 24-hour period with free *C. difficile* toxin A or B (or both) detected in a stool sample and in the opinion of the Investigator, there is a need for CDI retreatment.
2. SCC is defined as initial clinical cure without subsequent recurrence within 28 ± 2 days post EOT.

For Segment 2A, this endpoint will be summarized using frequencies and percentages, together with an exact (Clopper-Pearson) 95% confidence interval for the true cure percentage.

For Segment 2B, this endpoint will be summarized by treatment group using frequencies and percentages, together with an exact (Clopper-Pearson) 95% confidence interval for the true SCC percentage. A 95% confidence interval based on the normal approximation will also be calculated for the difference between the 2 population proportions ($\pi_{1,C} - \pi_{1,T}$). The null hypothesis that the true proportions are equal for the 2 treatments will be tested using a two-sided Fisher's exact test at the 0.05 significance level.

14.3.3 Analysis of Exploratory Endpoints

14.3.3.1 Microbial Assessments

Microbiome testing: Stool samples will be collected at the time points outlined in Table 4 and analyzed for microbial quantity and diversity by polymerase chain reaction and Next Gen sequencing.

Changes in the total fecal bacterial quantity and microbial diversity will be assessed for ibezapolstat-treated subjects in both segments, and in comparison to those treated with vancomycin in Segment 2B. Changes will be assessed during the treatment period and post EOT.

Details of the final microbiome analysis will be described in the SAP or relevant laboratory manual.

14.3.3.2 Time to Resolution of Diarrhea

For Segment 2A, time to resolution of diarrhea, defined as the time from onset of treatment to the first formed bowel movement not followed within the next 24 hours by a UBM, will be summarized using the Kaplan-Meier method, with estimates of the true resolution of diarrhea rate at Days 1, 5, 10, 12, and 40. The estimated median time to resolution of diarrhea, together with a 95% confidence interval, will also be presented. For Segment 2B, the analysis will be the same as for Segment 2A, except that the results will be presented by treatment group.

14.3.3.3 Time to Hospital Discharge

For Segment 2A, time to hospital discharge, defined as the time from onset of treatment to the day of discharge, and not followed by readmission for treatment of CDI before the TOC visit (for hospitalized subjects only), will be summarized using descriptive statistics. For Segment 2B, the analysis will be the same as for Segment A, except that the results will be presented by treatment group.

14.3.3.4 Quality of Life Assessments

The EQ-5D-5L scores for each of the 5 dimensions and the EQ-5D-5L visual analog scale score will be summarized by treatment group and time point using descriptive statistics.

Medical resource utilization data will be summarized by treatment group and time point using descriptive statistics for continuous variables and frequencies and percentages for categorical variables.

These analyses will be performed for Segment 2B only.

Further details of the statistical analyses of the EQ-5D-5L and Medical Resource Utilization will be described in the SAP.

14.3.3.5 ECC56 and ECC84

These endpoints will be summarized by treatment group using frequencies and percentages, together with exact (Clopper-Pearson) 95% confidence intervals for the true percentages. Also, 95% confidence intervals based on the normal approximation will be calculated for the differences between the 2 population proportions. These endpoints will be analyzed based on the ITT Extension Population.

14.4 Safety Analysis

TEAEs are defined as any AE with onset date on or after the date of the start of the study drug or any AE already present at baseline that worsens in severity. All reported AEs will be coded using the most recent version of the Medical Dictionary for Regulatory Activities (MedDRA). Events with missing onset dates will be considered to be treatment-emergent unless there is additional information indicating that the AE started before study treatment. Only TEAEs will be

summarized. If a subject experiences more than 1 occurrence of the same AE, the occurrence with the greatest severity and the closest relationship with the study drug will be used in the summary tables.

The number and percentage of subjects with at least one TEAE, at least one treatment-related TEAE, at least one serious TEAE, and at least one TEAE leading to study discontinuation will be presented by treatment group. AEs that are definitely, probably, or possibly related, or for which the relationship is missing, will be considered related.

The number and percentage of subjects having a TEAE in each System Organ Class (SOC) and having each individual type of AE (Preferred Term) will be presented. TEAEs will also be summarized at the event level by SOC/Preferred Term and severity. This will be done for all TEAEs, all serious TEAEs, and all TEAEs leading to discontinuation from the study.

All AEs will be listed by subject, along with information regarding onset, duration, relationship and severity to study drug, action taken with study drug, treatment of event, and outcome.

Clinical laboratory data and vital signs data will be summarized by treatment group and time point using descriptive statistics for both the actual values and the changes from baseline, as well as numbers and percentages of subjects with values outside the limits of the normal range at each time point. Electrocardiogram data will be summarized by treatment group and time point using descriptive statistics for both the actual values and the changes from baseline.

Concomitant medications initiated during the study period will be summarized by treatment group using frequencies and percentages.

14.5 Pharmacokinetic Analysis

The PK Analysis Set will be used for all summaries and exploratory analyses of PK data. Systemic exposure of ibezapolstat will be determined by measuring plasma ibezapolstat concentrations at specified time points following dose administration. Fecal concentrations of ibezapolstat will be measured at specified study visits. Plasma and fecal ibezapolstat concentrations will be listed by study segment, treatment, study day and/or nominal time, and summarized using descriptive statistics.

Summary statistics will be generated for ibezapolstat plasma and fecal concentrations. Given sufficient plasma and fecal levels over the lower limit of quantitation (LLOQ), the data will be analyzed as follows. Median (and range) and mean (and standard deviation) ibezapolstat plasma concentrations at each nominal time point will be calculated, as appropriate. Concentrations reported as below the LLOQ will be set to zero for descriptive statistics.

14.6 Interim Analysis

A single interim analysis will be performed when the first 36 Segment 2B subjects in the ITT Population (one half of the planned sample size) have data for the primary efficacy endpoint of clinical cure at the TOC visit. The null hypothesis (see Section 14.3.1) will be tested with a

one-sided normal approximation test of non-inferiority at a one-sided α=0.03037 level of significance (Pocock 1977) for an overall significance level of 0.05. If the p-value is less than or equal to 0.03037 at the time of the interim analysis, then the study may be stopped early for success.

14.7 Trial Oversight Committee

A Trial Oversight Committee will be established by the Sponsor to review accumulating safety data at regular intervals throughout the study and monitor overall study conduct. Members will include the Sponsor's Medical Monitor, Clinical Research Organization Medical Monitor, a biostatistician, and 1 PI who has enrolled subjects in Segment 2A. The Trial Oversight Committee can recommend in writing to the Sponsor whether to continue, modify, or stop the clinical study on the basis of efficacy and safety considerations. The Trial Oversight Committee's specific duties as well as statistical monitoring guidelines and procedures will be governed by a Trial Oversight Committee Charter.

After up to 10 subjects have been enrolled and completed the full course of treatment in Segment 2A, the Trial Oversight Committee will assess safety, tolerability, and as data allow, efficacy and will make a recommendation to the Sponsor regarding continued enrollment of Segment 2A.

Another assessment of safety, tolerability, and efficacy will be made by the Trial Oversight Committee after all subjects have been enrolled in Segment 2A and followed through at least the EOT visit. If the Trial Oversight Committee judges the results to be favorable, it will recommend to the Sponsor that Segment 2B commence, or alternatively may make other recommendations to the Sponsor to modify Segment 2A or the entire study, such as, for example, expanding Segment 2A, adding another open-label dose group, and/or prospectively adjusting the sample size of Segment 2B.

15 STUDY MANAGEMENT

15.1 Approval and Consent

15.1.1 Regulatory Guidelines

This study will be conducted in accordance with the accepted version of the Declaration of Helsinki and/or all relevant regulations, as set forth in Parts 50, 56, 312, Subpart D, of Title 21 of the US Code of Federal Regulations (CFR), in compliance with ICH and GCP guidelines and according to the appropriate regulatory requirements in the countries where the study was conducted.

15.1.2 Institutional Review Board/Independent Ethics Committee

Conduct of the study must be approved by an appropriately constituted IEC/IRB. Approval is required for the study protocol, protocol amendments (if applicable), Investigator's Brochure, ICFs, recruitment material and subject information sheets, and other subject-facing material.

15.1.3 Informed Consent

For each study subject, written informed consent will be obtained before any protocol-related activities. As part of this procedure, the PI or designee must explain orally and in writing the nature of the study, its purpose, procedures, expected duration, alternative therapy available, and the benefits and risks involved in study participation. The subject should be informed that he/she may withdraw from the study at any time, and the subject will receive all information that is required by local regulations and guidelines for ICH. The PI will provide the Sponsor or its representative with a copy of the IEC/IRB-approved ICF before the start of the study.

15.2 Data Handling

Any data to be recorded directly in the eCRFs (to be considered as source data) will be identified at the start of the study. Data reported in the eCRF that are derived from source documents should be consistent with the source documents, or the discrepancies must be explained. See also Section 15.3.

Clinical data will be entered by site personnel on eCRFs for transmission to the Sponsor. Data on eCRFs transmitted via the web-based data system must correspond to and be supported by source documentation maintained at the study site, unless the study site makes direct data entry to the databases for which no other original or source documentation is maintained. In such cases, the study site should document which eCRFs are subject to direct data entry and should have in place procedures to obtain and retain copies of the information submitted by direct data entry. All study forms and records transmitted to the Sponsor must only include coded identifiers such that directly identifying personal information is not transmitted. The primary method of data transmittal is via the secure, internet-based EDC system maintained by Syneos Health. Access to the EDC system is available to only authorized users via the study's internet web site, where a user unique assigned username and password are required for access.

Any changes made to data after collection will be made through the use of the EDC system. eCRFs will be considered complete when all missing and/or incorrect data have been resolved.

15.3 Source Documents

Source documents are considered to be all information in original records and certified copies of original records of clinical findings, observations, data, or other activities in a clinical study necessary for the reconstruction and evaluation of the study. The Investigator will provide direct access to source documents and/or source data in the facilitation of trial-related monitoring, audits, review by IECs/IRBs, and regulatory inspections.

The Investigator/institution should maintain adequate and accurate source documents and trial records that include all pertinent observations on each of the site's trial subjects. Source data should be attributable, legible, contemporaneous, original, accurate, and complete. Changes to source data should be traceable, not obscure the original entry, and be explained if necessary.

15.4 Record Retention

Study records and source documents must be preserved for at least 15 years after the completion or discontinuation of/withdrawal from the study, at least 2 years after the drug being studied has received its last approval for sale, or at least 2 years after the drug development has stopped, and in accordance with the applicable local privacy laws, whichever is the longer time period.

The Investigator agrees to comply with all applicable federal, state, and local laws and regulations relating to the privacy of subject health information, including, but not limited to, the Standards for Individually Identifiable Health Information, 45 CFR, Parts 160 and 164 (the Health Insurance Portability Accountability Act of 1996 Privacy Regulation). The Investigator shall ensure that study subjects authorize the use and disclosure of protected health information in accordance with Health Insurance Portability Accountability Act of 1996 Privacy Regulation and in a form satisfactory to the Sponsor.

15.5 Monitoring

The study will be monitored according to the study's monitoring plan to ensure that it is conducted and documented properly according to the protocol, GCP, and all applicable regulatory requirements.

On-site monitoring visits and contacts will be made at appropriate times during the study. The PI will assure he/she and adequate site personnel are available throughout the study to collaborate with clinical monitors. Clinical monitors must have direct access to source documentation in order to check the completeness, clarity, and consistency of the data recorded in the eCRFs for each subject.

The Investigator will make available to the clinical monitor all source documents and medical records necessary to review protocol adherence and eCRFs. In addition, the Investigator will work closely with the clinical monitor and, as needed, provide them appropriate evidence that the study is being conducted in accordance with the protocol, applicable regulations, and GCP guidelines.

15.6 Quality Control and Quality Assurance

The Sponsor or its designee will perform the quality assurance and quality control activities of this study; however, responsibility for the accuracy, completeness, security, and reliability of the study data presented to the Sponsor lies with the Investigator generating the data.

The Sponsor will arrange audits as part of the implementation of quality assurance to ensure that the study is being conducted in compliance with the protocol, standard operating procedures, GCP, and all applicable regulatory requirements. Audits will be independent of and separate from the routine monitoring and quality control functions. Quality assurance procedures will be performed at study sites and during data management to assure that safety and efficacy data are adequate and well documented.

This document is confidential.

15.7 Protocol Amendment and Protocol Deviation

15.7.1 Protocol Amendment

Amendments to the protocol that entail corrections of typographical errors, clarifications of confusing wording, changes in study personnel, and minor modifications that have no effect on the safety of subjects or the conduct of the study will be classed as administrative amendments and will be submitted to the IEC/IRB for information only. The Sponsor will ensure that acknowledgment is received and filed. Amendments that are classed as substantial amendments must be submitted to the appropriate regulatory authorities and the IECs/IRBs for approval and will not be implemented at sites until such approvals are received other than in the case of an urgent safety measure.

15.7.2 Protocol Deviations

Should a protocol deviation occur, the Sponsor must be informed as soon as possible. Protocol deviations and/or violations and the reasons they occurred will be included in the clinical study report. Reporting of protocol deviations to the IRB/IEC and in accordance with applicable regulatory authority mandates is an Investigator's responsibility.

15.8 Ethical Considerations

This study will be conducted in accordance with this protocol, the accepted version of the Declaration of Helsinki and/or all relevant federal regulations, as set forth in Parts 50, 56, 312, Subpart D, of Title 21 of the CFR; EU 536/2014, Annex 1, D, 17 (a); in compliance with GCP guidelines; and according to the appropriate regulatory requirements in the countries where the study is conducted.

IECs/IRBs will review and approve this protocol and the ICF. All subjects are required to give written informed consent before participation in the study.

15.9 Financing and Insurance

Before the study commences, the Sponsor (or its designee) and the Investigator (or the institution, as applicable) will agree on costs necessary to perform the study. This agreement will be documented in a financial agreement that will be signed by the Investigator (or the institution signatory) and the Sponsor (or its designee).

The Investigator is required to have adequate current insurance to cover claims for negligence and/or malpractice. The Sponsor will provide no-exclusion insurance coverage for the clinical study as required by national regulations.

15.10 Publication Policy/Disclosure of Data

Both the use of data and the publication policy are detailed within the clinical study agreement. Intellectual property rights (and related matters) generated by the Investigator and others performing the clinical study will be subject to the terms of a clinical study agreement that will be agreed between the institution and the Sponsor or their designee. With respect to such rights, the Sponsor or its designee will solely own all rights and interests in any materials, data, and

This document is confidential.

intellectual property rights developed by investigators and others performing the clinical study described in this protocol, subject to the terms of any such agreement. In order to facilitate such ownership, investigators will be required to assign all such inventions either to their institution or directly to the Sponsor or its designee, as will be set forth in the clinical study agreement.

16 REFERENCES

Acurx. ACX-362E. Investigator's Brochure Edition 1. White Plains, NY: Acurx; 12 October 2018.

Al-Nassir WN, Sethi AK, Nerandzic MM, Bobulsky GS, Jump RL, Donskey CJ. Comparison of clinical and microbiological response to treatment of Clostridium difficile-associated disease with metronidazole and vancomycin. Clin Infect Dis. 2008;47(1):56-62.

Global Markets Direct. Clostridium Infections – Pipeline Review, H2 2012, July 2012.

Dvoskin S, Xu WC, Brown NC, Yanachkov IB, Yanachkova M, Wright GE. A novel agent effective against Clostridium difficile infection. Antimicrob Agents Chemother. 2012;56(3):1624-6.

Kokkotou E, Moss AC, Michos A, Espinoza D, Cloud JW, Mustafa N, et al. Comparative efficacies of rifaximin and vancomycin for treatment of Clostridium difficile-associated diarrhea and prevention of disease recurrence in hamsters. Antimicrob Agents Chemother. 2008;52(3):1121-6.

Lessa FC, Winston LG, McDonald LC; Emerging Infections Program C, difficile Surveillance Team. Burden of Clostridium difficile infection in the United States. N Engl J Med. 2015;372(24):825-34.

Louie TJ, Cannon K, Byrne B, Emery J, Ward L, Eyben M, et al. Fidaxomicin preserves the intestinal microbiome during and after treatment of Clostridium difficile infection (CDI) and reduces both toxin reexpression and recurrence of CDI. Clin Infect Dis. 2012;55(Suppl 2):S132-42.

McDonald LC, Gerding DN, Johnson S, Bakken JS, Carroll KC, Coffin SE, et al. Clinical Practice Guidelines for Clostridium difficile Infection in Adults and Children: 2017 Update by the Infectious Diseases Society of America (IDSA) and Society for Healthcare Epidemiology of America (SHEA). Clin Infect Dis. 2018;66(7):987-94.

Nelson RL, Suda KJ, Evans CT. Antibiotic treatment for Clostridium difficile-associated diarrhoea in adults. Cochrane Database Syst Rev. 2017;3:CD004610. doi: 10.1002/14651858.CD004610.pub5.

Pocock SJ. Group sequential methods in the design and analysis of clinical trials. Biometrika. 1977;64 (2):191-199. doi.org/10.2307/2335684.

Razavi B, Apisarnthanarak A, Mundy LM. Clostridium difficile: emergence of hypervirulence and fluoroquinolone resistance. Infection. 2007;35(5):300-7.

Smits WK, Lyras D, Lacy DB, Wilcox MH, Kuijper EJ. Clostridium difficile infection. Nat Rev Dis Primers. 2016;2:16020. doi: 10.1038/nrdp.2016.20.

This document is confidential.

Torti A, Lossani A, Savi L, Focher F, Wright GE, Brown NC, et al. Clostridium difficile DNA polymerase IIIC: basis for activity of antibacterial compounds. Curr Enzym Inhib. 2011;7(3):147-53.

Xu WC, Wright GE, Brown NC, Long ZY, Zhi CX, Dvoskin S, et al. 7-Alkyl-N2-substituted-3-deazaguanines. Synthesis, DNA polymerase III inhibition and antibacterial activity. Bioorg Med Chem Lett. 2011;21(14):4197-202.

Zilberberg MD, Shorr AF, Kollef MH. Increase in adult Clostridium difficile-related hospitalizations and case-fatality rate, United States, 2000-2005. Emerg Infect Dis. 2008;14(6):929-31.

17 APPENDICES

APPENDIX 1 describes the contraception guidelines applicable for this study.

APPENDIX 2 contains the Bristol Stool Chart.

This document is confidential.

APPENDIX 1. CONTRACEPTION GUIDELINES

Definitions:

Woman of Childbearing Potential

A woman is considered fertile following menarche and until becoming postmenopausal unless permanently sterile (see below)

Women in the following categories are not considered WOCBP:

1. Premenarchal
2. Premenopausal female with 1 of the following:
    a. Documented hysterectomy
    b. Documented bilateral salpingectomy
    c. Documented bilateral oophorectomy

    Note: Documentation can come from the site personnel's review of the subject's medical records, medical examination, or medical history interview.
3. Postmenopausal female
    a. A postmenopausal state is defined as no menses for 12 months without an alternative medical cause. A high follicle stimulating hormone (FSH) level in the postmenopausal range may be used to confirm a postmenopausal state in women not using hormonal contraception or hormonal replacement therapy (HRT). However, in the absence of 12 months of amenorrhea, a single FSH measurement is insufficient.
    b. Females on HRT and whose menopausal status is in doubt will be required to use one of the non-estrogen hormonal highly effective contraception methods if they wish to continue their HRT during the study. Otherwise, they must discontinue HRT to allow confirmation of postmenopausal status before study enrollment.

Contraception Guidance:

Male Subjects

Are abstinent from penile-vaginal intercourse as their usual and preferred lifestyle (total abstinence on a long-term and persistent basis) and agree to remain abstinent.

In addition, male subjects must refrain from donating sperm for 30 days after the last dose of study treatment.

Male subjects with a pregnant or breastfeeding partner must agree to remain abstinent from penile-vaginal intercourse or use a male condom during each episode of penile penetration for 30 days after the last dose of study treatment.

This document is confidential.

Female Subjects

Female subjects of childbearing potential are eligible to participate if they agree to use two highly effective methods of contraception consistently and correctly as described in Table 9.

Table 9. Highly Effective Contraceptive Methods

**Highly Effective Contraceptive Methods That Are User Dependent**[a]<br>*Failure rate of <1% per year when used consistently and correctly.*
Combined (estrogen and progestogen containing) hormonal contraception associated with inhibition of ovulation[b]<br>• Oral<br>• Intravaginal<br>• Transdermal
Progestogen only hormonal contraception associated with inhibition of ovulation<br>• Oral<br>• Injectable
**Highly Effective Contraceptive Methods That Are User Independent**[a]
Implantable progestogen only hormonal contraception associated with inhibition of ovulation[b]<br>• Intrauterine device<br>• Intrauterine hormone-releasing system<br>• Bilateral tubal occlusion
**Vasectomized Partner**<br>*A vasectomized partner is a highly effective contraception method if the partner is the sole male sexual partner of the WOCBP and the absence of sperm has been confirmed. If not, an additional highly effective method of contraception should be used.*
**Sexual Abstinence**<br>*Sexual abstinence is considered a highly effective method only if defined as refraining from heterosexual intercourse during the treatment period and continuing for 30 days after the End of Study visit. The reliability of sexual abstinence needs to be evaluated in relation to the duration of the study and the preferred and usual lifestyle of the subject.*
NOTES:<br>• Typical use failure rates may differ from those when used consistently and correctly. Use should be consistent with local regulations regarding the use of contraceptive methods for subjects participating in clinical studies.<br>• Hormonal contraception may be susceptible to interaction with the study treatment, which may reduce the efficacy of the contraceptive method. In this case, 2 highly effective methods of contraception should be utilized during the treatment period and for at least 30 days after the last dose of study treatment.

Pregnancy Testing:

- WOCBP should only be included after a confirmed menstrual period and a negative highly sensitive urine or serum pregnancy test (a urine test will be provided by the Central Laboratory)
- Pregnancy testing will be performed at Day 12, Day 38, and whenever a menstrual cycle is missed or when pregnancy is otherwise suspected up to 30 days after the last dose of study treatment.

Collection of Pregnancy Information:

Male Subjects With Partners Who Become Pregnant

The Investigator will attempt to collect pregnancy information on any male subject's female partner who becomes pregnant while the male subject is in this study up to 30 days following the last dose of study treatment.

After obtaining the necessary signed informed consent from the pregnant female partner directly, the Investigator will record pregnancy information on the appropriate form and submit it to the Sponsor within 24 hours of learning of the partner's pregnancy. The female partner will also be followed to determine the outcome of the pregnancy. Information on the status of the mother and child will be forwarded to the Sponsor. Generally, the follow-up will be no longer than 6 to 8 weeks following the estimated delivery date. Any termination of the pregnancy will be reported regardless of fetal status (presence or absence of anomalies) or indication for the procedure.

Female Subjects Who Become Pregnant

The Investigator will collect pregnancy information on any female subject who becomes pregnant up to 30 days after the last dose of study treatment. Information will be recorded on the appropriate form and submitted to the Sponsor within 24 hours of learning of a subject's pregnancy. The subject will be followed to determine the outcome of the pregnancy. The Investigator will collect follow-up information on the subject and the neonate and the information will be forwarded to the Sponsor. Generally, follow-up will not be required for longer than 6 to 8 weeks beyond the estimated delivery date. Any termination of pregnancy will be reported, regardless of fetal status (presence or absence of anomalies) or indication for the procedure.

While pregnancy itself is not considered to be an AE or SAE, any pregnancy complication or elective termination of a pregnancy will be reported as an AE or SAE. A spontaneous abortion is always considered to be an SAE and will be reported as such. Any post-study pregnancy related SAE considered reasonably related to the study treatment by the Investigator will be reported to the Sponsor as described in Section 12.6.4. While the Investigator is not obligated to actively seek this information in former study subjects, he or she may learn of an SAE through spontaneous reporting.

APPENDIX 2. BRISTOL STOOL CHART

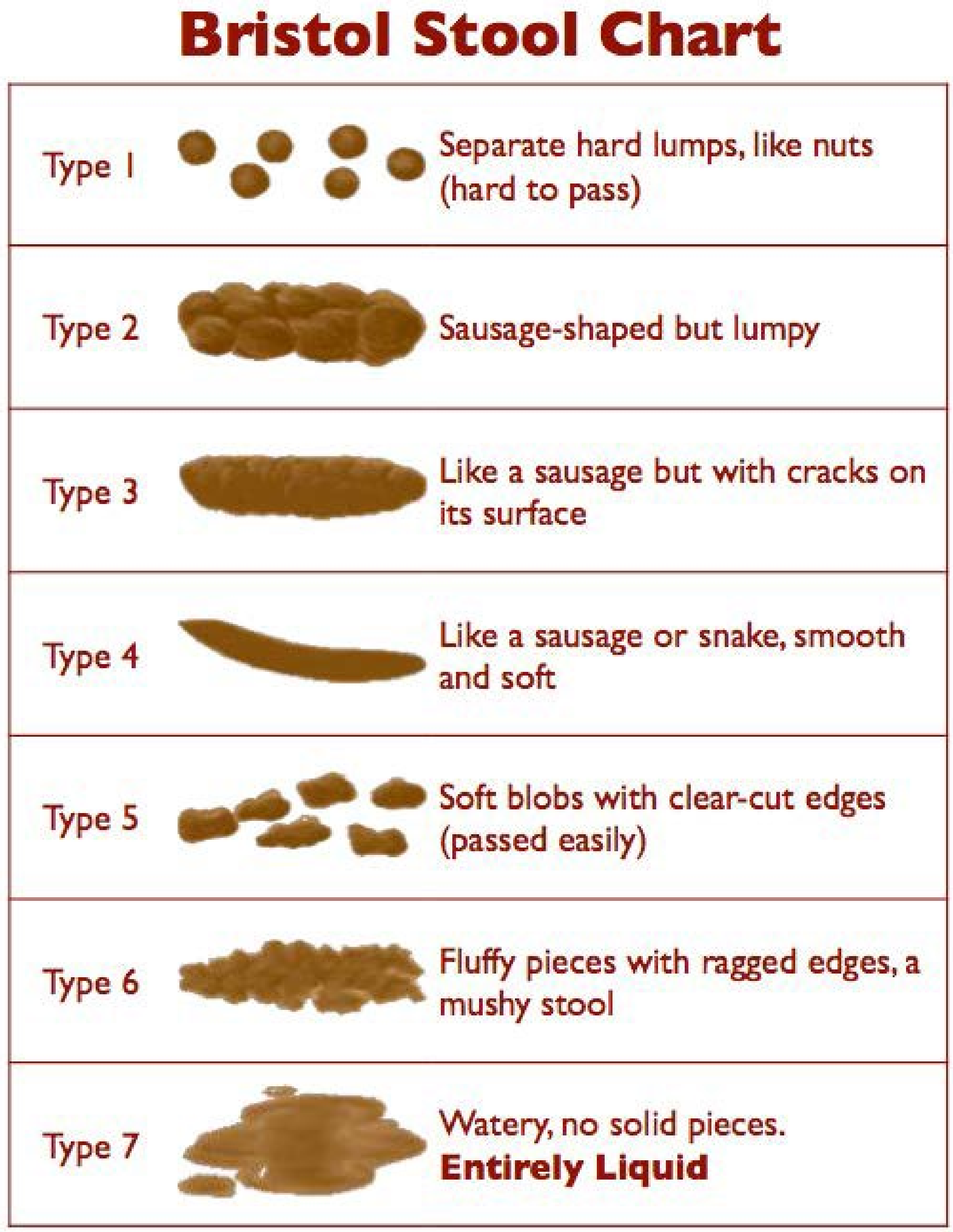

Supplement: Supplementary appendix [file NIHMS2154798-supplement-Supplementary_appendix.pdf]
